# Silica-coated magnetic-nanoparticle-induced cytotoxicity is reduced in microglia by glutathione and citrate identified using integrated omics

**DOI:** 10.1186/s12989-021-00433-y

**Published:** 2021-11-25

**Authors:** Tae Hwan Shin, Balachandran Manavalan, Da Yeon Lee, Shaherin Basith, Chan Seo, Man Jeong Paik, Sang-Wook Kim, Haewoon Seo, Ju Yeon Lee, Jin Young Kim, A Young Kim, Jee Min Chung, Eun Joo Baik, Seong Ho Kang, Dong-Kug Choi, Yup Kang, M. Maral Mouradian, Gwang Lee

**Affiliations:** 1 Department of Physiology, Ajou University School of Medicine, 206 World cup-ro, Suwon, 16499 Republic of Korea; 2 College of Pharmacy, Sunchon National University, 255 Jungang-ro, Suncheon, 57922 Republic of Korea; 3 Department of Molecular Science and Technology, Ajou University, 206 World cup-ro, Suwon, 16499 Republic of Korea; 4 Research Center of Bioconvergence Analysis, Korea Basic Science Institute, 162 Yeongudanji-ro, Cheongju, 28119 Republic of Korea; 5 Department of Chemistry, Graduate School, Kyung Hee University, Yongin-si, Gyeonggi-do 17104 Republic of Korea; 6 Department of Applied Chemistry and Institute of Natural Sciences, Kyung Hee University, Yongin-si, Gyeonggi-do 17104 Republic of Korea; 7 Department of Biotechnology, College of Biomedical and Health Science, Konkuk University, 268 Chungwondaero, Chungju, 27478 Republic of Korea; 8 RWJMS Institute for Neurological Therapeutics, Rutgers Biomedical and Health Sciences, and Department of Neurology, Robert Wood Johnson Medical School, Rutgers University, Piscataway, NJ 08854 USA; 9 Department of Molecular Science and Technology, Ajou University, Suwon-si, Gyeonggi-do 16499 Republic of Korea; 10 Department of Physiology, Ajou University School of Medicine, Suwon-si, Gyeonggi-do 16499 Republic of Korea

**Keywords:** Silica-coated magnetic nanoparticles, Nanotoxicity, Integrated omics, Microglia, Machine learning

## Abstract

**Background:**

Nanoparticles have been utilized in brain research and therapeutics, including imaging, diagnosis, and drug delivery, owing to their versatile properties compared to bulk materials. However, exposure to nanoparticles leads to their accumulation in the brain, but drug development to counteract this nanotoxicity remains challenging. To date, concerns have risen about the potential toxicity to the brain associated with nanoparticles exposure via penetration of the brain blood barrier to address this issue.

**Methods:**

Here the effect of silica-coated-magnetic nanoparticles containing the rhodamine B isothiocyanate dye [MNPs@SiO_2_(RITC)] were assessed on microglia through toxicological investigation, including biological analysis and integration of transcriptomics, proteomics, and metabolomics. MNPs@SiO_2_(RITC)-induced biological changes, such as morphology, generation of reactive oxygen species, intracellular accumulation of MNPs@SiO_2_(RITC) using transmission electron microscopy, and glucose uptake efficiency, were analyzed in BV2 murine microglial cells. Each omics data was collected via RNA-sequencing-based transcriptome analysis, liquid chromatography-tandem mass spectrometry-based proteome analysis, and gas chromatography- tandem mass spectrometry-based metabolome analysis. The three omics datasets were integrated and generated as a single network using a machine learning algorithm. Nineteen compounds were screened and predicted their effects on nanotoxicity within the triple-omics network.

**Results:**

Intracellular reactive oxygen species production, an inflammatory response, and morphological activation of cells were greater, but glucose uptake was lower in MNPs@SiO_2_(RITC)-treated BV2 microglia and primary rat microglia in a dose-dependent manner. Expression of 121 genes (from 41,214 identified genes), and levels of 45 proteins (from 5918 identified proteins) and 17 metabolites (from 47 identified metabolites) related to the above phenomena changed in MNPs@SiO_2_(RITC)-treated microglia. A combination of glutathione and citrate attenuated nanotoxicity induced by MNPs@SiO_2_(RITC) and ten other nanoparticles in vitro and in the murine brain, protecting mostly the hippocampus and thalamus.

**Conclusions:**

Combination of glutathione and citrate can be one of the candidates for nanotoxicity alleviating drug against MNPs@SiO_2_(RITC) induced detrimental effect, including elevation of intracellular reactive oxygen species level, activation of microglia, and reduction in glucose uptake efficiency. In addition, our findings indicate that an integrated triple omics approach provides useful and sensitive toxicological assessment for nanoparticles and screening of drug for nanotoxicity.

**Graphical Abstract:**

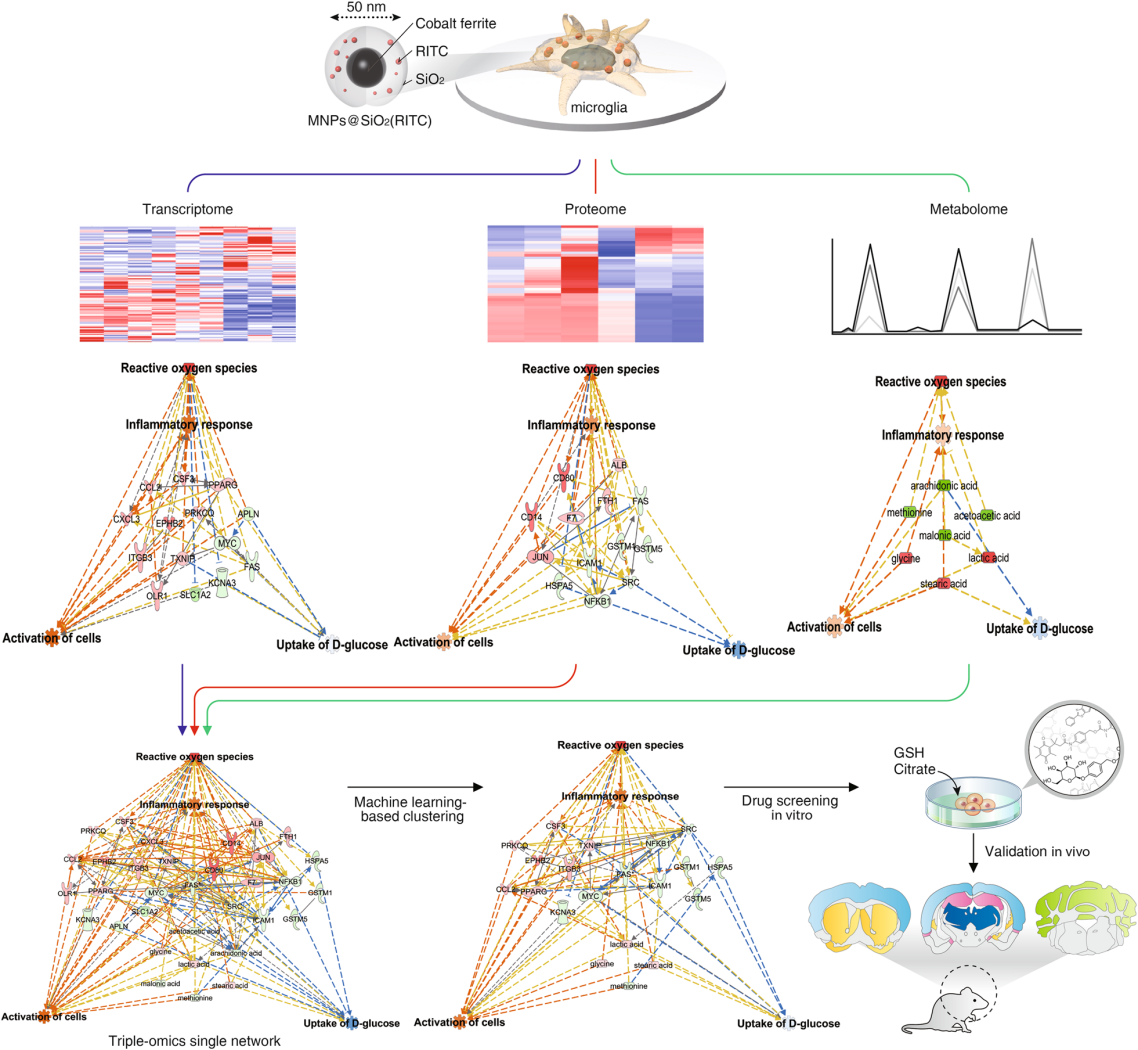

**Supplementary Information:**

The online version contains supplementary material available at 10.1186/s12989-021-00433-y.

## Background

Broad applications of magnetic nanoparticles (MNPs)—and those coated with biocompatible silica and polymers—include contrast agents for magnetic resonance imaging, in vivo tracers, transfection agents, and drug delivery tools [1–5]. Nanoparticles (NPs) have also enabled various technological advances in biotechnology, materials science, engineering, and biomedicine [6–9]. However, there are concerns about their safety owing to the large surface area-to-volume ratio and nanoscale size effects, making them more reactive than bulk-sized materials [10–12]. Nanomaterial-induced toxicity (nanotoxicity) is mostly mediated by redox imbalance, including reactive oxygen species (ROS) production, mitochondrial dysfunction, and energy metabolism dysfunction [13–20]. However, detailed biological evaluations for the mechanisms of the redox imbalance are still limited [5, 21]. Therefore, an increasing number of studies are focusing on reducing nanotoxicity and identifying the relevant mechanisms [22, 23].

Nanotoxicity to the brain is an increasing concern that is highly related to ROS-induced neurodegeneration [24, 25]. Some NPs, such as polysorbate 80–coated poly(*n*-butyl cyanoacrylate) NPs, MNPs, and silica-coated MNPs containing the rhodamine B isothiocyanate dye [MNPs@SiO_2_(RITC)], penetrate the brain through an active transport mechanism and diffusion without disrupting the blood–brain barrier (BBB) [26–29]. BBB permeability varies by brain region, and the cerebellum, hippocampus, and thalamus are reported to be leakier than other regions due to looser junctional arrangement with differences in junctional protein expression and heterogeneity in distribution of cerebral endothelial cells, pericytes, and astrocytes, of which the BBB is made of [30–32]. Moreover, heterogeneous nanotoxicity and NP accumulation in the brain have been reported [8, 33].

Microglia regulate immune homeostasis of the brain and constitute ~ 5–20% of the total glial cell population in the central nervous system [34]. They are capable of activation by NPs, and several reports have explained the nature and underlying cellular mechanisms of neuronanotoxicity for microglia in the brain [33, 35, 36]. Activation with concomitant morphological changes, such as assuming a round shape and swelling, are a distinctive property of microglia [37]. Lipopolysaccharide (LPS), an outer membrane component of gram-negative bacteria that activates innate immune cells, is a well-known inducer of microglial activation mainly by binding to toll-like receptor 4 [38]. Peripheral administration and injection of LPS directly into the central nervous system induce immune responses in the brain [39]. Recent studies have shown that intravenously administered LPS induces BBB disruption [40] and 0.025% of the overall LPS dose entered brain parenchyma in a mouse model [41]. Thus, LPS is often used as positive control for microglial activation in in vitro and in vivo studies [42, 43]. Moreover, as microglia are one of the major targets for brain-accumulated nanomaterials, their activation induced by nanomaterials can be compared with that promoted by LPS.

Omics analysis involves collective quantification and characterization of an entire set of biomolecules, such as genomics, transcriptomics, proteomics, and metabolomics, which are used in various applications for nanotoxicity evaluation [44–47]. However, a single omics type cannot reveal many complex biological events and interconnected molecular pathways in biological phenomena [48, 49]. To assess nanotoxicity, integrated omics offers a more comprehensive and precise analysis—as compared to classic methods for nanotoxicity detection or a single-omics analysis—by compensating for the weaknesses in each omics. The present study employed this broad approach with the goal to identify agents that can mitigate nanotoxicity.

## Methods

### Nanomaterials

MNPs@SiO_2_(RITC) that are comprised of a ~ 9 nm cobalt ferrite (CoFe_2_O_3_) core and a RITC-encompassed silica shell [1] were purchased from BITERIALS (Seoul, South Korea). Zeta potential of MNPs@SiO_2_(RITC) were previously reported as − 40 to − 30 mV [1, 50]. X-ray diffraction analysis on a High Power X-Ray Diffractometer (Ultima III, Rigaku, Japan) analyzed the structure of MNPs@SiO_2_(RITC), which showed specific patterns of cobalt ferrite; (220) at 30°, (311) at 36°, (400) at 44°, (511) at 57°, and (440) at 64°. The broad peak between 20° and 40° indicated amorphous silica beads [25]. Additionally, we validated whether the non-specific biological effects of MNPs@SiO_2_(RITC) treatment were induced by the silica shell rather than the CoFe_2_O_4_ core in HEK293 cells because the silica is in the periphery of MNPs@SiO_2_(RITC) and directly interacts with biological substances [14, 20, 51, 52]. Ag NPs, Au NPs, and CdSe QDs were generated as in previous studies [53, 54]. In the case of CdSe QDs, the absorption and emission of QDs were analyzed using PDA S‐3100 UV/Vis spectrophotometer (Scinco, Korea) and FP‐6500 fluorescence spectrometer (Jasco, Japan). Polystyrene micro beads (2 μm and 100 nm in diameter) and UPM [National Institute of Standards and Technology (NIST)-1648A], were purchased from Sigma-Aldrich. Thirty-nanometre diameter SiO_2_ NPs, TiO_2_ NPs, and MWCNTs were purchased from US Research Nanomaterials.

### Isolation and culture of primary microglia and neuronal cells

Primary rat microglia were isolated from Sprague–Dawley rats (1-day-old). Briefly, rat brains were divided into the cortex and midbrain and homogenized in Minimum Essential Medium (MEM, Gibco, USA). The cell fractions were plated and cultured in a dish containing MEM supplemented with 10% fetal bovine serum (Gibco, USA), 100 units/ml penicillin, and 100 ng/µl streptomycin (Gibco, USA) and incubated in a 5% humidified CO_2_ chamber at 37 °C. Primary rat microglia and neuronal cells were separated by the ‘shaking off’ method [55]. The murine microglial cell line BV2 was chosen as the in vitro model due to its well described nanotoxicological profile [25, 56, 57]. These cells were provided by Dr. K. Suk (Kyung-Pook National University, Daegu, Korea) and were cultured under the same conditions as primary rat microglia. Purity of the primary rat microglia was verified by flow-cytometric staining with specific antibodies to OX-42. Neuronal cells were verified by immunocytochemistry with specific antibodies to neuronal nuclei (NeuN) for staining of cortical neurons or antibodies to tyrosine hydroxylase (TH) for visualization of dopaminergic neurons.

### Immunoblotting

For protein samples from in vitro experiments, microglia were seeded at a density of 2 × 10^5^ cells/well in 6-well plates and treated with 0.01 or 0.1 µg/µl MNPs@SiO_2_(RITC) for 12 h. Cells were lysed in RIPA buffer (Thermo Fisher Scientific, USA). For protein samples from in vivo experiments, mouse brains were dissected into the cortex, striatum, cerebellum, hippocampus, and thalamus lysed in 10% Triton X-100 RIPA buffer. Lysates were homogenized, incubated at 4 °C for 1 h. The samples were centrifuged at 14,000 g for 15 min at 4 °C and supernatants were collected. Protein concentrations in the supernatants were determined with the BCA Kit (Thermo Fisher Scientific, USA). Next, proteins were divided using SDS-PAGE and transferred onto nitrocellulose membranes. The membranes were blocked with 3% skimmed milk for 1 h at room temperature and incubated with a primary antibody overnight at 4 °C. The following primary antibodies were used: an anti-Iba1 (1:2000, Novus Biologicals, USA), anti-CD40 (1:2000, Novus Biologicals, USA), anti-CD11b (1:2000, Abcam, USA), and anti-β-actin (1:5000, Cell Signalling Technology, USA) antibody. Secondary antibodies were used at a dilution of 1:2000 (Santa Cruz Technologies, USA). Enhanced chemiluminescence (ECL, Thermo Scientific, USA) solution was added to the membranes, and luminescence was taken on medical blue X-ray film (AGFA, Belgium) in a dark room.

### Evaluation of glucose uptake efficiency and intra/extra-cellular glucose concentration

To evaluate the efficiency of glucose uptake, microglia were seeded on a coverslip and treated with 0.01 or 0.1 µg/µl MNPs@SiO_2_(RITC) for 12 h. Cells were then incubated with a fluorescent d-glucose analogue, 2-NBDG, at 37 °C for 30 min, and the fluorescence images of 2-DG were acquired using fluorescence microscopy (Axio vert 200 M, Carl Zeiss, Jena, Germany) at the 3D immune system imaging core facility of Ajou University.

Uptake efficiency of glucose was determined using a luminescence-based kit in accordance with the manufacturer’s specifications (Promega, USA). Briefly, microglial cells were treated with 0.01 or 0.1 µg/µl MNPs@SiO_2_(RITC) for 12 h. Cells were washed twice with PBS and 1 mM 2-DG was added. After 10 min, the 2-DG uptake in cells was stopped with an acid detergent solution. pH was neutralized, and the lysates were mixed with glucose-6-phosphate dehydrogenase (G6PDH), nicotinamide adenine dinucleotide phosphate (NADP^+^), ATP, and luciferase. The luminescence was measured using a Synergy 2 luminometer (BioTek, CA), and the images were taken using a ChemiDoc™ Touch Gel Imaging System (Bio-Rad).

To evaluate intra/extra-cellular glucose, microglia were treated with 0.01 or 0.1 µg/µl MNPs@SiO_2_(RITC) and subsequently washed twice with PBS. Cell were lysed with an acid detergent solution and neutralized. Lysates were mixed with glucose dehydrogenase, NADP^+^, ATP, and luciferase. media was 400-fold diluted with PBS for measurement. luminescence was analyzed using a Synergy 2 luminometer (BioTek, CA), and the images were captured using a ChemiDoc™ Touch Gel Imaging System (Bio-Rad).

### Transmission electron microscopy (TEM)

To analyze the changes in MNPs@SiO_2_(RITC)-treated cells, Karnovsky’s fixative solution (Sigma-Aldrich, USA) was used for fixation of MNPs@SiO_2_(RITC)-treated BV2 for 12 h at 4 °C. Cells were sequentially washed with 0.1 M cacodylate buffer, pH 7.4, and post-fixed with 1% (v/v) osmium tetroxide (Polysciences, USA) in a 0.1 M cacodylate buffer for 2 h at room temperature. Samples were dehydrated with graded ethanol solutions (50–100%), infiltrated with propylene oxide, and embedded in Epon Mixture (Polysciences, USA). Samples were incubated at 35 °C for 6 h, at 45 °C for 12 h, and at 60 °C for 24 h. Blocks were sectioned with an ultramicrotome (Reichert-Jung, Bayreuth, Germany). Sections were double-stained with 6% uranyl acetate for 20 min (EMS, USA) and lead citrate for 10 min (Thermo Fisher Scientific, USA) for contrast staining. Images were acquired via a SIGMA500 (Zeiss, Germany) transmission electron microscope at the 3D immune system imaging core facility of Ajou University. Particle number, vesicle size, mitochondrial number and size were analyzed using Zen blue 2.3 image analysis module (Zeiss, Germany).

### Proteome sample preparation

BV2 cells were treated with 0.01 and 0.1 µg/µl MNPs@SiO_2_(RITC) for 12 h. Cells were lysed with RIPA buffer. Lysates were cleared by centrifugation, and the samples were denatured with 8 M urea and reduced with 5 mM tris(2-carboxyethyl) phosphine at room temperature for 10 and 60 min. Samples were alkylated with 15 mM iodoacetamide in the dark for 60 min at RT, and the buffer was replaced with 200 mM triethylammonium bicarbonate buffer. The total amount of proteins was measured by the Qubit Assay (Thermo Fisher Scientific, USA) following the manufacturer’s protocol. Three biological replicate samples of control and of 0.01 and 0.1 µg/µl MNPs@SiO_2_(RITC)-treated groups were pooled as the respective groups. Proteins were digested with trypsin at 37 °C for 16 h (Promega, USA). Total peptide concentration was re-quantified by the Qubit Assay (Thermo Fisher Scientific, USA) following the manufacturer’s protocol. Each sample of 100 µg was divided into two fractions. Samples were individually labelled using TMT-126 and 127 (Control), TMT-128 and -129 [0.01 µg/µl MNPs@SiO_2_(RITC)–treated group], and TMT-130 and -131 [0.1 µg/µl MNPs@SiO_2_(RITC)–treated group] following the manufacturer’s protocol. An aqueous hydroxylamine solution (5% w/v) was used for quenching the reaction. The six TMT-labelled samples were then combined, speed-vacuum dried, and then resuspended in 30 μl of water for fractionation by high-pH reversed-phase liquid chromatography [58].

### Liquid chromatography with tandem mass spectrometry (LC–MS/MS) analysis for proteome samples

TMT-labeled peptides were quantified using an Easy nLC 1200 (Thermo Fisher scientific, USA) combined with an Orbitrap Fusion Lumos mass spectrometer (Thermo Fisher Scientific, USA), and a nano-electrospray source (Thermo Fisher Scientific, USA) [59]. Before separation, Peptides were trapped with a 75 μm × 2 cm C_18_ precolumn (Thermo Fisher Scientific, USA). analytical C_18_ column (75 μm × 50 cm, Thermo Fisher Scientific, USA) was used and the flow rate was 300 nl/min. Zero to eighty % acetonitrile containing 0.1% formic acid was used as mobile phases A and B, respectively. The mobile phase gradient was used 6% phase B for 1 min and was elevated to 25% phase B for 75 min, to 40% phase B for 15 min, to 100% phase B for 1 min, and maintained at 100% phase B for 8 min, and 2% phase B for additional 5 min. The column was maintained with 2% phase B for 15 min. The 1900 V voltage was used to generate an electrospray. For chromatographic separation, the mass spectrometer was conducted in data-dependent mode and automatically 3 s cycle time switching between MS1 and MS2.

### Gas chromatography with MS/MS (GC–MS/MS)

GC-MS/MS analysis was conducted on a gas chromatograph (Shimadzu 2010 Plus, Shimadzu, Kyoto, Japan) combined with a triple quadruple mass spectrometer (Shimadzu TQ 8040, Shimadzu, Kyoto, Japan). A cross-linked capillary column (Ultra-2, 95% methylpolysiloxane-and 5% phenyl-bonded phase; 25 m × 0.20 mm inner diameter, 0.11 μm film thickness, Agilent Technologies, Atlanta, GA, USA) were used for separation. Samples were added in split-injection mode (10:1). The operation temperature was (60 °C for 2 min, elevated to 255 °C at a rate of 25 °C/min, elevated to 300 °C at a rate of 7 °C/min, with a holding 2.5 min). The temperatures were 260 °C for the injector, 300 °C for interface, and 230 °C for ion source were, respectively. Carrier (helium) and collision (argon) gases were used and the electron ionization (EI) mode was adjusted to 70 eV.

### Unsupervised principal component analysis (PCA)

To evaluate the relevance and to trim the triple-omics network (transcriptomics, proteomics, and metabolomics), we performed PCA. Each value in each omics was normalized to Z-scores, so that the data were on a similar scale of changed levels for clustering analysis. The multidimensional Z-score of each gene, protein, and metabolite was transformed into a two-dimensional space, where the first-dimension (PC1) and second dimension (PC2) are linear combinations of original values with a certain weight [60]. To identify the largest cluster in the PCA plot, we applied the unsupervised K-means clustering algorithm implemented in *Scikit*, a machine learning tool in Python [61]. This method takes two-dimensional data (from PCA) and tries to group them into ‘K’: number of clusters, where K is user defined. The pseudo code for the K-means algorithm was as follows:
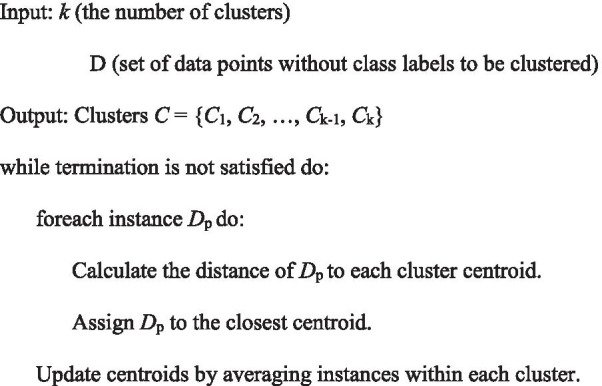


To test the robustness of the clusters, we also tested the unsupervised *k*-nearest neighbour (*k*-NN) algorithm (instead of *k*-means clustering) implemented in *Scikit* with default parameters (*k* = 3, algorithm = ‘ball_tree’), where k is the number of neighbours, and the algorithm is the choice of neighbours’ search. Of note, we obtained a result similar to the one above, indicating its robustness regardless of the algorithm.

### Evaluation of microglial activation in vivo

This work has received approval for research ethics from Laboratory Animal Research Center of Ajou University Medical Center and proof/certificate of approval is available upon request. All animal experiments were conducted in accordance with experimental protocols approved by the Laboratory Animal Research Center of Ajou University Medical Center (approval no. 2020-0033). The design of the animal study is reported previously [25, 26]. Mice (male ICR, 8 weeks old, Orientbio, Seongnam, Korea) were maintained on a 12 h light/dark cycle with free access to food and water. Four mice per group were used in this study. Another study analyzed the biodistribution and the toxicological impact of MNPs@SiO_2_(RITC) at doses of 25, 50, and 100 mg/kg [26]. The study uncovered a broad tissue distribution of MNPs@SiO_2_(RITC) and brain localization of MNPs@SiO_2_(RITC) without BBB disruption. Moreover, there were no significant adverse manifestations, such as growth, aberrant behaviours, biochemical changes in serum, or histopathology, even at the 100 mg/kg dose. However, based on our in vitro observations, we postulated that there will be subtle toxicity in the brain at the 100 mg/kg dose, which is the maximum concentration in the previous study [26]. Thus, MNPs@SiO_2_(RITC) were injected intraperitoneally in sterile saline at a dose of 100 mg/kg per mouse. GSH and citrate (Sigma-Aldrich, USA) were dissolved in sterile saline, and pH levels were adjusted to 7.4. A mixture of GSH and citrate was injected intraperitoneally at doses of 1000 mg/kg and 200 mg/kg, respectively. Control mice were injected with sterile saline only. The endpoint for analysis was set to 5 days according to a previous biodistribution study [26]. Five days after injection, mice were anaesthetized with urethane (1.2–1.5 g/kg, intraperitoneally), the hearts were rapidly exposed, and the mice were perfused transcardially with paraformaldehyde (PFA). The brains were fixed in PFA for 24 h. Brains were submerged in 30% sucrose and then sectioned at 5 μm and stored at − 80 °C until further analysis.

Fixed, frozen sections were blocked in a 1% bovine serum albumin (Sigma-Aldrich, USA) with 10% donkey serum (Sigma-Aldrich, USA) in PBS at room temperature for 2 h. Tissue sections were stained with a polyclonal goat anti-Iba1 antibody (1:100, Novus biologicals, USA) diluted in 1% donkey serum in PBS with 0.4% Triton X-100 and incubated at 4 °C overnight. The sections were rinsed and washed (3 times for 10 min) in PBS with 0.4% Triton X-100. A secondary antibody was a Thermo Scientific Alexa Fluor 488-conjugated donkey anti-goat IgG antibody at 1:100 dilution in 1% donkey serum in PBS with 0.4% Triton X-100 and was incubated for 2 h at room temperature. The samples were mounted with the Vectashield mounting medium containing DAPI (Vector Laboratories) and coverslips were applied. Immunostained sections were scanned with slide scanner (Axio Scan.Z1, Zeiss, Germany) at the 3D immune system imaging core facility of Ajou University, and regions of interest were viewed via a 40× objective lens under an A1R HD25 confocal microscope (Nikon, Japan) at the 3D immune system imaging core facility of Ajou University. Three-dimensional reconstructions of branch structures were conducted, and their numbers and lengths were determined using Imaris 9.2 software (Bitplane, Zurich, Switzerland).

### Statistical analysis

The results were subjected to one-way analysis of variance (ANOVA) and Tukey’s honestly significant difference (HSD) post hoc test in the IBM-SPSS software (IBM Corp., USA). Differences were considered statistically significant at *p* < 0.05.

## Results

### Characterization of nanoparticles

The nanoparticles used in this study were characterized using TEM, X-ray diffraction analysis, and UV/Vis and fluorescence spectrometry. As determined by TEM, the MNPs@SiO_2_(RITC) and SiO_2_ NPs were found to be 50 nm in diameter (Additional file 1: Fig. S1). Dose-dependent effects on cell viability were analyzed in the BV2 cell line and primary rat microglia with 0–1.0 μg/μl MNPs@SiO_2_(RITC) and 1.0 μg/μl MNPs@SiO_2_(RITC) treatment for 12 h, which reduced viability by approximately 80% in both cell types (Additional file 1: Fig. S2). Ag and Au NPs, and CdSe QDs were characterized regarding their uniformity and size using TEM (Additional file 1: Fig. S3). In the case of CdSe QDs, the absorption and emission of the QDs were also analyzed (Additional file 1: Fig. S4).

### Phenotypic characterization of MNPs@SiO_2_(RITC)-treated microglia in vitro

The efficiency of MNPs@SiO_2_(RITC) uptake was higher in BV2 cells and rat primary microglia than in primary cortical and dopaminergic neurons (Additional file 1: Fig. S5), when any of these cells were treated with 0.01 or 0.1 μg/μl MNPs@SiO_2_(RITC) for 12 h (Additional file 1: Fig. S6). The differences were more pronounced with 0.01 μg/μl MNPs@SiO_2_(RITC) treatment. Moreover, the uptake rate reached a plateau in 0.1 μg/μl MNPs@SiO_2_(RITC)-treated BV2 cells and primary microglia, and viability decreased by ~ 80% among 1.0 μg/μl MNPs@SiO_2_(RITC)–treated BV2 cells and primary microglia after 12 h (Additional file 1: Fig. S2). Therefore, the dose chosen to treat microglia with MNPs@SiO_2_(RITC) in this study ranged from 0.01 to 0.1 µg/µl. Intracellular ROS level increased after 0.1 µg/µl MNPs@SiO_2_(RITC) treatment compared to no-treatment control and 0.01 µg/µl MNPs@SiO_2_(RITC)–treated cells (Additional file 1: Fig. S7).

**Fig. 1:**
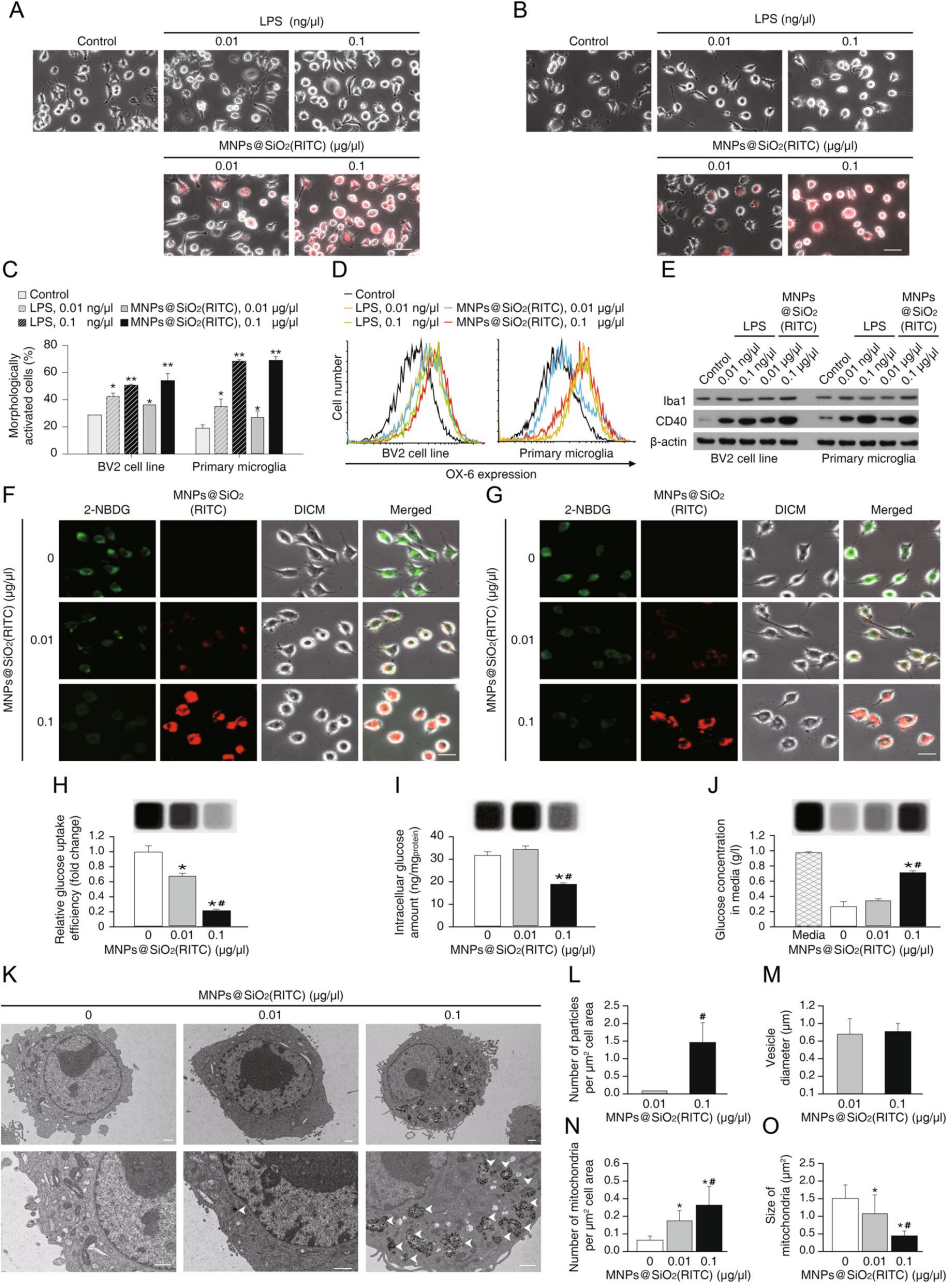
Phenotype of MNPs@SiO_2_(RITC)-treated microglial cells. Morphological analysis of **a** BV2 cells and **b** primary rat microglia. Scale bar = 50 μm. Red: MNPs@SiO_2_(RITC). LPS treatment served as a positive control for activation of microglia. **c** Quantification of swelled and rounded (activated) microglia compared to ramified microglia. **d** Flow-cytometric analysis of BV2 cells and primary microglia for activation marker, OX-6. **e** Immunoblotting analysis for microglia activation markers, Iba1 and CD40. β-actin served as an internal control. Visual analysis of glucose uptake efficiency was performed on 2-[N-(7-nitrobenz-2-oxa-1,3-diazol-4-yl)amino]-2-deoxy-d-glucose (2-NBDG)-treated BV2 cells **f** and primary microglia **g**. Scale bar = 20 µm. Analysis of glucose uptake efficiency with 2-deoxy-d-glucose (2-DG) **h** and glucose amounts in MNPs@SiO_2_(RITC)-treated cells **i** and media **j** were determined from the luminescent images. **k** Representative images of MNPs@SiO_2_(RITC)-treated BV2 cells. Magnified images are in the bottom panels; white arrowheads indicate the location of MNPs@SiO_2_(RITC). Scale bar = 1 μm. The number of particles **l**, vesicle diameter **m**, number of mitochondria **n**, and mitochondrial size **o** were determined. Data represent the means ± standard deviation of three independent experiments. *p < 0.05 versus control, ^#^*p* < 0.05 versus 0.01 μg/μl MNPs@SiO_2_(RITC)-treated cells

Various signs of morphological activation of microglia, including swelling and rounding, were monitored for 12 h after LPS or MNPs@SiO_2_(RITC) treatment (Fig. 1a, b). The morphological characteristics of BV2 cells and primary microglia changed after MNPs@SiO_2_(RITC) or LPS treatment dose-dependently (Fig. 1c). Moreover, expression level of microglial activation markers, including OX-6, Iba1, and CD40, also increased in a dose-dependent manner (Fig. 1d, e). A 1000-fold higher concentration of MNPs@SiO_2_(RITC) than LPS was necessary to trigger microglial activation, and the magnitude of response to MNPs@SiO_2_(RITC) was similar in BV2 cells and primary microglia.

Subsequently, we assessed the effect of 10 other types of nanomaterials (at doses 0.01 and 0.1 μg/μl for 12 h), that are produced from widespread minerals and used in daily life, e.g. 50 nm silica NPs (SiO_2_ NPs), 20 nm silver NPs (Ag NPs), 15 nm gold NPs (Au NPs), cadmium selenide quantum dots (CdSe QDs), 2 μm and 100 nm polystyrene particles (PSs), 40 nm titanium dioxide NPs (TiO_2_ NPs), 30 nm SiO_2_ NPs, urban particulate matter (UPM), and 25 nm outer diameter (OD) multi-walled carbon nanotubes (MWCNTs), on morphological activation and death of microglia (Additional file 1: Fig. S8). Using 50 nm SiO_2_ NPs [same chemical composition and size as the MNPs@SiO_2_(RITC) shell], Ag NPs, Au NPs, and CdSe QDs, resulted in the morphological activation of microglia. Two μm and 100 nm PSs and TiO_2_ NPs caused cell death and were accumulated over cells. Thirty nm SiO_2_ NPs, UPM, and MWCNTs also induced cell death. In particular, the biological effects on microglial cells were similar between MNPs@SiO_2_(RITC) and 50 nm SiO_2_ NPs.

A significant decrease in glucose uptake was observed in MNPs@SiO_2_(RITC)-treated BV2 cells and primary microglia using fluorescent-based analysis, when compared with untreated control cells, and this effect was concentration dependent (Fig. 1f, g). Evaluation of glucose uptake using enzyme-based analysis also showed a similar trend (Fig. 1h). Moreover, the amount of intracellular glucose was significantly lower in MNPs@SiO_2_(RITC)-treated BV2 cells (Fig. 1i), whereas extracellular glucose showed a reverse pattern (Fig. 1j).

To assess changes in cellular organelles after MNPs@SiO_2_(RITC) treatment, we visualized the ultrastructure of BV2 cells by TEM. The distribution of MNPs@SiO_2_(RITC) was clearly visible as black dots, and changes in mitochondrial size and number were most pronounced among all cellular organelles at the 0.1 μg/μl concentration of the MNPs@SiO_2_(RITC) (Fig. 1k). The number of MNPs@SiO_2_(RITC) per unit cell area was determined, and there was a ~ 14-fold difference between 0.01 and 0.1 μg/μl MNPs@SiO_2_(RITC)–treated BV2 cells (Fig. 1l). The diameter of vesicles containing MNPs@SiO_2_(RITC) was ~ 800 nm (Fig. 1m). The number of mitochondria increased ~ fourfold (Fig. 1n), and the cross-sectional area of each mitochondrion decreased by ~ 75% in 0.1 μg/μl MNPs@SiO_2_(RITC)-treated BV2 cells compared to no-treatment control (Fig. 1o). Phenotypically, reduced glucose uptake with increased ROS, inflammatory response, activation of cells, and mitochondrial fission were noted in MNPs@SiO_2_(RITC)-treated microglia.

### ***Transcriptome, proteome, and metabolome analyses of MNPs@SiO***_***2***_***(RITC)-treated BV2 cells***

**Fig. 2:**
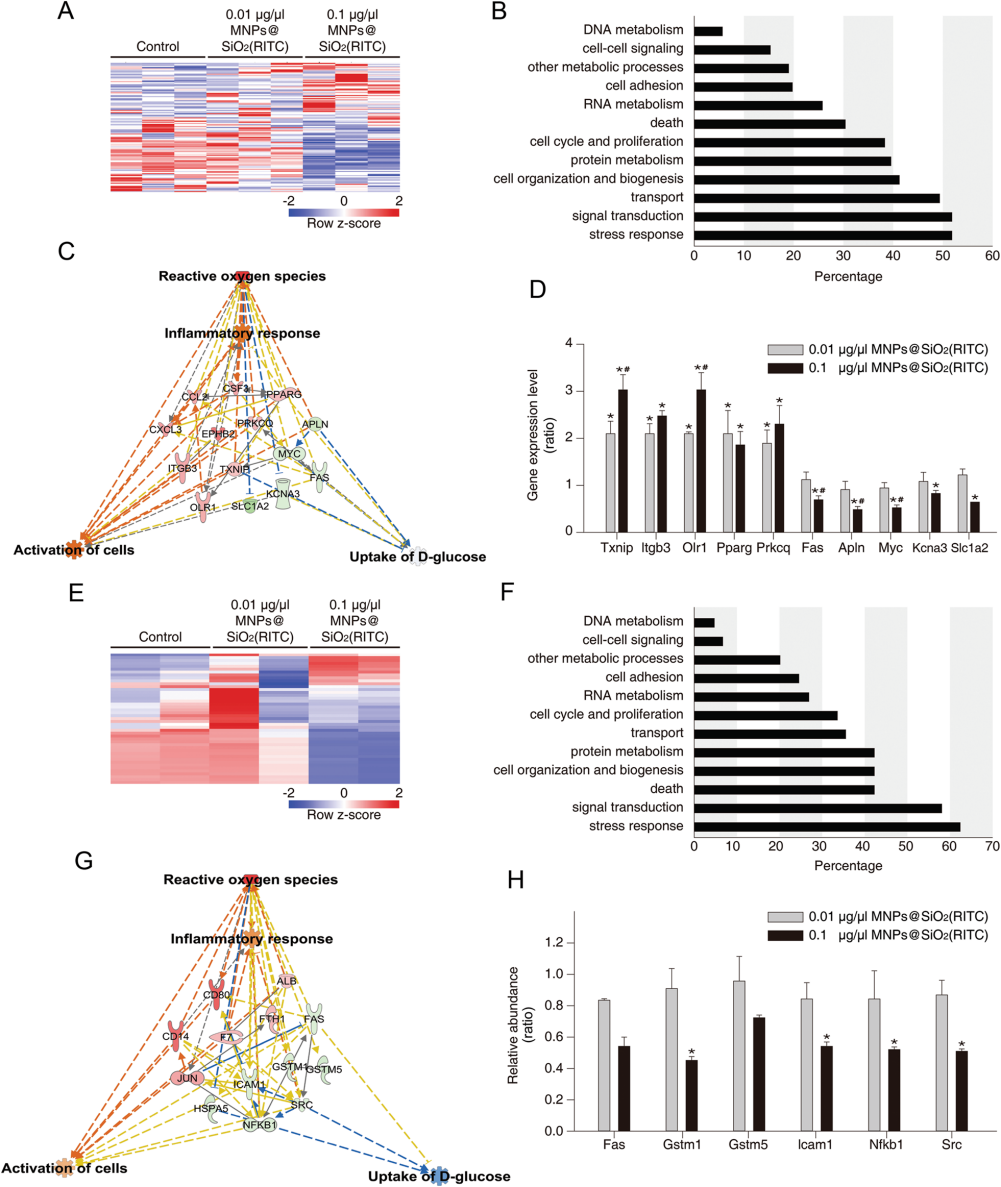
Transcriptomic and proteomic changes in 0.1 µg/µl MNPs@SiO_2_(RITC)-treated BV2 cells. **a** Heat map of 121 genes with altered expression in the RNA-seq analysis. **b** Gene Ontology analysis of the transcriptome for MNPs@SiO_2_(RITC)-treated cells. **c** Analysis of the transcriptomic network with prediction using IPA in MNPs@SiO_2_(RITC)-treated BV2 cells. The analysis involved a fold change cut-off value ± 1.5. Red and green nodes indicate genes that were up-regulated and down-regulated, respectively, compared to the control. Orange and blue arrows indicate prediction of activation and inhibition, respectively. Details for shape and color, which are originated from Ingenuity Systems (http://www.ingenuity.com), are provided in Figs. S9 and S11. **d** qPCR analysis was performed to determine gene expression in each group, with *GAPDH* as an internal control. Data represent means ± standard deviation of three independent experiments. *p < 0.05 versus control and ^#^*p* < 0.05 versus 0.01 µg/µl MNPs@SiO_2_(RITC)-treated cells. **e** Heat map of 45 proteins with altered expression in LC–MS/MS analysis. **f** Gene Ontology analysis of the proteome for MNPs@SiO_2_(RITC)-treated cells. **g** Functional analysis of the proteomic network with prediction using IPA in MNPs@SiO_2_(RITC)–treated cells. The analysis used a fold-change cut-off value ≥  ± 1.5. Details for shape and color are provided in Figs. S9 and S11. **h** The relative abundance levels of Fas, Gstm1, Gstm5, Icam1, Nfkb1, and Src according to LC–MS/MS analysis. Data represent means ± standard deviation of the two TMT ratios. *p < 0.05 versus control

We investigated the transcriptome of BV2 cells by high-throughput RNA sequencing (RNA-seq) after 12 h treatment with 0.01 or 0.1 µg/µl MNPs@SiO_2_(RITC). Higher concentration of MNPs@SiO_2_(RITC) (1.0 µg/µl) resulted in severe cell death and could not be used for omics analyses. We identified 41,214 genes and 4760 genes were differentially expressed (fold change ≥ 1.5 and ≤  − 1.5). Among the genes, we select the most significantly changed gene cluster for analysis. Upon the comparison of 0.01 µg/µl MNPs@SiO_2_(RITC)–treated cells to no-treatment control cells, 31 genes were found to be, and differentially expressed 121 genes were identified in 0.1 µg/µl MNPs@SiO_2_(RITC)-treated cells in the gene cluster (Fig. 2a). Moreover, Gene Ontology analyses revealed that these genes were highly related to a stress response, signal transduction, and transport, among other cellular functions (Fig. 2b). Next, we analyzed the transcriptomic network of the 121 genes by Ingenuity Pathway Analysis (IPA, http://www.ingenuity.com) in 0.01 or 0.1 µg/µl MNPs@SiO_2_(RITC)-treated BV2 cells. This analysis showed that these genes were related to four biological functions, including ROS, inflammatory response, activation of cells, and d-glucose uptake (Additional file 1: Figs. S9 and S10 and Table S1), which were useful for predicting biological functions (Additional file 1: Figs. S11 and S12). Furthermore, canonical pathways and biological functions related to the genes were investigated (Additional file 1: Tables S2 and S3). The transcriptome network was trimmed by providing higher relevance to the biological functions (Additional file 1: Fig. S13), and the prediction of the network uncovered up-regulation of ROS, an inflammatory response, and activation of cells with a decrease in d-glucose uptake (Fig. 2c and Additional file 1: Fig. S14). Among the genes in the network, *Txnip*, *Itgb3*, *Olr1*, *Pparg*, *Prkcq*, *Fas*, *Apln*, *Myc*, *Kcna3*, and *Slc1a2* were analyzed by quantitative PCR (qPCR) and tended to be up- or down-regulated, similar to what was seen in the IPA network (Fig. 2d).

The proteome changes in MNPs@SiO_2_(RITC)-treated BV2 cells were investigated by LC–MS/MS. We identified 5,918 proteins and 482 proteins were differentially expressed (fold change ≥ 1.5 and ≤  − 1.5). Among the proteins, we select the most significantly changed protein expression cluster for analysis. When 0.01 µg/µl MNPs@SiO_2_(RITC)–treated cells were compared to untreated controls, 27 proteins were found to be differentially expressed and differentially expressed 45 proteins were identified in 0.1 µg/µl MNPs@SiO_2_(RITC)–treated cells in the protein expression cluster (Fig. 2e). Gene Ontology analysis revealed that these proteins were related to the stress response and signal transduction (Fig. 2f). Moreover, the proteome network showed that these proteins were highly associated with the same four biological functions identified from the transcriptomic network analysis (Additional file 1: Figs. S15 and S16 and Table S4) and were useful for in silico predictions (Additional file 1: Figs. S17 and S18). Canonical pathways and biological functions were also analyzed among the proteins (Additional file 1: Tables S5 and S6). The proteome network was trimmed by providing higher relevance to the four biological functions (Additional file 1: Fig. S19), and the prediction of the network uncovered up-regulation of the same four functions, like increased ROS, inflammatory response, activation of cells, and down-regulation of d-glucose uptake (Fig. 2g and Additional file 1: Fig. S20). Among the proteins in the network, the relative abundance levels of *Fas*, *Gstm1*, *Gstm5*, *Icam1*, *Nfkb1*, and *Src* tended to be up- or down-regulated, similar to what was observed in the network analysis (Fig. 2h).

**Fig. 3:**
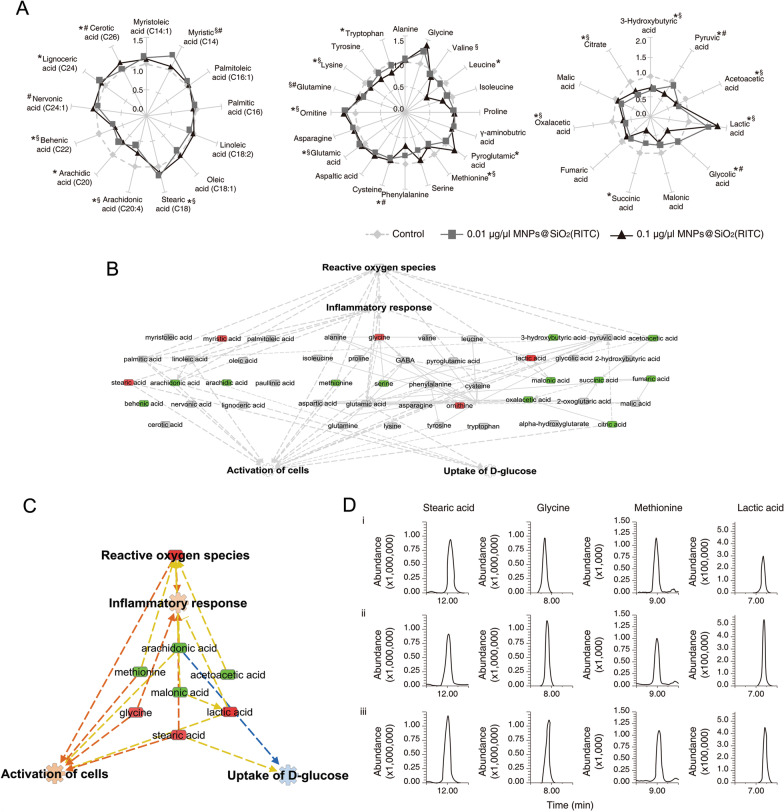
Metabolic disturbances in MNPs@SiO_2_(RITC)-treated BV2 cells. **a** Star patterns for 13 fatty acids (FAs; left), 20 amino acids (AAs; middle), and 11 organic acids (OAs; right) in BV2 cells treated with 0.01 or 0.1 µg/µl MNPs@SiO_2_(RITC) and no-treatment control cells. *p < 0.05 for no-treatment control versus 0.01 µg/µl MNPs@SiO_2_(RITC), ^§^*p* < 0.05 for no-treatment control versus 0.1 µg/µl MNPs@SiO_2_(RITC), and ^#^*p* < 0.05 for 0.01 µg/µl versus 0.1 µg/µl MNPs@SiO_2_(RITC). **b** The metabolomic network of 0.1 µg/µl MNPs@SiO_2_(RITC)–treated BV2 cells using IPA. This analysis employed a fold-change cut-off value ≥  ± 1.2. Details for shape and color are provided in Figs. S9 and S11. **c** The trimmed metabolomic network with prediction for four categories of biological functions. Orange and blue arrows indicate prediction of activation and inhibition, respectively. **d** Representative selected-ion monitoring chromatograms of stearic acid, glycine, methionine, and lactic acid for (i) control, (ii) 0.01, and (iii) 0.01 µg/µl MNPs@SiO_2_(RITC)–treated BV2 cells

We then performed profiling of 13 fatty acids (FAs), 20 amino acids (AAs), and 14 organic acids (OAs) in 0.01 or 0.1 µg/µl MNPs@SiO_2_(RITC)–treated BV2 cells via ethoxycarbonyl (EOC)/methoxime (MO)/tert-butyldimethylsilyl (TBDMS) derivatization and GC–MS/MS analyses (Additional file 1: Table S7). A visual star symbol plot was built using the metabolite composition values of FAs, AAs, and OAs, based on Additional file 1: Table S8 (Fig. 3a). Among the metabolites with altered levels (fold change ≥ 1.2 and ≤  − 1.2), decreased levels of three FAs, two AAs, and seven OAs, and increased levels of two FAs, two AAs, and one OA were noted in 0.1 µg/µl MNPs@SiO_2_(RITC)–treated BV2 cells. Similarly, decreased levels of three FAs, four AAs, and seven OAs, and increased levels of three AAs and one OA were observed in 0.01 µg/µl MNPs@SiO_2_(RITC)–treated cells.

Metabolic profiles were then studied to generate a metabolomic network via IPA (Fig. 3b and Additional file 1: Fig. S21) and to predict up- or down-regulation for the four biological functions (Additional file 1: Fig. S22). Canonical pathways and biological functions were analyzed in relation to the metabolites (Additional file 1: Tables S9 and S10). The network was trimmed by requiring higher relevance to the four biological functions (Additional file 1: Fig. S23), and the network prediction revealed again up-regulation of ROS, an inflammatory response, activation of cells, and down-regulation of d-glucose uptake in 0.1 and 0.01 µg/µl MNPs@SiO_2_(RITC)–treated BV2 cells (Fig. 3c and Additional file 1: Fig. S24). Representative selected-ion monitoring chromatograms of stearic acid, glycine, methionine, and lactic acid in the metabolomic network are presented in Fig. 3d.

### Integrated analysis of the triple-omics network and screening for drugs that alleviate nanotoxicity

**Fig. 4:**
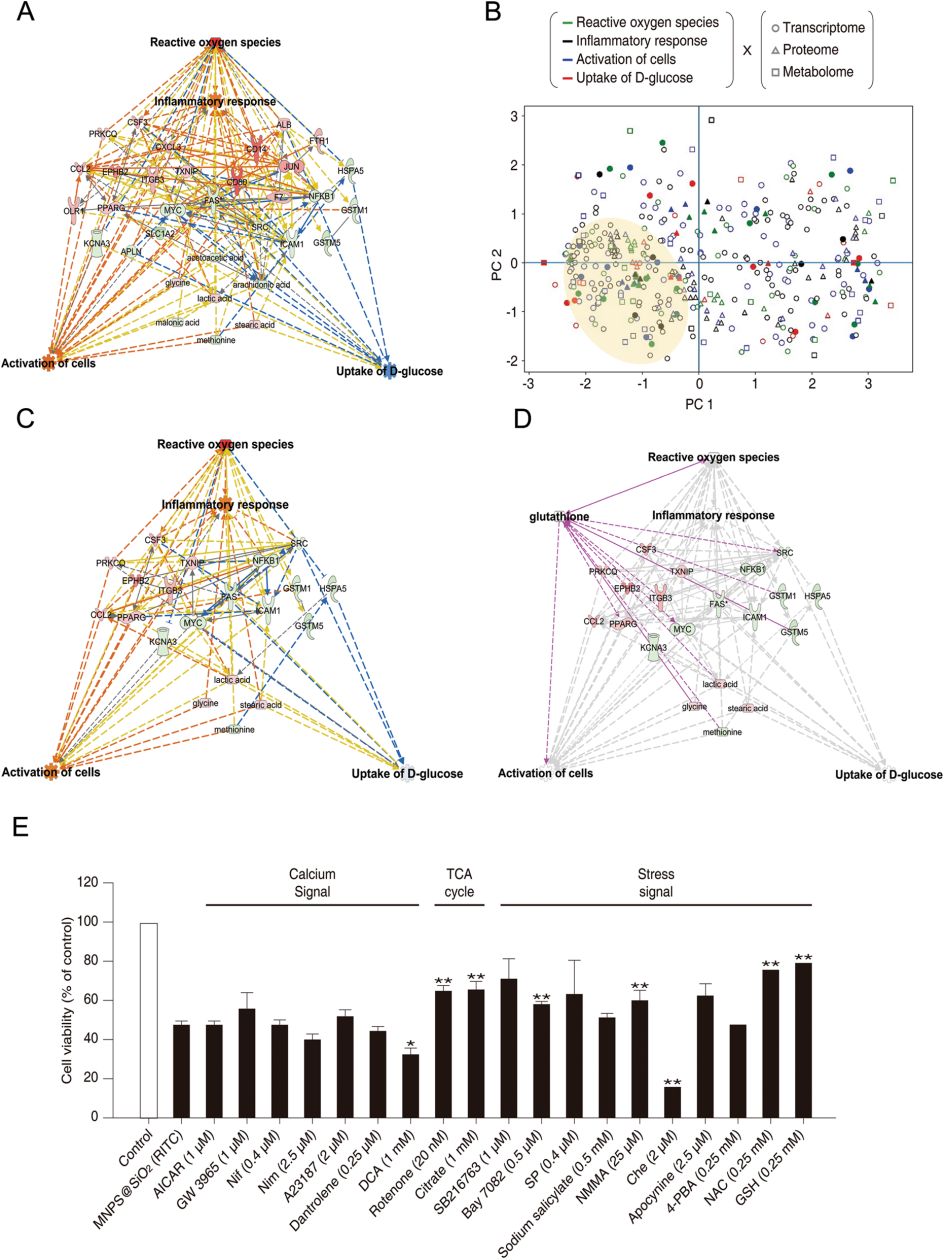
Creation of a triple-omics integrated network in MNPs@SiO_2_(RITC)-treated BV2 cells. **a** The simply merged triple-omics network of 0.1 µg/µl MNPs@SiO_2_(RITC)-treated BV2 cells. Left group: transcriptome, Right group: proteome, Bottom group: metabolome. Down-regulated Fas was shared between the transcriptome and proteome in center. **b** PCA for triple omics against four categories of biological functions. Filled spots indicate items included in the simply merged triple-omics network. The translucent yellow circle indicates the location of the major dominant cluster. **c** The trimmed integrated network for four categories of biological functions. **d** The trimmed triple-omics network with addition of GSH. Factors directly related to GSH are highlighted in magenta. **e** Evaluation of viability in 0.1 µg/µl MNPs@SiO_2_(RITC)–treated BV2 cells co-treated with each of 19 drugs. AICAR: AMP-activated protein kinase activator; GW3965: nonsteroidal liver X receptor agonist; Nif: nifedipine, calcium channel blocker; Nim: nimodipine, voltage-dependent calcium channel blocker; A23187: calcium ionophore; dantrolene: calcium ion release inhibitor; DCA: dichloroacetate, pyruvate dehydrogenase activator; Rotenone: inhibitor of electron transport chain; citrate: a supplement for TCA cycle; SB216763: glycogen synthase kinase 3 inhibitor; Bay7082: inhibitor of inhibitory κB kinase; sodium salicylate: an NFκB inhibitor; SP: SP600125, C-JUN N-terminal kinase inhibitor; NMMA: L-NG-monomethyl-L-arginine, iNOS inhibitor; Che: chelerythrine, pan–protein kinase C inhibitor; apocynin: inhibitor of NADPH oxidase; 4-PBA: 4-phenylbutyrate, chemical chaperone; NAC: N-acetyl cysteine, antioxidant; GSH: glutathione, antioxidant. Data represent means ± SD of triplicate measurements. *p < 0.05 and **p < 0.01 according to one-way ANOVA as compared to control and MNPs@SiO_2_(RITC)-treated BV2 cells, respectively

To compensate for potential weaknesses of each omics, we combined three omics studied above to create a triple-omics network (Additional file 1: Fig. S25) [62–65]. The predictions about four biological functions were consistent with those of the aforementioned single-omics network predictions (Fig. 4a and Additional file 1: Fig. S26). The integrated triple-omics related factors (genes, proteins, and metabolites) and four biological functions (i.e. ROS, inflammatory response, activation of cells, and d-glucose uptake) were subjected to machine learning–based unsupervised analysis using the *k*-NN algorithm. The overall integrated triple-omics biplot, where each data point represents individual omics factor, each involved in one of the four biological functions, is depicted in Fig. 4b. When we carried out the calculations for each group [control, 0.01, and 0.1 µg/µl MNPs@SiO_2_(RITC) treated BV2 cells], such a relation was not observed (Additional file 1: Fig. S27). According to the data distribution, we identified three clusters, one major and two minor ones. Of note, most factors congregated within one major dominant cluster, thus, indicating their stronger relations despite the observed functional differences. To further elucidate the cross-talk points among these triple-omics data and to generate an integrated network, we re-selected genes, proteins, and metabolites in the major cluster in Fig. 4b (indicated by filled circles), constructed an IPA network (Additional file 1: Fig. S28), and carried out a prediction of the trimmed network (Fig. 4c and Additional file 1: Fig. S29). The trimmed integrated networks based on biological functions showed strong relations between the factors and biological functions.

To find drugs that can alleviate nanotoxicity, i.e. to screen 19 drugs with three major categories of agents: those affecting calcium signalling, tricarboxylic acid (TCA) cycle, and stress signaling [66], we added each drug to the network, and the antioxidant glutathione (GSH) was found to be the most effective towards the triple-omics network and related to each factor (Fig. 4d and Additional file 1: Fig. S30). To validate the effect of GSH and to screen other drugs, BV2 cells were treated with 0.1 μg/μl MNPs@SiO_2_(RITC) for 24 h along with one of the three major categories of agents (Fig. 4e). The viability of MNPs@SiO_2_(RITC)-treated BV2 cells was ~ 50% lower compared to control cells. Rotenone, citrate, sodium salicylate, l-NG-monomethyl-l-arginine (NMMA), *N*-acetyl cysteine (NAC), and GSH alleviated nanomaterial-induced cell death, whereas dichloroacetate and chelerythrine reduced cell viability.

In the metabolome profile, we found predominant down-regulation of OAs—except for lactic acid—in MNPs@SiO_2_(RITC)-treated BV2 cells. We examined selected-ion monitoring chromatograms for 10 detected OAs (except for lactic acid, Additional file 1: Fig. S31). The canonical pathway network for the TCA cycle was examined (Additional file 1: Fig. S32), and a prediction of the network showed a reduction in ATP levels followed by down-regulation of OAs (Additional file 1: Fig. S33). In contrast, addition of citrate to the prediction network triggered up-regulation of ATP (Additional file 1: Fig. S34). Thus, we postulated that supplementation of OAs with citrate might alleviate the decrease in energy metabolism caused by MNPs@SiO_2_(RITC) treatment. We chose citrate and GSH for further study because of their nanotoxicity-alleviating activity, biocompatibility, and natural origin.

### Evaluation of protective effects of GSH and citrate against nanotoxicity in vitro and in vivos

**Fig. 5:**
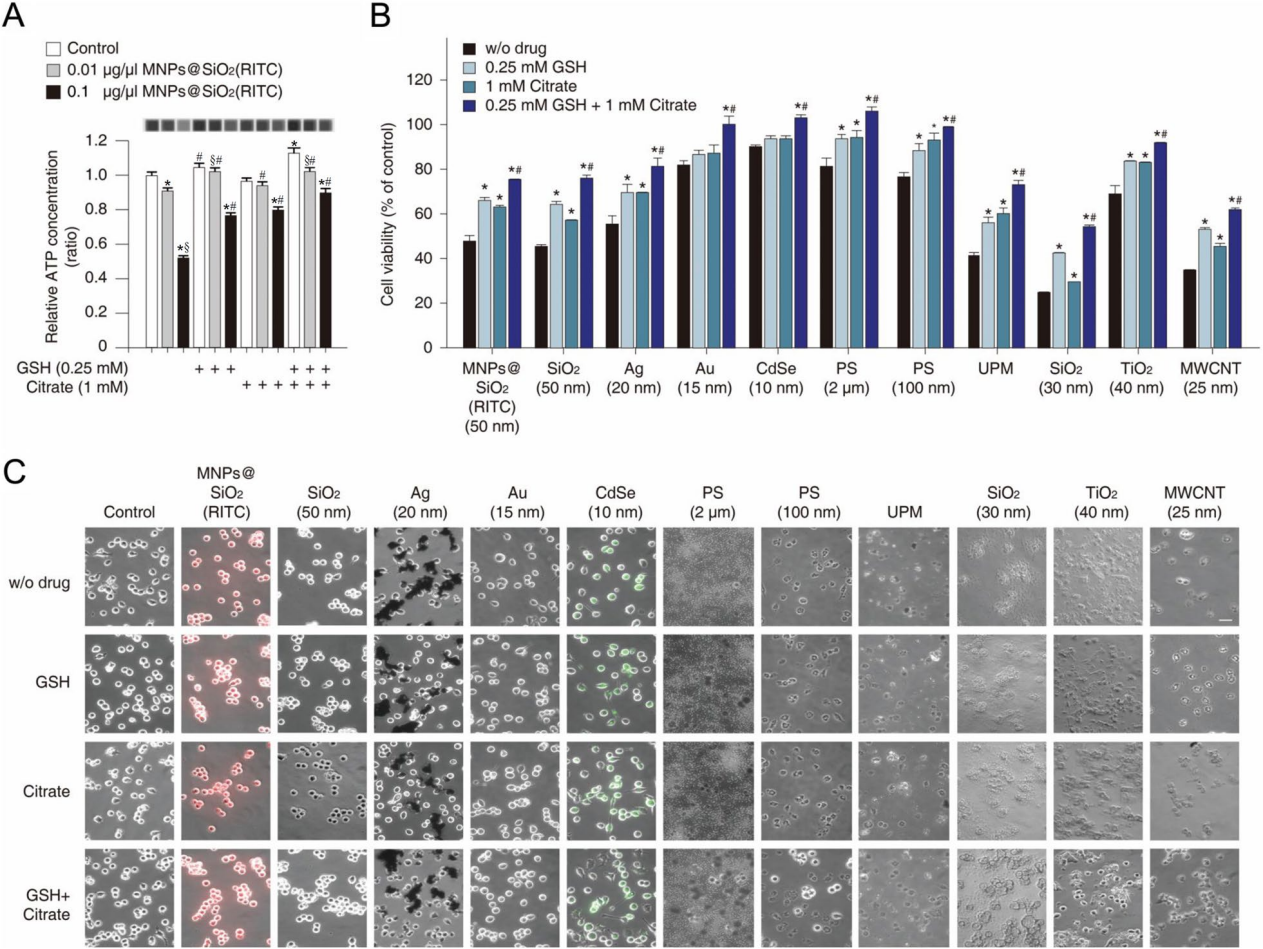
Evaluation of the inhibitory effects of drugs on the toxicity caused by 11 types of nanomaterials towards primary rat microglia. **a** Measurement of intracellular ATP levels in MNPs@SiO_2_(RITC)-treated primary rat microglia in the presence of GSH, citrate, or both. Data represent means ± standard deviation of three independent experiments. *p < 0.05 versus no-treatment control, ^§^*p* < 0.05 versus 0.01 µg/µl dose treatment, and ^#^*p* < 0.05 versus 0.1 µg/µl dose treatment, respectively, according to one-way ANOVA. **b** Cell viability assay for 11 types of nanomaterials (0.1 µg/µl) in primary microglia in the presence of GSH and citrate. Data represent means ± standard deviation of three independent experiments. *p < 0.05 versus w/o drug group, and ^#^*p* < 0.05 versus single treatment of GSH and citrate group, respectively, according to one-way ANOVA. **c** Morphological analysis of nanomaterial-treated primary microglia. Scale bar = 50 μm. Red: MNPs@SiO_2_(RITC). Green: CdSe QDs

We incubated primary microglia with MNPs@SiO_2_(RITC) for 24 h in the presence of each drug or both drugs, and levels of intracellular ATP were evaluated. ATP concentration decreased by ~ 50% in 0.1 µg/µl MNPs@SiO_2_(RITC)-treated cells but recovered by ~ 25% with each drug treatment alone and by ~ 40% with the combination of drugs; Fig. 5a). To study the protective effects of GSH and citrate against the nanotoxicity induced by ten other kinds of nanomaterials, we treated BV2 cells with each nanomaterial (0.1 µg/µl) for 24 h in the presence of each drug or both drugs (Fig. 5b). Among the nanomaterials, SiO_2_ NPs (30 nm) were the most toxic: ~ 75% of cells were dead in this condition. GSH attenuated nanomaterial-induced cell death by ~ 15%, whereas citrate reduced nanomaterial-induced cell death by ~ 10% as compared to NP-challenged untreated cells. The combined (GSH-and-citrate) treatment alleviated this cell death by ~ 25%. Similarly, morphological analysis showed that the nanomaterial-induced cell death was attenuated by the co-treatment with the two drugs (Fig. 5c).

**Fig. 6:**
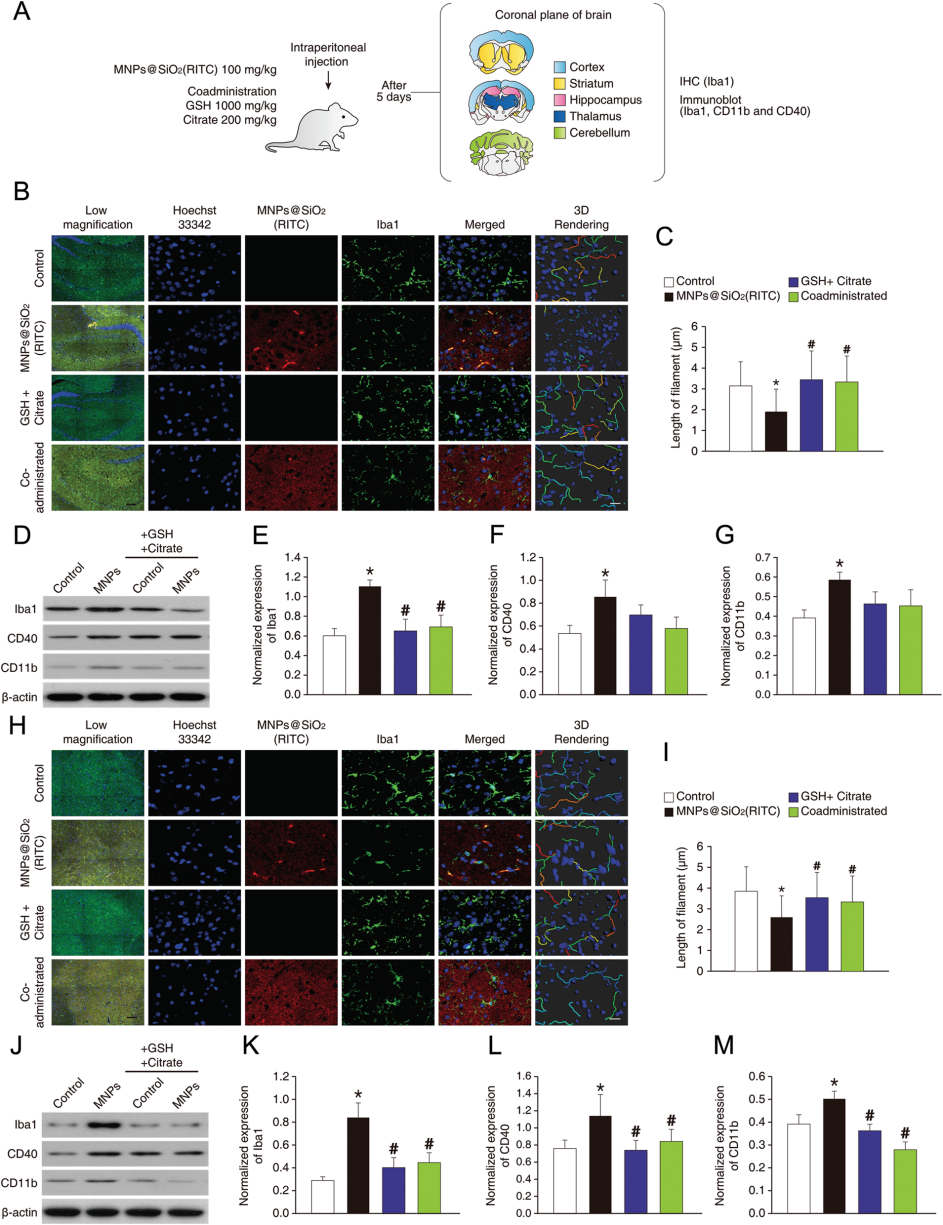
Evaluation of MNPs@SiO_2_(RITC)-induced microglial activation and effects of GSH and citrate in vivo. **a** Schematic of the in vivo experiment. **b** Immunohistochemical analysis of the hippocampal regions of the mouse brain. Low-magnification images are merged with florescence of Hoechst 33,342 (blue), MNPs@SiO_2_(RITC) (red), and Iba1 (green) to show region-specific structure and distribution of MNPs@SiO_2_(RITC). Black scale bar = 100 μm. Magnified images are separated into Hoechst 33,342 (blue), MNPs@SiO_2_(RITC) (red), and Iba1 (green), and Iba1-based 3D rendering images. White scale bar = 10 µm. **c** Determined filament length from 3D rendering images of the hippocampus. **d** Representative immunoblotting data related to microglia activation. β-Actin served as an internal control. Normalized expression of Iba1 **e**, CD40 **f**, and CD11b **g** in hippocampal tissue lysates. **h** Immunohistochemical analysis of the thalamus in the mouse brain. **i** Determined length of a filament from 3D rendering images of thalamic regions. **j** Representative immunoblotting data related to microglia activation. β-Actin was used as an internal control. Normalized expression of Iba1 **k**, CD40 **l**, and CD11b **m** in lysates of the thalamus. Data represent means ± standard error of three independent experiments. *p < 0.05 versus control, ^#^*p* < 0.05 versus MNPs@SiO_2_(RITC)-treated mice according to one-way ANOVA

To assess the potential of MNPs@SiO_2_(RITC) to cause nanotoxicity to microglia, mice were injected intraperitoneally with MNPs@SiO_2_(RITC) at a dose of 100 mg/kg or co-treated with a combination of GSH and citrate for 5 days (Fig. 6a). The brains were divided into the cortex, striatum, cerebellum, hippocampus, and thalamus and analyzed. Distribution of MNPs@SiO_2_(RITC) was homogeneous across the brain, and these NPs accumulated preferentially in Iba1-positive microglia. Moreover, MNPs@SiO_2_(RITC)-positive and Iba1-positive microglia showed morphological evidence of being activated. 3D reconstruction of these cells uncovered changes in the length of filaments of branch structures (Fig. 6b). In the hippocampus of MNPs@SiO_2_(RITC)-challenged mice, lengths of branch structures of microglia were significantly shorter, and this reduction was attenuated by co-administration of GSH and citrate (Fig. 6c). There were no changes in the brains of GSH-and-citrate-treated mice in the absence of MNPs@SiO_2_(RITC) compared to controls. Moreover, there were no significant changes in the number of cells in the brain (Additional file 1: Fig. S35). In immunoblotting assays, Iba1, CD40, and CD11b expression levels were also higher in the brains of MNPs@SiO_2_(RITC)-challenged mice. These increases were attenuated by co-administration of GSH and citrate (Fig. 6d–g). The microglial activation by MNPs@SiO_2_(RITC) and the attenuating effects of GSH and citrate also showed similar trends in the thalamus, cortex, striatum, and cerebellum (Fig. 6h–m and Additional file 1: Figs. S36–S38). The overall levels of microglial activation and the attenuating influence of co-administered GSH and citrate could be ranked as follows: hippocampus > thalamus > cerebellum > striatum > cortex.

## Discussion

To our knowledge, this study is the first systems-biological analysis aimed at nanotoxicological evaluation using integrated triple omics. The resulting single network in microglia was trimmed by machine learning and used for in silico prediction before the screening of relevant drugs. Additionally, we propose that glutathione and citric acid reduce nanotoxicity by mostly targeting ROS production and utilization of energy, respectively.

We detected up-regulation of intracellular ROS, activation of microglia, and mitochondrial fission in MNPs@SiO_2_(RITC)-treated cells. Generally, oxidative stress triggers microglial activation [67], and morphological changes of microglia are an intrinsic and sensitive indicator of changes in the microenvironment of microglia [37]. Moreover, one study showed that mitochondrial fission can accompany ROS production and microglial activation [68]. The correlation between ROS and activation of microglia has been studied in pathological conditions involving up-regulation of ROS, e.g. aging, neurodegenerative diseases, and acute injury of the brain [69–71]. In the present study, we elucidated the relation between ROS and activation of microglia using TEM and integrated omics involving selected genes, proteins, and metabolites with high relevance. Considering the implications to pathological conditions and NP-induced biological changes, NPs also pose a risk of pathologic activation of microglia.

In this study, there were similar responses to MNPs@SiO_2_(RITC) (including increased ROS production, microglial activation, and reduced glucose uptake) between primary rat microglia and the BV2 cell line. Moreover, although the transcriptome, proteome, and metabolome were analyzed in MNPs@SiO_2_(RITC)-treated BV2 cells, the collected data were used to extrapolate the phenomena in primary microglial cells and in an in vivo mouse model. However, some studies have highlighted certain differences between these two cell types in their responses to LPS and TGF-β in transcriptome and proteome analysis [72, 73], namely, primary microglia being more sensitive than BV2 cells. These discrepancies might be explained by complex biological effects of MNPs@SiO_2_(RITC) and their mechanism of action compared to LPS or TGF-β. There are no known proteins or other factors specific for binding to NPs [74], and our results indicate that a 1,000-fold higher concentration of MNPs@SiO_2_(RITC) than LPS is necessary to trigger microglial activation. Moreover, we hypothesized that the action of MNPs@SiO_2_(RITC) in the cell is mostly based on ROS production, which can positively correlate with the efficiency of MNPs@SiO_2_(RITC) uptake. Thus, we found similar efficiency of MNPs@SiO_2_(RITC) uptake between primary microglia and BV2 cells, and as a result, there were similar responses to MNPs@SiO_2_(RITC) between these cells.

Detachment of component, such as cobalt ferrite, RITC, and silica, from MNPs@SiO_2_(RITC) may occur in in vitro and in vivo in complex fluids. However, a previous in vitro study reported that RITC fluorescence intensity of MNPs@SiO_2_(RITC) was reduced by 10% and 60% in the 3rd and 7th passages, respectively, in MNPs@SiO_2_(RITC)-treated human cord blood-derived mesenchymal stem cells, and the reductions were suggested to be due to a dilution effect by cell proliferation [75]. Moreover, cobalt ferrite is more toxic than intact MNPs@SiO_2_(RITC) [15, 51]; however, there was no significant reduction of cell viability in MNPs@SiO_2_(RITC)-treated HEK293 cells over 7 days. In addition, although MNPs@SiO_2_(RITC) localized to various organs in mice treated with MNPs@SiO_2_(RITC) for 4 weeks, an in vivo study revealed no pathological symptoms [26]. Thus, we assume that the MNPs@SiO_2_(RITC) remains intact in complex biological fluid and the detachment of MNPs@SiO_2_(RITC) component may be considered in long-term exposure (> 4 weeks).

Previously, we demonstrated that changes in cells are induced by the silica shell of MNPs@SiO_2_(RITC) rather than the CoFe_2_O_3_ core [15, 20, 51, 52], although the release of free metal ion from SiO_2_ NPs into the cytosol up-regulating intracellular ROS has also been reported [76, 77]. Accordingly, we investigated ROS production induced by MNPs@SiO_2_(RITC) [in comparison with silica NPs that are shall component of MNPs@SiO_2_(RITC)] or by a CoFe_2_O_3_ chemical at similar amount of the CoFe_2_O_3_ core in HEK293 cells treated with 0.1 µg/µl and 1.0 µg/µl MNPs@SiO_2_(RITC). The CoFe_2_O_3_ chemical caused elevated levels of ROS and reduced cell viability at 0.1 µg/µl and 1.0 µg/µl amount treatment, but ROS production was similar between the MNPs@SiO_2_(RITC) and silica NP treated cells at both concentrations 0.1 µg/µl and 1.0 µg/µl for 12, 24, and 48 h. ROS production was induced by MNPs@SiO_2_(RITC) and by silica NPs at 0.01 and 0.1 µg/µl in microglia. Besides, MNPs@SiO_2_(RITC) and silica NPs have similar biological effects [50]. These findings suggest that the elevation in intracellular ROS is due to the silica shell of MNPs@SiO_2_(RITC) and caused a dysfunction of glucose uptake by microglial cells. Furthermore, our results indicate that mitochondrial fission might be triggered by increased ROS generation due to MNPs@SiO_2_(RITC), and as a consequence, reduced ATP production and increased lactate concentration in MNPs@SiO_2_(RITC)-treated cells. Similar findings have been reported in Ag NPs-treated hepatoma cells [78].

We found lower glucose uptake by MNPs@SiO_2_(RITC)-treated microglia with up-regulation of ROS, an inflammatory response, and activation of microglia during the computational prediction and in the actual experimental data. Moreover, one study emphasizes that d-glucose uptake by microglia is regulated by glucose transporter 1 (GLUT1) under inflammation, and the metabolic mechanism is re-programmed by blockage of GLUT1 for regulation of microglial activation and neurodegeneration [79]. By contrast, we found that a reduction in d-glucose uptake correlates inversely with microglia activation, according to in silico prediction by transcriptomic, proteomic, and metabolomic analyses, and there is no significant change in the expression of GLUT1. In this study, ROS turned out to be a major trigger of dysfunctions in microglia, whereas GSH attenuated the reduction in d-glucose uptake. In addition, in canonical signaling, crosstalk between hypoxia-inducible factor (HIF)-1α and NF-κB, which are regulated by ROS, has been suggested [80], with HIF-1α activating NF-κB with consequent increment of GLUT1 expression [81]. However, the GLUT1 expression at the transcriptional level decreased upon HIF-1α inhibition by ROS in NPs-treated cells [82]. On the other hand, other studies reported that NPs treatment induces HIF-1α and NF-κB activation [83, 84]. Thus, interpretation of nanotoxicity based on the canonical pathway remains controversial, and MNPs@SiO_2_(RITC)-induced microglial activation is different from the canonical process of activating microglia. A strategy for alleviating MNPs@SiO_2_(RITC)-induced inflammation should be based on a comprehensive analysis.

Pathway analysis, one of the functional clustering (enrichment) techniques, is a useful tool for omics research. This method facilitates interpretation and hypothesis generation at the overwhelmingly large scale of experimental omics data, according to existing knowledge [14, 85–88]. Thus, researchers may gain insights into heterogeneous phenomena using pathway analysis with in silico prediction.

Pathway analysis of differentially expressed genes, proteins, and metabolites has limitation because the omics data are a snapshot of biological phenomena at a specific time point and cannot fully reflect the dynamics of the phenomena [89]. Moreover, the inter-dependence of biological functions may not be realized due to weak links among biological functions [89]. Additionally, bioinformatic tools for pathway analysis are created mostly on the basis of existing knowledge, hence, novel findings are limited [90, 91]. Thus, a non-canonical pathway cannot be found in pathway analysis, and each omics analysis can be biased, which could be deduced a false positive or negative result. In this study, we trimmed the triple-omics network-related factors by a machine learning–based unsupervised procedure with the *k*-NN algorithm for reducing biases in the analysis. By doing so, we noted stronger relationships of factors despite the observed functional differences. Thus, optimization of an algorithm and combination of analysis tools might be necessary for precise analysis and prediction of biological phenomena.

We detected a reduction in intracellular ATP concentration and an increase of lactate levels (Additional file 1: Tables S7 and S8) in MNPs@SiO_2_(RITC)-treated BV2 cells. Moreover, we examined intracellular accumulation of MNPs@SiO_2_(RITC) and changes in cell ultrastructure using TEM. The most distinguishable changes in cell organelles were detected in mitochondria, which were segregated and showed small cross-sectional area and increased numbers, so called mitochondrial fission, in MNPs@SiO_2_(RITC)-treated BV2 cells. Some researchers demonstrated that mitochondrial fission can take place during oxidative damage, metabolic change, and other pathological conditions [92]. Moreover, in the mitochondrial fission state, cells utilise aerobic glycolysis, which generates ATP by converting glucose to lactate, in accordance with lower mitochondrial respiratory function for utilizing the TCA cycle [93]. Thus, due to mitochondrial fission by MNPs@SiO_2_(RITC)-induced ROS, the level of lactate was higher in MNPs@SiO_2_(RITC)-treated BV2 cells.

GSH and citrate, which we found in the triple-omics network, efficiently reversed the nanotoxicity induced by NPs of various kinds and sizes. GSH is a natural antioxidant that regulates cellular redox homeostasis [94]. Citrate is a natural compound and can serve as a nutritional supplement for the cell as a TCA cycle intermediate [95]. Moreover, GSH and citrate have a chemical property of chelating metal ions [96], whereby they can reduce up-regulation of intracellular calcium ion, which can induce apoptosis, while citrate can chelate an NP-derived toxic metal ion. We studied the protective effect against nanotoxicity in MNPs@SiO_2_(RITC)-treated BV2 cells using modulators affecting stress signalling, calcium signalling, lipid metabolism, the TCA cycle, and oxidative stress. Moreover, using triple-omics analysis in MNPs@SiO_2_(RITC)-treated BV2 cells, we found that ROS is highly related with genes, proteins, and metabolites that are involved in inflammatory response, activation of cells, and uptake of d-glucose in MNPs@SiO_2_(RITC) treated microglia. We also found intracellular ATP levels to be decreased in MNPs@SiO_2_(RITC)-treated microglia, and TCA cycle energy metabolism was impaired by reduction of organic acids. In addition, GSH and citrate were identified from the triple-omics analysis, and their expected major mechanisms of action were found to be reduction of ROS production and supplementation of energy source, respectively. In addition, although we examine the nanotoxicity alleviation effect of intraperitoneal administration of glutathione and citrate in alleviating nanotoxicity in an in vivo mouse model, these compounds are orally bioavailable, making their broad application feasible [97, 98]. We also suggest that strategies for developing drugs to control nanotoxicity should be based on inhibition of oxidative stress initiation, activation of energy generation, and reduction in the exposure to toxic metal ions. Further study is needed to analyse the alleviation of nanotoxicity by GSH and citrate using multi-omics in order to elucidate the detailed molecular mechanism(s) of their actions.

The toxicities of SiO_2_ NPs, Ag NPs, Au NPs, CdSe QDs, PSs, TiO_2_ NPs, UPM, and MWCNTs, which are produced from widespread minerals and used in daily life, were assessed at doses of 0.01 and 0.1 μg/μl. In addition, the toxicity alleviation effects of GSH and citrate were observed in microglia for comparison with MNPs@SiO_2_(RITC)-induced toxicity at 0.01 or 0.1 μg/μl doses, and investigation of versatile alleviation effects of GSH and citrate. Morphological activation of microglia was observed after a 12 h treatment with 0.01 and 0.1 μg/μl of 50 nm SiO_2_ NPs, Ag NPs, Au NPs, and CdSe QDs. Cell death was observed in microglia treated for 12 h with 0.01 or 0.1 μg/μl doses of 2 μm and 100 nm PSs, TiO_2_ NPs, 30 nm SiO_2_ NPs, UPM, and MWCNTs, in a dose dependent manner. In a 24 h NP treatment with GSH and citrate, nanomaterial-induced cell death was attenuated, although the attenuation was deduced from omics analysis from MNPs@SiO_2_(RITC) treated cells. With respect to nanomaterial stability in cell culture medium, Ag NPs, 2 μm PSs, UPM, 30 nm SiO_2_ NPs, and TiO_2_ NPs were observed to aggregate and sediment in cell culture medium after incubation for 24 h. Chemical dosimetry in vitro is important for assessment of nanomaterial toxicity [99, 100], and further physicochemical studies, including stability, hydrodynamic size, and agglomeration state, are required for proper dose–response assessment of each nanomaterial in in vitro studies.

In the in vivo mouse model, we found that administration of MNPs@SiO_2_(RITC) induces microglia activation in the brain, whereas co-administration of GSH and citric acid reduced this activation, consistently with our in vitro findings. The levels of microglial activation events varied among brain regions (the cortex, striatum, cerebellum, hippocampus, and thalamus), according to differences in BBB permeability in each region and accumulation of MNPs@SiO_2_(RITC) and drugs [30]. Although GSH and citric acid are physiologically present in the human body, their administration efficiently reduced MNPs@SiO_2_(RITC)-induced toxicity in the brain across the BBB. Additionally, the biodistribution and toxicological parameters of MNPs@SiO_2_(RITC) were evaluated at 25, 50, and 100 mg/kg doses, and this study revealed no significant adverse effects, such as growth, behavioural changes, biochemical changes in serum, or histopathologic findings even at the 100 mg/kg dose [26]. Further studies are needed to evaluate the dose and temporal effects of NPs, GSH, citric acid, and other drugs in other organs, beyond the brain assessments performed in the present study.

MNPs@SiO_2_(RITC) accumulate in the liver, lungs, uterus, kidneys, testes, heart, spleen, and brain [26], after intraperitoneal administration in mice. MNPs@SiO_2_(RITC) penetrated the BBB and accumulated in the brain with time, as the intensity of its fluorescence increased in a time-dependent manner. Route of administration is an important factor for the biodistribution of substances in vivo [101, 102]. In the case of NPs, they can be administrated through various routes for medical purposes including oral, pulmonary, transdermal, and intravenous [103], which results in their distribution and accumulation in organs through the blood and lymphatic circulatory systems [104, 105]. Brain accumulation of NPs is determined by the penetration of the BBB, which comprises specialized structures with a basal lamina, microvessel endothelial cells, pericytes, and astrocytes [30–32, 106]. Paracellular and transcytosis pathways are the two major paths for BBB penetration [106]. The paracellular pathway is induced by disruption of tight junctions and osmotic pressure with local permeability using additional support, such as ultrasound/microbubbles, but this additional support can be a contributing factor for loss of BBB function [107]. In turn, the transcytosis pathway can be classified as adsorptive, mediated by physicochemical interactions with cells, or receptor-mediated by binding to specific receptors [108]. In general, transcytosis is initiated by clathrin- and caveolin-mediated endocytosis and escape from the endocytosis vesicle [106]. In addition, the efficiency of endocytosis is determined by surface properties and NPs size [109]. Thus, the amount of total administrated MNPs@SiO_2_(RITC) localized to the brain varies based on route, which should be considered during the usage of nanomaterials for clinical purposes.

As mentioned earlier, MNPs@SiO_2_(RITC) is assumed to remain intact in complex biological fluid and the effects of MNPs@SiO_2_(RITC) are suggested to be exerted by the silica shell. BBB penetration and brain localization of silica NPs have been analyzed using inductively coupled plasma (ICP) techniques, TEM, and X-ray fluorescence (XRF) measurement in previous studies [110, 111]. Although we analyzed the fluorescence of MNPs@SiO_2_(RITC) to detect NP localization in the brain, there is a low possibility for false-positive detection (detached RITC). In addition, and theoretically, mice weighing 25 g have ~ 2 ml of total blood [112], and brain localization of administered NPs is ~ 1% of the initial dose in biodistribution studies [110, 111]. Thus, 100 mg/kg (2.5 mg/mouse_25 g_) of administered MNPs@SiO_2_(RITC) may be localized at a concentration of ~ 0.0125 µg/µl in the brain. Moreover, in this study, fluorescence intensity of neuronal cells in the brain was similar between brain slices of mice receiving 100 mg/kg and microglia treated with ~ 0.01–0.1 µg/µl NPs in vitro. Moreover, Thus, to test whether the concentrations of MNPs@SiO_2_(RITC) in microglial cells in culture are related to those present in brain microglia, there is a pressing need for further clarification about the distribution of NPs in the brain using high-resolution confocal microscopy, X-ray absorption near edge structure spectroscopy, and inductively coupled plasma mass spectrometry for medical diagnostics and therapeutic applications.

Kim et al. [26] reported that serum biochemical changes, including glucose, cholesterol, creatinine, and the concentration ratio of aspartate transaminase and alanine transaminase were not detected in mice treated with 25, 50, and 100 mg/kg of MNPs@SiO_2_(RITC). Moreover, abnormal body weight change and behaviors were also not detected in a previous study [26]. Due to subtle toxicological phenotypes for MNPs@SiO_2_(RITC) exposure, the nanotoxicity was not detected using conventional methods in in vitro and in vivo [14, 15, 75]; however, the toxicity of MNPs@SiO_2_(RITC) has been analyzed using integrated omics and mechanobiology [13, 15, 17, 20, 25, 51, 52]. In this study, we analyzed changes in the MNPs@SiO_2_(RITC)-exposure related biological functions using triple omics integration in in vitro studies, and we extrapolated the effect of MNPs@SiO_2_(RITC) exposure on an in vivo mouse model. Although intravenous injection is a more proper route for administering contrast agents to organs for rapid delivery, when considering the onset of action, than is intraperitoneal injection [113], the in vivo experiment was performed using the same conditions, including mice species, the highest MNPs@SiO_2_(RITC) dose, and injection route, of the Kim et al. study. No abnormal body weight change nor behaviors were detected. However, in this study, the extrapolated biological function, microglial activation, was detected in MNPs@SiO_2_(RITC) treated mice brain. Thus, our results suggest that an in vitro investigation is highly recommended to precede a subtle toxicological assessment in in vivo.

MNPs@SiO_2_(RITC) were preferentially taken up by microglia than neuronal cells in in vitro and in vivo. Previous studies also reported higher uptake efficiencies by microglia over that of other neuroglia and neurons [33, 114], consistent with the results of our study. In addition to utilizing internalization mechanisms such as clathrin- and caveolin-dependent endocytosis and pinocytosis [115–117], microglia have more highly developed phagocytosis than neurons, with higher expression of phagocytosis related genes, including the triggering receptor expressed on myeloid cells 2 (*TREM2*), platelet glycoprotein 4, fatty acid translocase (*FAT*, also known as CD36), CCAAT enhancer-binding protein alpha (*C/EBPα*), leucine-rich repeat kinase 2 (*LRRK2*), lysosomal associated membrane protein 3 (*LAMP3*, also known as CD 208), and lysosomal associated membrane protein 4 (*LAMP4*, also known as CD68) [114, 118]. This might explain the increased uptake of nanoparticles by microglia compared to neurons. Additionally, we found that the uptake of MNPs@SiO_2_(RITC) by microglia was associated with phagocytosis, whereas the exocytosis process turned out to be suppressed in the triple-omics network analysis (Additional file 1: Figs. S39 and S40 and Table S11) and in the prediction of the triple omics network in MNPs@SiO_2_(RITC)-treated BV2 cells (Additional file 1: Figs. S41 and S42). Particle transport into cells is physically mediated by diffusion (size fraction 25–50 nm), sedimentation (size fraction 250–500 nm), and agglomeration in a particle density (concentration)-dependent manner [119]. In addition, cells uptake NPs through clathrin-mediated endocytosis (endocytic vesicle size: ~ 100 nm), caveolae-mediated endocytosis (endocytic vesicle size: ~ 60 nm), clathrin–caveolin-independent endocytosis (endocytic vesicle size: ~ 100 nm), micropinocytosis (endocytic vesicle size: > 200 nm), and phagocytosis (endocytic vesicle size: > 200 nm), according to size, shape, and physicochemical properties of the NPs [120, 121]. In our results, diameters of vesicles containing > 30 MNPs@SiO_2_(RITC) agglomerates in the MNPs@SiO_2_(RITC)-treated microglial cells were greater than 500 nm, implying that the endocytic process was dominantly micropinocytosis or phagocytosis [122, 123]. Moreover, morphological activation is also called ‘phagocytic activation’ and indicates that the phagocytic pathway is dominant in this state [124]. On the other hand, the phagocytic pathway is not highly developed in other cell types [122]. Suppression of exocytosis may be fundamentally related to nanotoxicity due to the resistance of NPs to biodegradation. Therefore, high uptake efficiency in microglia might be mediated by phagocytosis, which is highly developed in the macrophage lineage [114, 118], and activation of exocytosis in the cells taking up NPs may be one of the strategies for reducing nanotoxicity.

## Conclusions

This study suggests that exposure to 0.1 µg/µl dose of MNPs@SiO_2_(RITC) can cause ROS production, inflammatory response, microglial activation, and a glucose metabolism disorder according to integral analysis using computational prediction by means of triple omics, as compared to 0.01 µg/µl dose of MNPs@SiO_2_(RITC) in microglia. These findings highlight the importance of using appropriate doses of NPs in terms of possible adverse effects and may help to develop new drugs reducing nanotoxicity.

## Supplementary Information

**Additional file 1:** Supplementary Materials.

## Data Availability

The data supporting the findings of this study are available from the corresponding author, upon reasonable request. Transcriptome sequencing and quantification data are available in the GEO database under the following accession number: GSE154250. Proteome and quantification data are available in PRIDE with following accession number: PXD020225.

## Abbreviations

AAs: Amino acids
ANOVA: One-way analysis of variance
BBB: Blood–brain barrier
C/EBPα: CCAAT enhancer-binding protein alpha
CoFe_2_O_3_: Cobalt ferrite
ECL: Enhanced chemiluminescence
EOC: Ethoxycarbonyl
FAs: Fatty acids
FAT: Platelet glycoprotein 4, fatty acid translocase
G6PDH: Glucose-6-phosphate dehydrogenase
GC–MS/MS: Gas chromatography with tandem mass spectrometry
GLUT1: Glucose transporter 1
GSH: Glutathione
HIF: Hypoxia-inducible factor
HSD: Tukey’s honestly significant difference
ICP: Inductively coupled plasma
IPA: Ingenuity pathway analysis
*k*-NN: *k*-Nearest neighbor
LAMP3: Lysosomal associated membrane protein 3
LAMP4: Lysosomal associated membrane protein 4
LC–MS/MS: Liquid chromatography with tandem mass spectrometry
LPS: Lipopolysaccharide
LRRK2: Leucine-rich repeat kinase 2
MEM: Minimum essential medium
MNPs: Magnetic nanoparticles
MNPs@SiO_2_(RITC): Silica-coated-magnetic nanoparticles containing the rhodamine B isothiocyanate dye
MO: Methoxime
MWCNTs: Multi-walled carbon nanotubes
NAC: *N*-Acetyl cysteine
NADP^+^: Nicotinamide adenine dinucleotide phosphate
Nanotoxicity: Nanomaterial-induced toxicity
NeuN: Neuronal nuclei
NIST: National Institute of Standards and Technology
NMMA: _L_-NG-monomethyl-_L_-arginine
NPs: Nanoparticles
OAs: Organic acids
OD: Outer diameter
PCA: Principal component analysis
PSs: Polystyrene particles
QDs: Quantum dots
qPCR: Quantitative PCR
ROS: Reactive oxygen species
TBDMS: *tert*-Butyldimethylsilyl
TCA: Tricarboxylic acid
TEM: Transmission electron microscopy
TH: Tyrosine hydroxylase
TREM2: Triggering receptor expressed on myeloid cells 2
UPM: Urban particulate matter
XRF: X-ray fluorescence
