# Supplementary material for: Silica-coated magnetic-nanoparticle-induced cytotoxicity is reduced in microglia by glutathione and citrate identified using integrated omics
Source: Part Fibre Toxicol. 2021 Nov 25;18:42. doi: 10.1186/s12989-021-00433-y (PMC8614058; doi:10.1186/s12989-021-00433-y)
Supplement: Supplementary file 1 — Additional file 1: Supplementary Materials. [file 12989_2021_433_MOESM1_ESM.docx]

**Additional File 1**

**Silica-coated magnetic-nanoparticle-induced cytotoxicity is reduced in microglia by glutathione and citrate identified using integrated omics**

Tae Hwan Shin^1^, Balachandran Manavalan^1^, Da Yeon Lee^1^, Shaherin Basith^1^, Chan Seo^2^, Man Jeong Paik^2^, Sang-Wook Kim^3^, Haewoon Seo^3^, Ju Yeon Lee^4^, Jin Young Kim^4^, A Young Kim^1^, Jee Min Chung^1^, Eun Joo Baik^1^, Seong Ho Kang^5^, Dong-Kug Choi^6^, Yup Kang^1^, M. Maral Mouradian^7^, Gwang Lee^1,3,*^

*^1^Department of Physiology, Ajou University School of Medicine, 206 World cup-ro, 16499 Suwon, Republic of Korea*

*^2^College of Pharmacy, Sunchon National University, 255 Jungang-ro, 57922 Suncheon, Republic of Korea*

*^3^Department of Molecular Science and Technology, Ajou University, 206 World cup-ro, 16499 Suwon, Republic of Korea*

*^4^Research Center of Bioconvergence Analysis, Korea Basic Science Institute, 162 Yeongudanji-ro, 28119 Cheongju, Republic of Korea*

*^5^Department of Chemistry, Graduate School, Kyung Hee University, Yongin-si, Gyeonggi-do 17104, Republic of Korea and Department of Applied Chemistry and Institute of Natural Sciences, Kyung Hee University, Yongin-si, Gyeonggi-do 17104, Republic of Korea*

*^6^Department of Biotechnology, College of Biomedical and Health Science, Konkuk University, 268 Chungwondaero, 27478 Chungju, Republic of Korea*

*^7^RWJMS Institute for Neurological Therapeutics, Rutgers Biomedical and Health Sciences, and Department of Neurology, Robert Wood Johnson Medical School, Rutgers University, Piscataway, 08854 New Jersey, USA*

^*^Corresponding author:

Gwang Lee, Professor

Department of Physiology, Ajou University School of Medicine, World cup-ro,

Yeongtong-gu, Suwon 16499, Republic of Korea

Tel.: +82-31-219-4554

Fax: +82-31-219-5049

E-mail: glee@ajou.ac.kr

**Supplementary Methods**

**Evaluation of intracellular ROS levels**

Intracellular ROS was analyzed using 2′,7′-Dichlorodihydrofluorescein diacetate (DCFH-DA, Cell Biolabs, CA, USA). Briefly, HEK293 cells were treated with MNPs@SiO_2_(RITC) for 12 h. Ten µM DCFH-DA was added and incubated at 37°C/5% CO_2_ for 1 h. After twice wash with PBS, fluorescence images of oxidized form of DCFH-DA (DCF) were taken with an AxioVert 200M fluorescence microscopy (Zeiss, Jena, Germany) at the 3D immune system imaging core facility of Ajou University.

**Evaluation of morphological activation of microglia**

To evaluate MNPs@SiO_2_(RITC)-induced microglia activation, BV2 and primary rat microglia cells were treated with 0.01 and 0.1 µg/µl of MNPs@SiO_2_(RITC) or 0.01 and 0.1 ng/µl of LPS as activation control for 12 h. Images were taken with an AxioVert 200M fluorescence microscopy (Zeiss, Jena, Germany) at the Three-Dimensional Immune System Imaging Core Facility of Ajou University. Activated microglia, which had unramified morphology, were counted and compared to untreated control cells.

**Flow cytometry**

BV2 and primary rat microglia cells (10^6^ cells) were treated with 0.01 and 0.1 µg/µl of MNPs@SiO_2_(RITC) or 0.01 and 0.1 ng/µl of LPS as an activation control for 12 h. Then, cells were detached from plate and fixed in Cytofix buffer (BD, San Jose, CA, USA) for 30 min in room temperature. Cells were incubated for 1 h in room temperature with anti-OX42 mouse monoclonal antibody (1:100, Santa Cruz Biotechnology) for primary rat microglia purity, and with anti-OX6 mouse polyclonal antibody (1:100, Abcam) for microglial activation diluted in PBS. FITC-conjugated anti-mouse goat antibody (1:100, Vector Laboratories) was used for fluorescence label. After two washes with PBS, cells were analyzed using a flow cytometer (BD FACS Aria II™) at the 3D immune system imaging core facility of Ajou University.

**Total RNA isolation and cDNA library preparation for transcriptome sequencing (RNA-seq)**

BV2 cells were treated with 0.01 and 0.1 µg/µl of MNPs@SiO_2_(RITC) for 12 h, lysed, and total RNA was isolated with TruSeq Stranded Total RNA Library Prep Kit (Illumina, CA). RNA quality was analyzed by rRNA band integrity on an Agilent RNA 6000 Nano kit (Agilent Technologies, CA). cDNA library was constructed from 1 µg of total RNA. To enrich poly A tailed mRNA, magnetic beads conjugated with oligo (dT) were used. Isolated mRNAs were disintegrated into short fragments, and double-stranded cDNAs were generated. The cDNAs were modified with end-repair, poly A addition, and connected with sequencing adapters using the TruSeq RNA sample prep Kit (Illumina, CA). cDNA fragments were purified with BluePippin (Sage Science, MA) and amplified by PCR. The cDNA library size and quality were determined with an Agilent High Sensitivity DNA kit (Agilent Technologies, CA). The library was sequenced using an Illumina HiSeq2500 sequencer (Illumina, CA).

**High pH reversed-phase liquid chromatography for peptide fractionation**

Six hundred milligrams of tandem mass tag (TMT) labeled sample was separated using a 4.6 mm × 250 nm, C18, 5 μm column (Shim Pack, SHIMADZU, Japan) on NexeraXR HPLC (SHIMADZU, Japan) with a 60 min gradient with five to ninety-five% mobile phases B at a flow rate of 0.5 ml/min. The mobile phases A (10 mM ammonium formate in water) and mobile phases B (10 mM ammonium formate in 90% acetonitrile) were adjusted to pH 10 with NH_4_OH. FRC-10A fraction collector (SHIMADZU, Japan) was used for collecting fractions for 32 min with 1 min intervals. The fractions were merged as 10 fractions and desalted.

**Protein identification and quantification**

MS/MS spectra were identified using Uniprot mouse database (Dec/11^st^/2017 released). Calculation of false discovery rate (FDR) was conducted with database of reversed sequences of all proteins. Identification of the peptide were performed using ProLucid [1]. Static modifications were used with TMT modification (+ 229.1629) at the N-terminus and lysine residue and variable modification used with oxidation at methionine. Reporter ions were obtained from small windows (± 20 ppm) and the data were arranged with peptides assignments and filtered a false positive rate less than 0.01 using the DTASelect (The Scripps Research Institute, USA) [2]. Quantification using Census (Integrated Proteomics, USA) was conducted. The intensity of a reporter ion channel was determined as the average of the ion intensity from all peptides from the protein [3].

**Differentially expressed gene analysis using RNA-seq data**

Reads were identified with the mouse reference genome using the aligner Tophat [4]. Gene expression level was determined with Cufflinks v2.1.1 [4], using the gene annotation database of Ensembl release 77. Multi-read-correction and fragbias- correct options were used for elevation of accuracy. DEG were identified using Cuffdiff tool with default set with a significance of *p* value < 0.05.

**Sample preparation for metabolic profiling**

Thirteen FAs, 20 AAs, and 14 OAs were analyzed with EOC/MO/TBDMS derivatives on GC-MS/MS [5, 6]. Cells were lysed by freeze-thawing, and pentadecanoic acid (internal standard for FAs), norvaline (internal standard for AAs), and dimethoxybenzoic acid (internal standard for OAs) was mixed to each sample. Each sample was reacted with methoxyamine hydrochloride at 60°C for 60 min with 5.0 M sodium hydroxide in dichloromethane containing ECF and MO derivative. The samples, adjusted to a pH ≥ 12, were acidified (pH ≤ 2.0 with 10% sulphuric acid). Sodium chloride were added at saturation in sample and diethyl ether and ethyl acetate were used for extraction. The samples were evaporated and reacted with triethylamine, toluene, and MTBSTFA at 60°C for 30 min.

**Gene Ontology analysis and pathway analysis**

Mouse Genome Informatics (MGI) [7] was used as Gene Ontology (GO) database. Biological pathways and functions were analyzed using the IPA bioinformatics software (Qiagen, Valencia, CA, USA). A 1.5-fold change for gene and protein expression and a 1.2-fold change for metabolite levels were used as a cut-off value for significant changes.

**RNA isolation and** **quantitative real-time PCR**

BV2 cells were treated with 0.01 or 0.1 µg/µl MNPs@SiO_2_(RITC) for 12 h and were lysed with RNAzol B solution. chloroform was added and incubated for 5 min on ice. Isopropyl alcohol was added to precipitate total RNA. The precipitated RNA was washed in 70% ethanol and the RNA was dissolved in RNase-free water. A cDNA library was generated using the iScript Advanced cDNA Synthesis Kit (BioRad, Hercules, CA, USA). The reaction conditions were as follows: 46°C for 20 min followed by 95°C for 1 min.

The expression of transcriptomic network related genes was detected by quantitative real-time PCR (qPCR) using the SsoAdvanced™ Universal SYBR® Green real-time PCR kit (BioRad, Hercules, CA, USA) with gene-specific primer pairs (Table S12) on a Rotor Gene-Q system (Qiagen, Valencia, CA, USA). Expression levels were determined with melting curve using Rotor-Gene 1.7 software (Qiagen, Valencia, CA, USA). PCR reactions were prepared as independent triplicate samples. The relative quantification of target gene expression was calculated by the 2^−ΔΔCt^ method.

**Cell viability assay**

Microglia were seeded at 2 × 10^4^ cells/well in a 96-well plate. In the case of 12 h assay, cells were treated with 0.01, 0.1, and 1.0 µg/µl MNPs@SiO_2_(RITC) for 12 h. In the case of 24 h assay, 0.1 µg/µl nanoparticles and each drug or combination of 0.25 mM GSH and 1 mM citric acid were added for 24 h. Cell viability was determined by CellTilter 96® Aqueous One Solution Cell Proliferation Assay (Promega Corporation, Madison, WI, USA), according to the manufacturer’s instructions.

**Measurement of Adenosine Triphosphate (ATP) concentration**

ATP concentration of MNPs@SiO_2_(RITC) treated microglia was measured using an ATP assay system (Promega, USA) according to the manufacturer's protocol. Briefly, microglia were seeded 2 × 10^4^ in 96 well plate and treated with MNPs@SiO_2_(RITC) for 24 h. Microglia were trypsinized and counted. Luciferin reagent with cell lysis agent was mixed 1 × 10^4^ cells. The mixtures were resuspended and then cells were split in 384-well white plates. After 20 min of incubation at room temperature, luminescence of each well was analyzed using Synergy 2 luminometer (BioTek, Winooski, VT, USA) and the images were taken using a ChemiDoc™ Touch Gel Imaging System (Bio-Rad, Hercules, CA, USA).

**Table S1**. Ingenuity Pathway Analysis-based profiles of transcriptome of BV2 cells treated with MNPs*@*SiO_2_(RITC)

| Entrez gene name | Symbol | Ensembl ID | Location | Signal fold change^a^ | |
| --- | --- | --- | --- | --- | --- |
|  |  |  |  | 0.01 μg/μl | 0.1 μg/μl |
| ATP binding cassette subfamily D member 2 | ABCD2 | ENSMUSG00000055782 | Cytoplasm | -1.20 | -2.25 |
| angiotensin I converting enzyme | ACE | ENSMUSG00000020681 | Plasma Membrane | 3.17 | 1.67 |
| adenosine A2a receptor | ADORA2A | ENSMUSG00000020178 | Plasma Membrane | -2.00 | -3.00 |
| advanced glycosylation end-product specific receptor | AGER | ENSMUSG00000015452 | Plasma Membrane | 1.41 | 1.68 |
| aryl hydrocarbon receptor | AHR | ENSMUSG00000019256 | Nucleus | 1.56 | 1.67 |
| allograft inflammatory factor 1 | AIF1 | ENSMUSG00000024397 | Nucleus | -1.32 | -1.69 |
| arachidonate 15-lipoxygenase | ALOX15 | ENSMUSG00000018924 | Cytoplasm | 1.22 | 2.39 |
| apelin | APLN | ENSMUSG00000037010 | Extracellular Space | -3.50 | -2.33 |
| aquaporin 9 | AQP9 | ENSMUSG00000032204 | Plasma Membrane | -1.07 | -1.80 |
| BCL2 related protein A1 | BCL2A1 | ENSMUSG00000099974 | Cytoplasm | 1.07 | -1.58 |
| bradykinin receptor B1 | BDKRB1 | ENSMUSG00000041347 | Plasma Membrane | 1.40 | 1.55 |
| calcium/calmodulin dependent protein kinase ID | CAMK1D | ENSMUSG00000039145 | Cytoplasm | -1.20 | -2.07 |
| C-C motif chemokine ligand 2 | CCL2 | ENSMUSG00000035352 | Extracellular Space | 1.38 | 2.40 |
| C-C motif chemokine receptor 1 | CCR1 | ENSMUSG00000025804 | Plasma Membrane | -1.09 | 1.57 |
| C-C motif chemokine receptor 5 (gene/pseudogene) | CCR5 | ENSMUSG00000079227 | Plasma Membrane | 1.27 | 2.15 |
| CD33 antigen | CD33 | ENSMUSG00000004609 | Plasma Membrane | -1.09 | -2.18 |
| CD4 molecule | CD4 | ENSMUSG00000023274 | Plasma Membrane | 1.25 | 3.94 |
| CD72 molecule | CD72 | ENSMUSG00000028459 | Plasma Membrane | 1.37 | -1.66 |
| CCAAT enhancer binding protein delta | CEBPD | ENSMUSG00000071637 | Nucleus | -1.02 | -1.58 |
| complement factor H | CFH | ENSMUSG00000026365 | Extracellular Space | 1.04 | -1.60 |
| class II major histocompatibility complex transactivator | CIITA | ENSMUSG00000022504 | Nucleus | 1.00 | 2.00 |
| cardiotrophin like cytokine factor 1 | CLCF1 | ENSMUSG00000040663 | Extracellular Space | 1.07 | 1.79 |
| C-type lectin domain family 12 member A | CLEC12A | ENSMUSG00000053063 | Plasma Membrane | -1.00 | -1.70 |
| C-type lectin domain family 4 member A | CLEC4A | ENSMUSG00000030148 | Plasma Membrane | 1.03 | -1.55 |
| C-type lectin domain containing 7A | CLEC7A | ENSMUSG00000079293 | Plasma Membrane | 1.03 | -1.50 |
| colony stimulating factor 3 | CSF3 | ENSMUSG00000038067 | Extracellular Space | 1.20 | 2.48 |
| colony stimulating factor 3 receptor | CSF3R | ENSMUSG00000028859 | Plasma Membrane | 1.09 | -1.55 |
| CXADR, Ig-like cell adhesion molecule | CXADR | ENSMUSG00000022865 | Plasma Membrane | 1.59 | 2.26 |
| C-X-C motif chemokine ligand 3 | CXCL3 | ENSMUSG00000058427 | Extracellular Space | 1.35 | 2.67 |
| C-X-C motif chemokine receptor 1 | CXCR1 | ENSMUSG00000048480 | Plasma Membrane | 1.50 | -2.25 |
| cytochrome b-245 beta chain | CYBB | ENSMUSG00000015340 | Cytoplasm | -1.12 | -1.70 |
| cytochrome P450, family 4, subfamily f, polypeptide 16 | Cyp4f16/Cyp4f37 | ENSMUSG00000062464 | Cytoplasm | 1.00 | 1.83 |
| death associated protein kinase 2 | DAPK2 | ENSMUSG00000032380 | Cytoplasm | 1.00 | 1.75 |
| elastase, neutrophil expressed | ELANE | ENSMUSG00000020125 | Extracellular Space | 1.00 | 1.55 |
| endoglin | ENG | ENSMUSG00000026814 | Plasma Membrane | 1.68 | 1.55 |
| EPH receptor A2 | EPHA2 | ENSMUSG00000006445 | Plasma Membrane | -1.27 | -1.50 |
| EPH receptor B2 | EPHB2 | ENSMUSG00000028664 | Plasma Membrane | 2.67 | 3.83 |
| Fas cell surface death receptor | FAS | ENSMUSG00000024778 | Plasma Membrane | -1.08 | -1.56 |
| FGR proto-oncogene, Src family tyrosine kinase | FGR | ENSMUSG00000028874 | Nucleus | -1.63 | -2.04 |
| forkhead box P3 | FOXP3 | ENSMUSG00000039521 | Nucleus | -1.43 | -1.61 |
| formyl peptide receptor 1 | FPR1 | ENSMUSG00000045551 | Plasma Membrane | -1.38 | -4.06 |
| formyl peptide receptor 2 | FPR2 | ENSMUSG00000052270 | Plasma Membrane | -1.26 | -1.92 |
| GATA binding protein 3 | GATA3 | ENSMUSG00000015619 | Nucleus | 2.99 | 3.51 |
| gap junction protein alpha 1 | GJA1 | ENSMUSG00000050953 | Plasma Membrane | 1.17 | -1.50 |
| gap junction protein gamma 2 | GJC2 | ENSMUSG00000043448 | Plasma Membrane | 2.00 | 2.00 |
| gonadotropin releasing hormone 1 | GNRH1 | ENSMUSG00000015812 | Extracellular Space | 1.04 | 2.79 |
| G protein-coupled receptor 183 | GPR183 | ENSMUSG00000051212 | Plasma Membrane | 1.05 | -1.67 |
| G protein-coupled receptor 68 | GPR68 | ENSMUSG00000047415 | Plasma Membrane | 1.18 | 1.94 |
| histone deacetylase 9 | HDAC9 | ENSMUSG00000004698 | Nucleus | -1.14 | -1.80 |
| major histocompatibility complex, class I, A | HLA-A | ENSMUSG00000067235 | Plasma Membrane | 1.00 | -3.50 |
| hepatocyte nuclear factor 4 alpha | HNF4A | ENSMUSG00000017950 | Nucleus | 1.67 | 2.00 |
| haptoglobin | HP | ENSMUSG00000031722 | Extracellular Space | -1.18 | -2.69 |
| heat shock protein family B (small) member 1 | HSPB1 | ENSMUSG00000004951 | Cytoplasm | 1.00 | -1.88 |
| immunoglobulin heavy constant mu | IGHM | ENSMUSG00000076617 | Plasma Membrane | 1.13 | -2.91 |
| interleukin 10 | IL10 | ENSMUSG00000016529 | Extracellular Space | -1.28 | -2.11 |
| interleukin 10 receptor subunit alpha | IL10RA | ENSMUSG00000032089 | Plasma Membrane | -1.09 | -1.67 |
| interleukin 17 receptor B | IL17RB | ENSMUSG00000015966 | Plasma Membrane | -1.15 | 2.07 |
| interleukin 1 receptor type 1 | IL1R1 | ENSMUSG00000026072 | Plasma Membrane | 1.23 | -1.73 |
| interleukin 1 receptor antagonist | IL1RN | ENSMUSG00000026981 | Extracellular Space | 1.14 | 1.64 |
| interleukin 23 subunit alpha | IL23A | ENSMUSG00000025383 | Extracellular Space | -1.07 | 2.91 |
| interleukin 34 | IL34 | ENSMUSG00000031750 | Extracellular Space | -1.46 | -2.02 |
| interferon regulatory factor 6 | IRF6 | ENSMUSG00000026638 | Nucleus | -1.50 | -1.71 |
| integrin subunit alpha L | ITGAL | ENSMUSG00000030830 | Plasma Membrane | -1.28 | -2.31 |
| integrin subunit beta 3 | ITGB3 | ENSMUSG00000020689 | Plasma Membrane | 2.00 | 2.33 |
| potassium voltage-gated channel subfamily A member 3 | KCNA3 | ENSMUSG00000047959 | Plasma Membrane | -1.01 | -1.55 |
| potassium voltage-gated channel subfamily E regulatory subunit 3 | KCNE3 | ENSMUSG00000035165 | Plasma Membrane | 3.41 | 6.95 |
| lipocalin 2 | LCN2 | ENSMUSG00000026822 | Extracellular Space | -1.11 | -1.70 |
| LIF, interleukin 6 family cytokine | LIF | ENSMUSG00000034394 | Extracellular Space | 8.83 | 2.50 |
| leukocyte immunoglobulin like receptor B3 | LILRB3 | ENSMUSG00000074417 | Plasma Membrane | 1.00 | -2.00 |
| lymphotoxin alpha | LTA | ENSMUSG00000024402 | Extracellular Space | 1.50 | 1.76 |
| leukotriene B4 receptor | LTB4R | ENSMUSG00000046908 | Plasma Membrane | 1.50 | -1.90 |
| latent transforming growth factor beta binding protein 1 | LTBP1 | ENSMUSG00000001870 | Extracellular Space | -1.21 | -2.27 |
| microtubule associated protein tau | MAPT | ENSMUSG00000018411 | Plasma Membrane | 11.29 | 5.26 |
| macrophage receptor with collagenous structure | MARCO | ENSMUSG00000026390 | Plasma Membrane | -1.17 | -1.93 |
| MAS1 proto-oncogene, G protein-coupled receptor | MAS1 | ENSMUSG00000068037 | Plasma Membrane | -1.30 | -1.68 |
| midkine | MDK | ENSMUSG00000027239 | Extracellular Space | -1.38 | -1.98 |
| major facilitator superfamily domain containing 2A | MFSD2A | ENSMUSG00000028655 | Plasma Membrane | -1.27 | 1.79 |
| matrix metallopeptidase 14 | MMP14 | ENSMUSG00000000957 | Extracellular Space | -1.03 | -1.71 |
| matrix metallopeptidase 28 | MMP28 | ENSMUSG00000020682 | Extracellular Space | -1.69 | -2.25 |
| MOK protein kinase | MOK | ENSMUSG00000056458 | Plasma Membrane | 1.02 | 1.80 |
| methionine sulfoxide reductase A | MSRA | ENSMUSG00000054733 | Cytoplasm | -1.14 | -1.54 |
| MYC proto-oncogene, bHLH transcription factor | MYC | ENSMUSG00000022346 | Nucleus | 1.15 | -1.51 |
| NFKB inhibitor zeta | NFKBIZ | ENSMUSG00000035356 | Nucleus | 1.04 | -1.54 |
| nodal growth differentiation factor | NODAL | ENSMUSG00000037171 | Extracellular Space | 2.41 | 1.88 |
| nitric oxide synthase 1 | NOS1 | ENSMUSG00000029361 | Cytoplasm | 1.42 | 1.74 |
| notch 1 | NOTCH1 | ENSMUSG00000026923 | Plasma Membrane | 1.02 | -1.58 |
| NADPH oxidase 1 | NOX1 | ENSMUSG00000031257 | Cytoplasm | 1.38 | -2.06 |
| 5'-nucleotidase ecto | NT5E | ENSMUSG00000032420 | Plasma Membrane | 1.50 | 1.50 |
| nuclear protein 1, transcriptional regulator | NUPR1 | ENSMUSG00000030717 | Nucleus | 1.00 | -1.81 |
| oxidized low density lipoprotein receptor 1 | OLR1 | ENSMUSG00000030162 | Plasma Membrane | -1.43 | 2.27 |
| phosphodiesterase 4D, cAMP specific | PDE4D | ENSMUSG00000021699 | Cytoplasm | 1.46 | 2.05 |
| pyruvate dehydrogenase kinase 2 | PDK2 | ENSMUSG00000038967 | Cytoplasm | 1.33 | 1.58 |
| platelet and endothelial cell adhesion molecule 1 | PECAM1 | ENSMUSG00000020717 | Plasma Membrane | -1.91 | -1.58 |
| peroxisome proliferator activated receptor gamma | PPARG | ENSMUSG00000000440 | Nucleus | 1.09 | 1.82 |
| PR/SET domain 1 | PRDM1 | ENSMUSG00000038151 | Nucleus | 1.21 | -1.51 |
| protein kinase C beta | PRKCB | ENSMUSG00000052889 | Cytoplasm | -4.43 | -3.88 |
| protein kinase C theta | PRKCQ | ENSMUSG00000026778 | Cytoplasm | 1.00 | 2.00 |
| proteinase 3 | PRTN3 | ENSMUSG00000057729 | Extracellular Space | -1.64 | 1.61 |
| prostaglandin E receptor 2 | PTGER2 | ENSMUSG00000037759 | Plasma Membrane | 1.13 | -2.25 |
| prostaglandin I2 synthase | PTGIS | ENSMUSG00000017969 | Cytoplasm | -1.53 | -1.92 |
| PYD and CARD domain containing | PYCARD | ENSMUSG00000030793 | Cytoplasm | 1.29 | -1.70 |
| PZP, alpha-2-macroglobulin like | PZP | ENSMUSG00000030359 | Extracellular Space | 1.00 | -4.25 |
| selectin L | SELL | ENSMUSG00000026581 | Plasma Membrane | -1.13 | 1.59 |
| selectin P | SELP | ENSMUSG00000026580 | Plasma Membrane | 3.11 | 1.89 |
| sialic acid binding Ig like lectin 9 | SIGLEC9 | ENSMUSG00000030474 | Plasma Membrane | -1.11 | -1.94 |
| signaling lymphocytic activation molecule family member 1 | SLAMF1 | ENSMUSG00000015316 | Plasma Membrane | 1.17 | 2.00 |
| solute carrier family 1 member 2 | SLC1A2 | ENSMUSG00000005089 | Plasma Membrane | -2.74 | -4.20 |
| suppressor of cytokine signaling 1 | SOCS1 | ENSMUSG00000038037 | Cytoplasm | -1.03 | 1.60 |
| SRC kinase signaling inhibitor 1 | SRCIN1 | ENSMUSG00000038453 | Cytoplasm | -1.47 | -1.57 |
| STEAP2 metalloreductase | STEAP2 | ENSMUSG00000015653 | Plasma Membrane | 1.00 | 4.00 |
| thromboxane A synthase 1 | TBXAS1 | ENSMUSG00000029925 | Plasma Membrane | -1.08 | -2.64 |
| TEK receptor tyrosine kinase | TEK | ENSMUSG00000006386 | Plasma Membrane | 2.00 | 3.00 |
| toll-like receptor 12 | TLR12 | ENSMUSG00000062545 | Other | -2.16 | -1.93 |
| toll like receptor 3 | TLR3 | ENSMUSG00000031639 | Plasma Membrane | -1.14 | -4.00 |
| toll like receptor 8 | TLR8 | ENSMUSG00000040522 | Plasma Membrane | -1.15 | -2.07 |
| TNF receptor superfamily member 14 | TNFRSF14 | ENSMUSG00000042333 | Plasma Membrane | 1.30 | -1.84 |
| TNF superfamily member 14 | TNFSF14 | ENSMUSG00000005824 | Extracellular Space | 1.12 | 1.56 |
| tubulin alpha 4a | TUBA4A | ENSMUSG00000026202 | Cytoplasm | 1.00 | -2.40 |
| tubulin beta 1 class VI | TUBB1 | ENSMUSG00000016255 | Cytoplasm | 1.26 | -1.50 |
| thioredoxin interacting protein | TXNIP | ENSMUSG00000038393 | Cytoplasm | 1.15 | 1.65 |
| unc-13 homolog D | UNC13D | ENSMUSG00000057948 | Cytoplasm | -1.83 | -1.83 |

^a^Normalized signal fold change of signal in treated groups with MNPs@SiO_2_(RITC) to corresponding signal of control group

**Table S2**. Top 20 canonical pathways constructed algorithmically by Ingenuity Pathway Analysis in transcriptome of BV2 cells treated with MNPs*@*SiO_2_(RITC)

| Canonical pathways | -log(p-value) |
| --- | --- |
| Protein Kinase A Signaling | 3.06 |
| Gustation Pathway | 3.04 |
| Granulocyte Adhesion and Diapedesis | 2.98 |
| GP6 Signaling Pathway | 2.88 |
| Altered T Cell and B Cell Signaling in Rheumatoid Arthritis | 2.87 |
| Leukocyte Extravasation Signaling | 2.75 |
| Cardiac β-adrenergic Signaling | 2.71 |
| CCR5 Signaling in Macrophages | 2.69 |
| Axonal Guidance Signaling | 2.55 |
| Hepatic Fibrosis / Hepatic Stellate Cell Activation | 2.44 |
| Eicosanoid Signaling | 2.38 |
| NAD biosynthesis II (from tryptophan) | 2.1 |
| cAMP-mediated signaling | 2.1 |
| Th1 and Th2 Activation Pathway | 2.09 |
| Th1 Pathway | 2.04 |
| Crosstalk between Dendritic Cells and Natural Killer Cells | 1.99 |
| Phagosome Formation | 1.82 |
| Complement System | 1.82 |
| Neuroprotective Role of THOP1 in Alzheimer's Disease | 1.8 |
| Notch Signaling | 1.76 |

**Table S3**. Top 20 biological functions constructed algorithmically by Ingenuity Pathway Analysis in transcriptome of BV2 cells treated with MNPs*@*SiO_2_(RITC)

| Biological functions | p-value |
| --- | --- |
| Cancer | 1.78 × 10^-22^ |
| Organismal Injury and Abnormalities | 1.78 × 10^-22^ |
| Dermatological Diseases and Conditions | 9.55 × 10^-21^ |
| Endocrine System Disorders | 4.38 × 10^-17^ |
| Gastrointestinal Disease | 1.82 × 10^-14^ |
| Hematological System Development and Function | 4.77 × 10^-13^ |
| Tissue Morphology | 4.77 × 10^-13^ |
| Cellular Development | 1.83 × 10^-12^ |
| Cellular Growth and Proliferation | 1.83× 10^-12^ |
| Lymphoid Tissue Structure and Development | 1.83 × 10^-12^ |
| Reproductive System Disease | 1.92 × 10^-11^ |
| Cellular Movement | 4.18 × 10^-11^ |
| Inflammatory Response | 1.09 × 10^-10^ |
| Metabolic Disease | 1.12 × 10^-10^ |
| Cellular Function and Maintenance | 1.48 × 10^-10^ |
| Connective Tissue Disorders | 3.3 × 10^-10^ |
| Inflammatory Disease | 3.3 × 10^-10^ |
| Skeletal and Muscular Disorders | 3.3 × 10^-10^ |
| Immunological Disease | 3.42 × 10^-10^ |
| Hematopoiesis | 1.69 × 10^-9^ |

**Table S4**. Ingenuity Pathway Analysis-based profiles of proteome of BV2 cells treated with MNPs*@*SiO_2_(RITC)

| Entrez gene name | Symbol | UniProt ID | Location | Signal fold change^a^ | |
| --- | --- | --- | --- | --- | --- |
|  |  |  |  | 0.01 μg/μl | 0.1 μg/μl |
| albumin | ALB | P07724 | Extracellular Space | 4.27 | 1.90 |
| apolipoprotein A1 | APOA1 | Q00623 | Extracellular Space | 9.78 | 5.11 |
| complement C5a receptor 1 | C5AR1 | P30993 | Plasma Membrane | -0.53 | -3.45 |
| CD14 molecule | CD14 | P10810 | Plasma Membrane | 2.15 | 4.95 |
| CD36 molecule | CD36 | Q08857 | Plasma Membrane | -2.44 | -3.34 |
| CD80 molecule | CD80 | Q00609 | Plasma Membrane | 2.11 | 5.43 |
| CCAAT enhancer binding protein beta | CEBPB | P28033 | Nucleus | -7.26 | 3.35 |
| chemerin chemokine-like receptor 1 | CMKLR1 | P97468 | Plasma Membrane | -0.07 | 1.98 |
| cathepsin B | CTSB | P10605 | Cytoplasm | 1.96 | 3.07 |
| coagulation factor VII | F7 | P70375 | Plasma Membrane | 3.91 | 2.31 |
| Fas cell surface death receptor | FAS | P25446 | Plasma Membrane | -3.73 | -1.76 |
| Fc fragment of IgE receptor Ig | FCER1G | P20491 | Plasma Membrane | -1.49 | -2.92 |
| Fc fragment of IgG receptor Ia | FCGR1A | P26151 | Plasma Membrane | -0.83 | -2.02 |
| ferritin heavy chain 1 | FTH1 | P09528 | Cytoplasm | -1.22 | 2.29 |
| G protein subunit alpha 13 | GNA13 | P27601 | Plasma Membrane | 1.16 | 2.33 |
| G protein-coupled receptor 183 | GPR183 | Q3U6B2 | Plasma Membrane | 1.92 | 6.76 |
| glutathione peroxidase 1 | GPX1 | P11352 | Cytoplasm | 1.05 | 2.48 |
| glutathione S-transferase mu 1 | GSTM1 | P15626 | Cytoplasm | -1.17 | -2.51 |
| glutathione S-transferase mu 5 | GSTM5 | P10649 | Cytoplasm | -1.63 | -3.24 |
| histone deacetylase 5 | HDAC5 | Q9Z2V6 | Nucleus | -0.02 | -2.11 |
| heat shock protein family A (Hsp70) member 5 | HSPA5 | P20029 | Cytoplasm | -1.72 | -3.09 |
| intercellular adhesion molecule 1 | ICAM1 | P13597 | Plasma Membrane | -3.38 | -1.63 |
| interleukin 1 receptor antagonist | IL1RN | P25085 | Extracellular Space | 1.28 | 6.78 |
| integrin subunit alpha 4 | ITGA4 | Q00651 | Plasma Membrane | -1.28 | -2.46 |
| integrin subunit beta 7 | ITGB7 | P26011 | Plasma Membrane | 3.16 | 7.24 |
| Jun proto-oncogene, AP-1 transcription factor subunit | JUN | P05627 | Nucleus | 2.23 | 3.02 |
| keratin 1 | KRT1 | P04104 | Cytoplasm | -5.08 | 3.12 |
| keratin 16 | KRT16 | Q9Z2K1 | Cytoplasm | -2.59 | 2.24 |
| lymphocyte cytosolic protein 1 | LCP1 | Q61233 | Cytoplasm | -1.59 | -2.22 |
| galectin 3 | LGALS3 | P16110 | Extracellular Space | 1.41 | 3.89 |
| galectin 9B | LGALS9B | O08573 | Cytoplasm | 2.64 | 2.73 |
| lysozyme | LYZ | P17897 | Extracellular Space | -1.40 | -3.73 |
| milk fat globule-EGF factor 8 protein | MFGE8 | P21956 | Extracellular Space | 1.06 | 3.61 |
| nuclear factor kappa B subunit 1 | NFKB1 | P25799 | Nucleus | -3.36 | -1.81 |
| notch 1 | NOTCH1 | Q01705 | Plasma Membrane | -0.49 | 1.99 |
| neuropilin 2 | NRP2 | O35375 | Plasma Membrane | 1.26 | 2.31 |
| PDZ binding kinase | PBK | Q9JJ78 | Cytoplasm | -2.65 | -3.53 |
| Rho associated coiled-coil containing protein kinase 1 | ROCK1 | P70335 | Cytoplasm | -1.18 | -2.37 |
| ribosomal protein L13a | RPL13A | P19253 | Cytoplasm | -1.11 | -2.23 |
| SRC proto-oncogene, non-receptor tyrosine kinase | SRC | P05480 | Cytoplasm | -2.47 | -2.11 |
| thrombospondin 1 | THBS1 | P35441 | Extracellular Space | 3.08 | 2.94 |
| tenascin C | TNC | Q80YX1 | Extracellular Space | 3.54 | 3.00 |
| TNF receptor superfamily member 1B | TNFRSF1B | P25119 | Plasma Membrane | -2.04 | 4.52 |
| tubulin alpha 4a | TUBA4A | P68368 | Cytoplasm | -1.86 | -5.29 |
| vasoactive intestinal peptide | VIP | P32648 | Extracellular Space | -2.88 | 2.31 |

^a^Normalized signal fold change of signal in treated groups with MNPs@SiO_2_(RITC) to corresponding signal of control group

**Table S5**. Top 20 canonical pathways constructed algorithmically by Ingenuity Pathway Analysis in proteome of BV2 cells treated with MNPs*@*SiO_2_(RITC)

| Canonical pathways | -log(p-value) |
| --- | --- |
| Superpathway of Cholesterol Biosynthesis | 8.2 |
| Glucocorticoid Receptor Signaling | 7.69 |
| LXR/RXR Activation | 6.48 |
| Superpathway of Geranylgeranyldiphosphate Biosynthesis I (via Mevalonate) | 5.38 |
| Role of Macrophages, Fibroblasts and Endothelial Cells in Rheumatoid Arthritis | 4.99 |
| Mevalonate Pathway I | 4.47 |
| Aryl Hydrocarbon Receptor Signaling | 4.19 |
| Acute Phase Response Signaling | 4.08 |
| Phagosome Maturation | 4.03 |
| Phospholipase C Signaling | 4 |
| Atherosclerosis Signaling | 3.82 |
| Role of NFAT in Regulation of the Immune Response | 3.72 |
| Cholecystokinin/Gastrin-mediated Signaling | 3.63 |
| Endoplasmic Reticulum Stress Pathway | 3.59 |
| iNOS Signaling | 3.23 |
| LPS/IL-1 Mediated Inhibition of RXR Function | 3.19 |
| Apelin Endothelial Signaling Pathway | 3.13 |
| Cholesterol Biosynthesis I | 3.05 |
| Cholesterol Biosynthesis II (via 24,25-dihydrolanosterol) | 3.05 |
| Cholesterol Biosynthesis III (via Desmosterol) | 3.05 |

**Table S6**. Top 20 biological functions constructed algorithmically by Ingenuity Pathway Analysis in proteome of BV2 cells treated with MNPs*@*SiO_2_(RITC)

| Biological functions | p-value |
| --- | --- |
| Organismal Survival | 1.22 × 10^-19^ |
| Cell Death and Survival | 8.57 × 10^-15^ |
| Organismal Development | 1.28 × 10^-14^ |
| Tissue Morphology | 4.76 × 10^-14^ |
| Cardiovascular System Development and Function | 2.3 × 10^-13^ |
| Cellular Compromise | 1.33 × 10^-12^ |
| Inflammatory Response | 1.33 × 10^-12^ |
| Cellular Movement | 1.48 × 10^-12^ |
| Cancer | 2.85 × 10^-12^ |
| Organismal Injury and Abnormalities | 2.85 × 10^-12^ |
| Reproductive System Disease | 2.85 × 10^-12^ |
| Infectious Diseases | 8.47 × 10^-12^ |
| Hematological System Development and Function | 1.7 × 10^-11^ |
| Cellular Assembly and Organization | 3.12 × 10^-11^ |
| Cellular Function and Maintenance | 3.12 × 10^-11^ |
| Lymphoid Tissue Structure and Development | 4.62 × 10^-11^ |
| Embryonic Development | 4.7 × 10^-11^ |
| Tumor Morphology | 5.32 × 10^-11^ |
| Metabolic Disease | 7.48 × 10^-11^ |
| Cellular Development | 7.73 × 10^-11^ |

**Table S7.** The amount of 13 FAs, 20 AAs, and 14 OAs in MNPs@SiO_2_(RITC) treated BV2 cells

|  |  | amount (ng/2 × 10^6^ cells, ± standard deviation) | | |
| --- | --- | --- | --- | --- |
|  |  |  | MNPs@SiO_2_(RITC)-treated group (n = 3) | |
| No. | Analyte | Control  (n = 3) | 0.01 µg/µl | 0.1 µg/µl |
| **FAs** | |  |  |  |
| 1 | Myristoleic acid (C_14:1_) | 19.74 ± 0.82 | 17.99 ± 0.43 | 25.16 ± 1.12 |
| 2 | Myristic acid (C_14_) | 198.40 ± 7.60 | 178.85 ± 15.97 | 296.63 ± 27.04 |
| 3 | Palmitoleic acid (C_16:1_) | 766.11 ± 8.48 | 663.91 ± 73.78 | 970.51 ± 101.99 |
| 4 | Palmitic acid (C_16_) | 184.65 ± 14.21 | 145.03 ± 9.41 | 229.30 ± 28.04 |
| 5 | Linoleic acid (C_18:2_) | 3405.42 ± 66.06 | 2895.63 ± 226.03 | 4039.31 ± 472.73 |
| 6 | Oleic acid (C_18:1_) | 3449.87 ± 72.52 | 2989.47 ± 339.52 | 4069.75 ± 472.72 |
| 7 | Stearic acid (C_18_) | 2096.31 ± 70.59 | 1996.14 ± 194.23 | 3169.69 ± 466.52 |
| 8 | Arachidonic acid (C_20:4_) | 1309.78 ± 110.81 | 812.73 ± 93.63 | 1031.57 ± 19.02 |
| 9 | Arachidic acid (C_20_) | 42.22 ± 7.16 | 24.52 ± 0.61 | 39.03 ± 7.41 |
| 10 | Behenic acid (C_22_) | 7.44 ± 0.34 | 6.70 ± 0.26 | 7.19 ± 0.30 |
| 11 | Nervonic acid (C_24:1_) | 30.04 ± 2.27 | 16.96 ± 1.47 | 23.70 ± 3.49 |
| 12 | Lignoceric acid (C_24_) | 124.52 ± 3.77 | 113.37 ± 4.25 | 136.84 ± 9.85 |
| 13 | Cerotic acid (C_26_) | 70.70 ± 2.07 | 65.85 ± 4.56 | 90.00 ± 11.19 |
| **AAs** | |  |  |  |
| 1 | Alanine | 1055.3 ± 86.8 | 1262.1 ± 95.3 | 1118.7 ± 120.8 |
| 2 | Glycine | 290.6 ± 21.6 | 467.9 ± 53.4 | 375.4 ± 126.0 |
| 3 | Valine | 305.0 ± 8.8 | 307.5 ± 16.9 | 278.8 ± 37.9 |
| 4 | Leucine | 290.0 ± 67.7 | 178.2 ± 4.0 | 264.1 ± 20.9 |
| 5 | Isoleucine | 76.7 ± 36.3 | 68.6 ± 17.8 | 72.9 ± 28.6 |
| 6 | Proline | 463.9 ± 20.1 | 531.5 ± 17.5 | 478.7 ± 40.4 |
| 7 | γ-Aminobutric acid | 828.3 ± 37.3 | 933.8 ± 30.5 | 906.1 ± 43.7 |
| 8 | Pyroglutamic acid | 318.8 ± 23.8 | 450.2 ± 50.4 | 343.1 ± 16.0 |
| 9 | Methionine | 354.5 ± 12.7 | 319.9 ± 15.6 | 306.3 ± 32.2 |
| 10 | Serine | 177.7 ± 21.8 | 195.1 ± 39.6 | 154.3 ± 27.4 |
| 11 | Phenylalanine | 108.7 ± 12.3 | 79.0 ± 7.4 | 100.6 ± 7.8 |
| 12 | Cysteine | 325.5 ± 9.2 | 360.4 ± 38.9 | 337.0 ± 21.1 |
| 13 | Aspartic acid | 840.6 ± 24.6 | 992.3 ± 146.2 | 902.2 ± 138.6 |
| 14 | Glutamic acid | 1448.9 ± 36.3 | 1779.7 ± 118.9 | 1721.5 ± 58.8 |
| 15 | Asparagine | 578.4 ± 71.1 | 710.0 ± 101.9 | 670.5 ± 58.5 |
| 16 | Ornithine | 646.7 ± 39.5 | 911.6 ± 33.0 | 830.2 ± 86.6 |
| 17 | Glutamine | 924.3 ± 30.1 | 1038.7 ± 93.7 | 847.2 ± 80.5 |
| 18 | Lysine | 562.6 ± 12.9 | 557.5 ± 7.6 | 510.3 ± 41.5 |
| 19 | Tyrosine | 491.0 ± 14.3 | 469.2 ± 13.3 | 466.3 ± 29.7 |
| 20 | Tryptophan | 298.7 ± 2.9 | 287.5 ± 2.1 | 287.2 ± 2.2 |
| **OAs** | |  |  |  |
| 1 | 3-Hydroxybutyric acid | 19.92 ± 1.24 | 19.50 ± 1.79 | 21.45 ± 1.35 |
| 2 | Pyruvic acid | 45.83 ± 3.28 | 40.80 ± 8.38 | 65.66 ± 7.97 |
| 3 | Acetoacetic acid | 23.42 ± 0.25 | 23.57 ± 0.38 | 25.05 ± 0.42 |
| 4 | Lactic acid | 199.35 ± 53.21 | 466.33 ± 72.71 | 414.16 ± 36.51 |
| 5 | Glycolic acid | 144.81 ± 15.04 | 113.41 ± 14.68 | 177.37 ± 11.15 |
| 6 | 2-Hydroxybutyric acid | 15.61 ± 3.22 | 15.41 ± 1.83 | 15.32 ± 3.73 |
| 7 | Malonic acid | ND | ND | ND |
| 8 | Succinic acid | 24.06 ± 3.70 | 12.81 ± 3.79 | 22.94 ± 7.22 |
| 9 | Fumaric acid | 18.55 ± 1.11 | 21.33 ± 1.88 | 20.07 ± 2.91 |
| 10 | Oxaloacetic acid | 80.51 ± 0.38 | 81.20 ± 1.59 | 83.35 ± 0.47 |
| 11 | 2-Oxoglutaric acid | ND | ND | ND |
| 12 | Malic acid | 62.58 ± 4.73 | 80.19 ± 9.95 | 82.12 ± 11.24 |
| 13 | α-Hydroxyglutarate | ND | ND | ND |
| 14 | Citric acid | 52.88 ± 0.23 | 54.00 ± 1.52 | 54.28 ± 1.19 |

N.D: Not detected.

**Table S8.** The composition of 13 FAs, 20 AAs, and 14 OAs in MNPs@SiO_2_(RITC) treated BV2 cells

|  |  | Composition (%, ± standard deviation)^a^ | | |  |  |  |
| --- | --- | --- | --- | --- | --- | --- | --- |
|  |  |  | MNPs@SiO_2_(RITC)-treated group (n = 3) | |  | Ratio value^e^ | |
| No. | Analyte | Control  (n = 3) | 0.01 µg/µl | 0.1 µg/µl | *p* value^d^ | 0.01 µg/µl | 0.1 µg/µl |
| **FAs** | |  |  |  |  |  |  |
| 1 | Myristoleic acid (C_14:1_) | 0.17 ± 0.01 | 0.18 ± 0.01 (0.269) | 0.18 ± 0.01 (0.446) | 0.903 | 1.08 | 1.06 |
| 2 | Myristic acid (C_14_) | 1.67 ± 0.07 | 1.78 ± 0.12 (0.369) | 2.07 ± 0.04 (0.003) | 0.013 | 1.06 | 1.24 |
| 3 | Palmitoleic acid (C_16:1_) | 6.44 ± 0.06 | 6.58 ± 0.25 (0.548) | 6.78 ± 0.08 (0.082) | 0.326 | 1.02 | 1.05 |
| 4 | Palmitic acid (C_16_) | 1.55 ± 0.13 | 1.44 ± 0.05 (0.244) | 1.60 ± 0.03 (0.759) | 0.179 | 0.93 | 1.03 |
| 5 | Linoleic acid (C_18:2_) | 28.63 ± 0.39 | 28.73 ± 0.28 (0.922) | 28.19 ± 0.27 (0.270) | 0.166 | 1.00 | 0.98 |
| 6 | Oleic acid (C_18:1_) | 29.01 ± 0.43 | 29.61 ± 1.15 (0.586) | 28.40 ± 0.23 (0.589) | 0.179 | 1.02 | 0.98 |
| 7 | Stearic acid (C_18_) | 17.63 ± 0.54 | 19.81 ± 1.22 (0.066) | 22.07 ± 0.92 (0.003) | 0.058 | 1.12 | 1.25 |
| 8 | Arachidonic acid (C_20:4_) | 11.02 ± 0.98 | 8.13 ± 1.42 (0.048) | 7.27 ± 0.95 (0.016) | 0.646 | 0.74 | 0.66 |
| 9 | Arachidic acid (C_20_) | 0.36 ± 0.06 | 0.24 ± 0.02 (0.026) | 0.27 ± 0.02 (0.074) | 0.690 | 0.69 | 0.76 |
| 10 | Behenic acid (C_22_) | 0.25 ± 0.02 | 0.17 ± 0.02 (0.004) | 0.16 ± 0.01 (0.004) | 1.000 | 0.67 | 0.65 |
| 11 | Nervonic acid (C_24:1_) | 1.05 ± 0.03 | 1.13 ± 0.05 (0.155) | 0.96 ± 0.06 (0.139) | 0.011 | 1.08 | 0.92 |
| 12 | Lignoceric acid (C_24_) | 0.59 ± 0.01 | 0.65 ± 0.02 (0.006) | 0.63 ± 0.01 (0.090) | 0.130 | 1.10 | 1.06 |
| 13 | Cerotic acid (C_26_) | 0.29 ± 0.01 | 0.33 ± 0.01 (0.013) | 0.27 ± 0.02 (0.302) | 0.003 | 1.14 | 0.93 |
| **AAs** | |  |  |  |  |  |  |
| 1 | Alanine | 10.16 ± 0.74 | 10.60 ± 0.18 (0.580) | 10.18 ± 0.48 (0.998) | 0.615 | 1.04 | 1.00 |
| 2 | Glycine | 2.80 ± 0.19 | 3.92 ± 0.23 (0.118) | 3.38 ± 0.96 (0.481) | 0.518 | 1.40 | 1.21 |
| 3 | Valine | 2.94 ± 0.07 | 2.59 ± 0.26 (0.135) | 2.53 ± 0.18 (0.084) | 0.927 | 0.88 | 0.86 |
| 4 | Leucine | 2.79 ± 0.66 | 1.50 ± 0.12 (0.026) | 2.42 ± 0.35 (0.577) | 0.944 | 0.54 | 0.87 |
| 5 | Isoleucine | 0.74 ± 0.35 | 0.58 ± 0.18 (0.757) | 0.65 ± 0.23 (0.916) | 0.944 | 0.79 | 0.89 |
| 6 | Proline | 4.47 ± 0.15 | 4.47 ± 0.18 (1.000) | 4.36 ± 0.09 (0.651) | 0.651 | 1.00 | 0.98 |
| 7 | γ-Aminobutric acid | 7.97 ± 0.29 | 7.86 ± 0.32 (0.972) | 8.30 ± 0.99 (0.800) | 0.672 | 0.99 | 1.04 |
| 8 | Pyroglutamic acid | 3.07 ± 0.22 | 3.78 ± 0.19 (0.022) | 3.14 ± 0.27 (0.934) | 0.033 | 1.23 | 1.02 |
| 9 | Methionine | 3.41 ± 0.14 | 2.70 ± 0.29 (0.010) | 2.79 ± 0.11 (0.018) | 0.855 | 0.79 | 0.82 |
| 10 | Serine | 1.71 ± 0.20 | 1.63 ± 0.25 (0.896) | 1.40 ± 0.20 (0.271) | 0.456 | 0.95 | 0.82 |
| 11 | Phenylalanine | 1.05 ± 0.12 | 0.66 ± 0.02 (0.001) | 0.92 ± 0.04 (0.141) | 0.011 | 0.63 | 0.88 |
| 12 | Cysteine | 3.13 ± 0.11 | 3.02 ± 0.17 (0.555) | 3.07 ± 0.07 (0.837) | 0.868 | 0.96 | 0.98 |
| 13 | Aspartic acid | 8.10 ± 0.31 | 8.31 ± 0.79 (0.915) | 8.19 ± 0.75 (0.983) | 0.971 | 1.03 | 1.01 |
| 14 | Glutamic acid | 13.95 ± 0.21 | 14.96 ± 0.54 (0.089) | 15.72 ± 0.59 (0.009) | 0.203 | 1.07 | 1.13 |
| 15 | Asparagine | 5.57 ± 0.71 | 5.95 ± 0.52 (0.763) | 6.13 ± 0.68 (0.567) | 0.936 | 1.07 | 1.10 |
| 16 | Ornithine | 6.23 ± 0.39 | 7.67 ± 0.20 (0.005) | 7.56 ± 0.41 (0.007) | 0.924 | 1.23 | 1.21 |
| 17 | Glutamine | 8.90 ± 0.27 | 8.72 ± 0.24 (0.721) | 7.71 ± 0.32 (0.005) | 0.011 | 0.98 | 0.87 |
| 18 | Lysine | 5.42 ± 0.17 | 4.69 ± 0.22 (0.011) | 4.65 ± 0.21 (0.009) | 0.963 | 0.87 | 0.86 |
| 19 | Tyrosine | 4.73 ± 0.18 | 3.95 ± 0.31 (0.053) | 4.26 ± 0.41 (0.244) | 0.494 | 0.84 | 0.90 |
| 20 | Tryptophan | 2.88 ± 0.05 | 2.42 ± 0.14 (0.012) | 2.63 ± 0.17 (0.120) | 0.203 | 0.84 | 0.91 |
| **OAs** | |  |  |  |  |  |  |
| 1 | 3-Hydroxybutyric acid | 2.91 ± 0.35 | 2.11 ± 0.29 (0.021)^b^ | 2.18 ± 0.06 (0.033)^c^ | 0.933 | 0.72 | 0.75 |
| 2 | Pyruvic acid | 6.69 ± 0.63 | 4.42 ± 1.06 (0.027) | 6.68 ± 0.55 (1.000) | 0.028 | 0.66 | 1.00 |
| 3 | Acetoacetic acid | 3.42 ± 0.31 | 2.54 ± 0.15 (0.005) | 2.56 ± 0.11 (0.005) | 0.997 | 0.74 | 0.75 |
| 4 | Lactic acid | 28.70 ± 5.63 | 50.06 ± 5.86 (0.004) | 42.14 ± 1.72 (0.032) | 0.188 | 1.74 | 1.47 |
| 5 | Glycolic acid | 21.10 ± 2.00 | 12.27 ± 2.12 (0.003) | 18.11 ± 1.65 (0.221) | 0.024 | 0.58 | 0.86 |
| 6 | 2-Hydroxybutyric acid | N.D | N.D | N.D |  |  |  |
| 7 | Malonic acid | 2.29 ± 0.56 | 1.66 ± 0.17 (0.206) | 1.56 ± 0.37 (0.143) | 0.956 | 0.72 | 0.68 |
| 8 | Succinic acid | 3.54 ± 0.82 | 1.38 ± 0.42 (0.015) | 2.31 ± 0.63 (0.126) | 0.259 | 0.39 | 0.65 |
| 9 | Fumaric acid | 2.71 ± 0.28 | 2.31 ± 0.30 (0.318) | 2.05 ± 0.35 (0.093) | 0.615 | 0.85 | 0.76 |
| 10 | Oxaloacetic acid | 11.76 ± 0.92 | 8.76 ± 0.55 (0.004) | 8.51 ± 0.47 (0.003) | 0.889 | 0.74 | 0.72 |
| 11 | 2-Oxoglutaric acid | N.D | N.D | N.D |  |  |  |
| 12 | Malic acid | 9.14 ± 1.02 | 8.67 ± 1.42 (0.877) | 8.36 ± 1.00 (0.703) | 0.942 | 0.95 | 0.91 |
| 13 | α-Hydroxyglutarate | N.D | N.D | N.D |  |  |  |
| 14 | Citric acid | 7.73 ± 0.66 | 5.82 ± 0.17 (0.004) | 5.54 ± 0.34 (0.002) | 0.724 | 0.75 | 0.72 |

^a^Values as percentage of composition by each analyte amount ratio to each of the total analyte amount (ng/2 × 10^6^ cells). ^b^One way ANOVA comparing the mean values of control group and treated group with MNPs@SiO_2_(RITC) of 0.01 μg/μl. ^c^One way ANOVA comparing the mean values of control group and treated group with MNPs@SiO_2_(RITC) of 0.1 μg/μl. ^d^One way ANOVA comparing the mean values of group with MNPs@SiO_2_(RITC) of 0.01 μg/μl and treated group with MNPs@SiO_2_(RITC) of 0.1 μg/μl. ^e^Ratio values of analyte in treated groups with MNPs@SiO_2_(RITC) to corresponding mean values in the control group. N.D: Not detected.

**Table S9**. Top 20 canonical pathways constructed algorithmically by Ingenuity Pathway Analysis in metabolome of BV2 cells treated with MNPs*@*SiO_2_(RITC)

| Canonical pathways | -log(p-value) |
| --- | --- |
| TCA Cycle II (Eukaryotic) | 7.7 |
| Glycine Betaine Degradation | 6.27 |
| Folate Transformations I | 6 |
| Glycine Biosynthesis I | 5.14 |
| Acetyl-CoA Biosynthesis III (from Citrate) | 4.87 |
| Glycine Degradation (Creatine Biosynthesis) | 4.76 |
| Glutamate Degradation II | 4.76 |
| tRNA Charging | 4.58 |
| L-carnitine Biosynthesis | 4.42 |
| dTMP De Novo Biosynthesis | 4.36 |
| Tyrosine Degradation I | 4.36 |
| Sirtuin Signaling Pathway | 4.14 |
| Folate Polyglutamylation | 4.13 |
| Superpathway of Serine and Glycine Biosynthesis I | 4.08 |
| Ketolysis | 4.08 |
| Urea Cycle | 4.04 |
| Arginine Biosynthesis IV | 3.88 |
| Leukotriene Biosynthesis | 3.88 |
| Cysteine Biosynthesis III (mammalia) | 3.5 |
| Superpathway of Citrulline Metabolism | 3.45 |

**Table S10**. Top 20 biological functions constructed algorithmically by Ingenuity Pathway Analysis in metabolome of BV2 cells treated with MNPs*@*SiO_2_(RITC)

| Biological functions | p-value |
| --- | --- |
| Endocrine System Disorders | 6.94 × 10^-13^ |
| Gastrointestinal Disease | 6.94 × 10^-13^ |
| Metabolic Disease | 6.94 × 10^-13^ |
| Organismal Injury and Abnormalities | 6.94 × 10^-13^ |
| Developmental Disorder | 1.14 × 10^-12^ |
| Hereditary Disorder | 1.14 × 10^-12^ |
| Molecular Transport | 5.7 × 10^-10^ |
| Amino Acid Metabolism | 3.68 × 10^-9^ |
| Small Molecule Biochemistry | 3.68 × 10^-9^ |
| Cell Signaling | 9.17 × 10^-9^ |
| Vitamin and Mineral Metabolism | 9.17 × 10^-9^ |
| Cell-To-Cell Signaling and Interaction | 6.73 × 10^-8^ |
| Cellular Growth and Proliferation | 6.73 × 10^-8^ |
| Nervous System Development and Function | 6.73 × 10^-8^ |
| Immunological Disease | 3.9 × 10^-7^ |
| Inflammatory Disease | 3.9 × 10^-7^ |
| Inflammatory Response | 3.9 × 10^-7^ |
| Neurological Disease | 3.9 × 10^-7^ |
| Organismal Development | 1.45 × 10^-6^ |
| Cancer | 1.49 × 10^-6^ |

**Table S11**. Ingenuity Pathway Analysis-based profiles of endocytosis and exocytosis related factors of BV2 cells treated with MNPs*@*SiO_2_(RITC)

| Entrez gene name | Symbol | ID^b^ | Location | Signal fold change^a^ | |
| --- | --- | --- | --- | --- | --- |
|  |  |  |  | 0.01 μg/μl | 0.1 μg/μl |
| signal regulatory protein beta 1 | SIRPB1 | ENSMUSG00000095028 | Plasma Membrane | 1.05 | -2.95 |
| C-type lectin domain containing 7A | CLEC7A | ENSMUSG00000079293 | Plasma Membrane | 1.03 | -1.50 |
| septin 5 | SEPT5 | ENSMUSG00000072214 | Cytoplasm | -1.24 | -1.70 |
| FCH domain only 1 | FCHO1 | ENSMUSG00000070000 | Plasma Membrane | 1.00 | 4.00 |
| palladin, cytoskeletal associated protein | PALLD | ENSMUSG00000058056 | Plasma Membrane | 1.38 | 1.92 |
| unc-13 homolog D | UNC13D | ENSMUSG00000057948 | Cytoplasm | -1.83 | -1.83 |
| proteinase 3 | PRTN3 | ENSMUSG00000057729 | Extracellular Space | -1.64 | 1.61 |
| formyl peptide receptor 2 | FPR2 | ENSMUSG00000052270 | Plasma Membrane | -1.26 | -1.92 |
| potassium voltage-gated channel subfamily B member 1 | KCNB1 | ENSMUSG00000050556 | Plasma Membrane | 1.06 | -1.51 |
| LDL receptor related protein 1B | LRP1B | ENSMUSG00000049252 | Plasma Membrane | -2.45 | 4.00 |
| Rap guanine nucleotide exchange factor 4 | RAPGEF4 | ENSMUSG00000049044 | Cytoplasm | 2.40 | 2.60 |
| tripartite motif containing 72 | TRIM72 | ENSMUSG00000042828 | Cytoplasm | 1.00 | 2.00 |
| calcium/calmodulin dependent protein kinase ID | CAMK1D | ENSMUSG00000039145 | Cytoplasm | -1.20 | -2.07 |
| SRC kinase signaling inhibitor 1 | SRCIN1 | ENSMUSG00000038453 | Cytoplasm | -1.47 | -1.57 |
| pleckstrin homology and RhoGEF domain containing G6 | PLEKHG6 | ENSMUSG00000038167 | Cytoplasm | 3.63 | 3.63 |
| colony stimulating factor 3 | CSF3 | ENSMUSG00000038067 | Extracellular Space | 1.20 | 2.48 |
| prostaglandin E receptor 2 | PTGER2 | ENSMUSG00000037759 | Plasma Membrane | 1.13 | -2.25 |
| nodal growth differentiation factor | NODAL | ENSMUSG00000037171 | Extracellular Space | 2.41 | 1.88 |
| complement C1q A chain | C1QA | ENSMUSG00000036887 | Extracellular Space | -1.20 | -2.06 |
| stabilin 2 | STAB2 | ENSMUSG00000035459 | Plasma Membrane | -1.64 | 3.20 |
| toll like receptor 3 | TLR3 | ENSMUSG00000031639 | Plasma Membrane | -1.14 | -4.00 |
| synaptotagmin like 4 | SYTL4 | ENSMUSG00000031255 | Cytoplasm | 1.00 | -2.00 |
| PYD and CARD domain containing | PYCARD | ENSMUSG00000030793 | Cytoplasm | 1.29 | -1.70 |
| synaptotagmin 3 | SYT3 | ENSMUSG00000030731 | Cytoplasm | -1.13 | -4.47 |
| rabphilin 3A | RPH3A | ENSMUSG00000029608 | Plasma Membrane | 1.81 | 1.69 |
| FGR proto-oncogene, Src family tyrosine kinase | FGR | ENSMUSG00000028874 | Nucleus | -1.63 | -2.04 |
| synaptotagmin like 1 | SYTL1 | ENSMUSG00000028860 | Cytoplasm | 1.24 | 6.00 |
| colony stimulating factor 3 receptor | CSF3R | ENSMUSG00000028859 | Plasma Membrane | 1.09 | -1.55 |
| complement factor H | CFH | ENSMUSG00000026365 | Extracellular Space | 1.04 | -1.60 |
| C-C motif chemokine receptor 1 | CCR1 | ENSMUSG00000025804 | Plasma Membrane | -1.09 | 1.57 |
| CD4 molecule | CD4 | ENSMUSG00000023274 | Plasma Membrane | 1.25 | 3.94 |
| interleukin 15 receptor subunit alpha | IL15RA | ENSMUSG00000023206 | Plasma Membrane | 1.00 | -1.67 |
| MYC proto-oncogene, bHLH transcription factor | MYC | ENSMUSG00000022346 | Nucleus | 1.15 | -1.51 |
| regulator of cell cycle | RGCC | ENSMUSG00000022018 | Cytoplasm | 1.49 | 1.50 |
| platelet and endothelial cell adhesion molecule 1 | PECAM1 | ENSMUSG00000020717 | Plasma Membrane | -1.91 | -1.58 |
| integrin subunit beta 3 | ITGB3 | ENSMUSG00000020689 | Plasma Membrane | 2.00 | 2.33 |
| apolipoprotein B | APOB | ENSMUSG00000020609 | Extracellular Space | 1.00 | -1.50 |
| adenosine A2a receptor | ADORA2A | ENSMUSG00000020178 | Plasma Membrane | -2.00 | -3.00 |
| elastase, neutrophil expressed | ELANE | ENSMUSG00000020125 | Extracellular Space | 1.00 | 1.55 |
| serum/glucocorticoid regulated kinase 1 | SGK1 | ENSMUSG00000019970 | Cytoplasm | -1.10 | -2.00 |
| arachidonate 15-lipoxygenase | ALOX15 | ENSMUSG00000018924 | Cytoplasm | 1.22 | 2.39 |
| interleukin 10 | IL10 | ENSMUSG00000016529 | Extracellular Space | -1.28 | -2.11 |
| advanced glycosylation end-product specific receptor | AGER | ENSMUSG00000015452 | Plasma Membrane | 1.41 | 1.68 |
| GATA binding protein 2 | GATA2 | ENSMUSG00000015053 | Nucleus | 1.00 | 1.50 |
| RAS p21 protein activator 4 | RASA4 | ENSMUSG00000004952 | Cytoplasm | -1.07 | -1.61 |
| calponin 2 | CNN2 | ENSMUSG00000004665 | Cytoplasm | 1.00 | -1.60 |
| apolipoprotein C2 | APOC2 | ENSMUSG00000002992 | Extracellular Space | -1.30 | -1.52 |
| creatine kinase B | CKB | ENSMUSG00000001270 | Cytoplasm | -1.33 | 2.06 |
| peroxisome proliferator activated receptor gamma | PPARG | ENSMUSG00000000440 | Nucleus | 1.09 | 1.82 |
| unconventional SNARE in the ER 1 | USE1 | Q9CQ56 | Cytoplasm | -3.16 | -1.52 |
| transferrin | TF | Q921I1 | Extracellular Space | 6.16 | 3.08 |
| sorting nexin 17 | SNX17 | Q8BVL3 | Cytoplasm | -3.14 | -1.55 |
| MICAL like 1 | MICALL1 | Q8BGT6 | Cytoplasm | -1.92 | 3.03 |
| apolipoprotein A1 | APOA1 | Q00623 | Extracellular Space | 9.78 | 5.11 |
| alanyl aminopeptidase, membrane | ANPEP | P97449 | Plasma Membrane | 1.38 | 3.23 |
| ATP synthase F1 subunit beta | ATP5F1B | P56480 | Cytoplasm | -1.48 | -2.08 |
| LDL receptor related protein associated protein 1 | LRPAP1 | P55302 | Plasma Membrane | 1.39 | 2.77 |
| cyclin dependent kinase 5 | CDK5 | P49615 | Nucleus | -2.39 | -2.31 |
| ATP binding cassette subfamily A member 1 | ABCA1 | P41233 | Plasma Membrane | 1.43 | 4.08 |
| thrombospondin 1 | THBS1 | P35441 | Extracellular Space | 3.08 | 2.94 |
| CCAAT enhancer binding protein beta | CEBPB | P28033 | Nucleus | -7.26 | 3.35 |
| Fas cell surface death receptor | FAS | P25446 | Plasma Membrane | -3.73 | -1.76 |
| transglutaminase 2 | TGM2 | P21981 | Cytoplasm | 1.38 | 2.85 |
| milk fat globule-EGF factor 8 protein | MFGE8 | P21956 | Extracellular Space | 1.06 | 3.61 |
| galectin 3 | LGALS3 | P16110 | Extracellular Space | 1.41 | 3.89 |
| CD14 molecule | CD14 | P10810 | Plasma Membrane | 2.15 | 4.95 |
| heat shock protein 90 beta family member 1 | HSP90B1 | P08113 | Cytoplasm | -0.28 | -2.97 |
| albumin | ALB | P07724 | Extracellular Space | 4.27 | 1.90 |
| hemoglobin subunit alpha 2 | HBA1/HBA2 | P01942 | Extracellular Space | 5.41 | 5.86 |
|  | arachidonic acid | 506-32-1 | Other | -1.35 | -1.51 |

^a^Fold change of normalized signal in MNPs@SiO_2_(RITC) treated group relative to corresponding control group. ^b^ID for identifying the factors: Ensembl for genes, Uniprot for proteins, and CAS Registry Number for metabolite.

**Table S12**. Quantitative real-time PCR primer sequences for genes encoding transcriptomic network related genes

| **Gene Name** | **Symbol** | **NCBI Ref. seq** | **Direction** | **Primer sequence (5’-3’)** |
| --- | --- | --- | --- | --- |
| thioredoxin interacting protein | Txnip | BC011212.1 | Forward | ACATTATCTCAGGGACTTGCG |
|  |  |  | Reverse | AAGGATGACTTTCTTGGAGCC |
| integrin beta 3 | Itgb3 | BC125518.1 | Forward | CCTTGCTACTCTGCTCATCTG |
|  |  |  | Reverse | ATATTGGTGAAGGTGGAGGTG |
| oxidized low density lipoprotein (lectin-like) receptor 1 | Olr1 | NM_138648.2 | Forward | CTCAACCCCAGTTTCTCTTCC |
|  |  |  | Reverse | TGTAACTTGAGATGCTGAGGC |
| peroxisome proliferator activated receptor gamma | Pparg | BC021798.1 | Forward | CTCTGCTCAAGTATGGTGTCC |
|  |  |  | Reverse | GCTCCATAAAGTCACCAAAGG |
| protein kinase C, theta | Prkcq | BC138552.1 | Forward | ACCACGCCTACTTCACTAATG |
|  |  |  | Reverse | TCTTTCACAGCATCGGGAAC |
| TNF receptor superfamily member 6 | Fas | BC061160.1 | Forward | AAGTCCCAGAAATCGCCTATG |
|  |  |  | Reverse | GTATGGTTTCACGACTGGAGG |
| apelin | Apln | BC020015.1 | Forward | TCTCCGTCTTTGTCGTTTCTG |
|  |  |  | Reverse | TTTACTCCCCTCTTGTGCTTC |
| myelocytomatosis oncogene | Myc | BC138931.1 | Forward | ATTGATGTGGTGTCTGTGGAG |
|  |  |  | Reverse | GTAGTTGTGCTGGTGAGTGG |
| potassium voltage-gated channel, shaker-related subfamily, member 3 | Kcna3 | BC137667.1 | Forward | TGGTTCTCCTTTGAGCTTCTG |
|  |  |  | Reverse | TTACCTTGTCGTTCAGCCAG |
| solute carrier family 1 (glial high affinity glutamate transporter), member 2 | Slc1a2 | BC138255.1 | Forward | GATCACTGCTCTGGGAACTG |
|  |  |  | Reverse | TTAATGGTTGCTCCGACTGG |
| glyceraldehyde-3-phosphate dehydrogenase | Gapdh | NM_001289726.1 | Forward | GAAGACTGTGGATGGCCC |
|  |  |  | Reverse | CCATGCCAGTGAGCTTCC |

Ref. seq.: Reference sequence


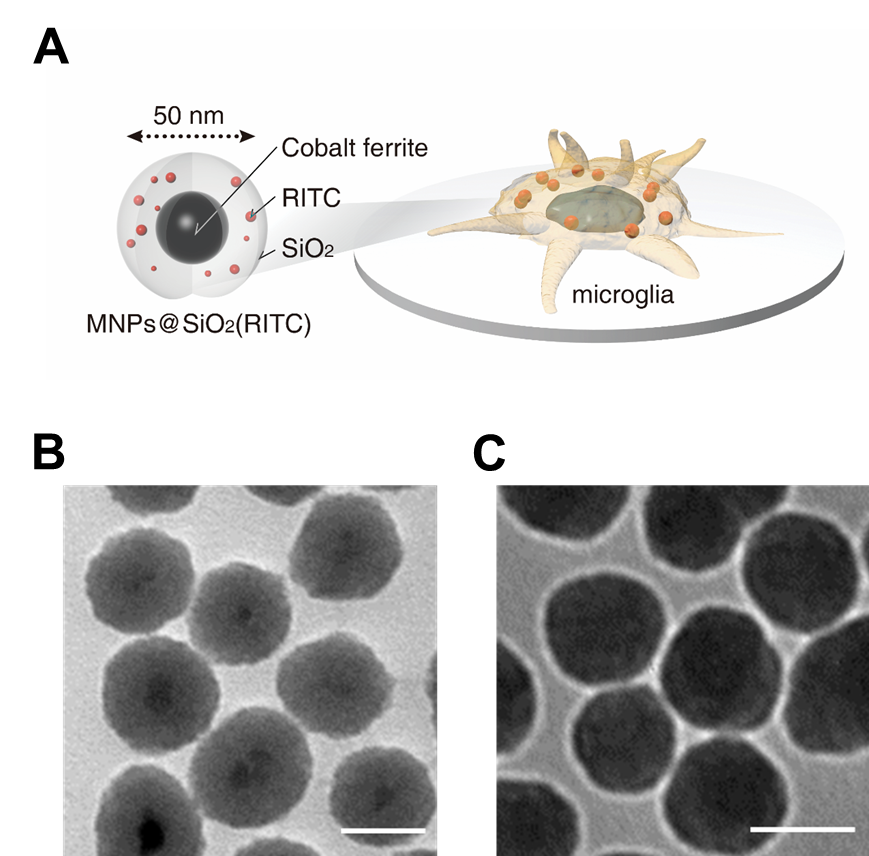


**Fig. S1.** MNPs@SiO_2_(RITC) characterization. **a** Schematic of MNPs@SiO_2_(RITC) composition. Transmission electron microscope images for **b** MNPs@SiO_2_(RITC) and **c** silica NPs. Scale bar = 50 nm.


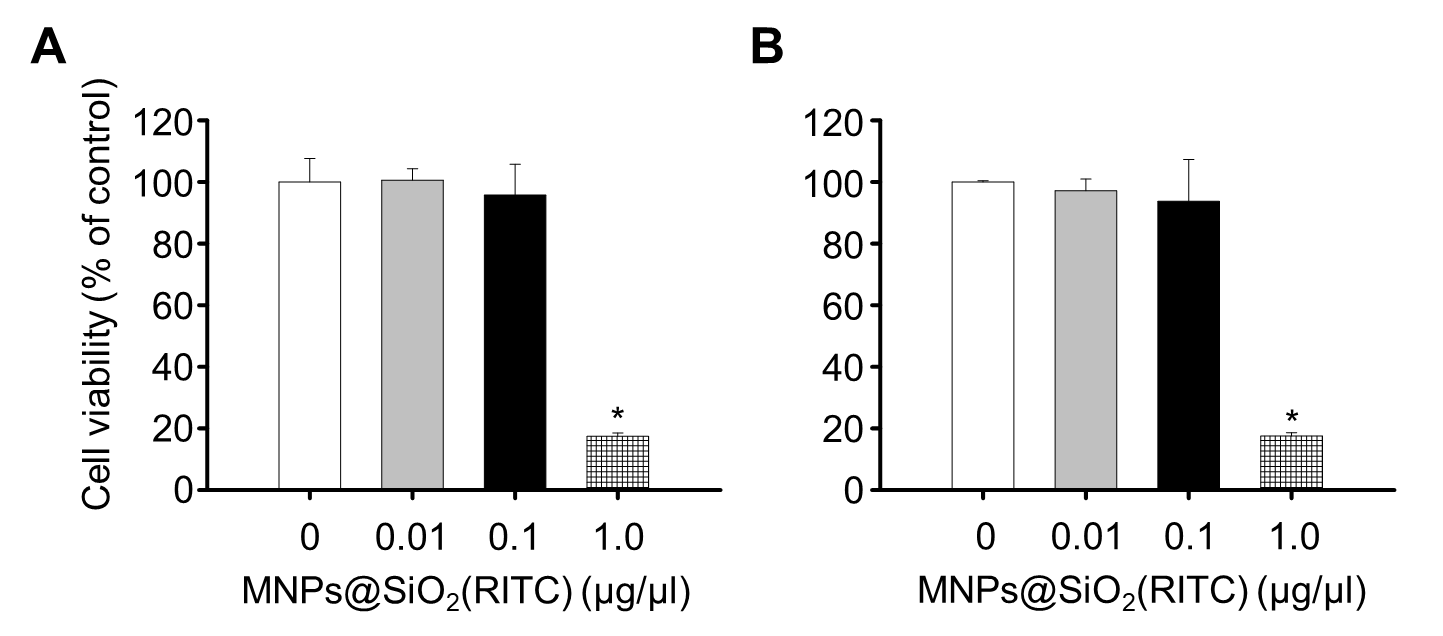


**Fig. S2.** Cell viability of MNPs@SiO_2_(RITC) in microglia. Cell viability were analyzed after treatment with MNPs@SiO_2_(RITC) for 12 h in BV2 **a** and primary rat microglia **b**. Data represent means ± standard deviation of 3 independent experiments. **p* < 0.05 vs. control.


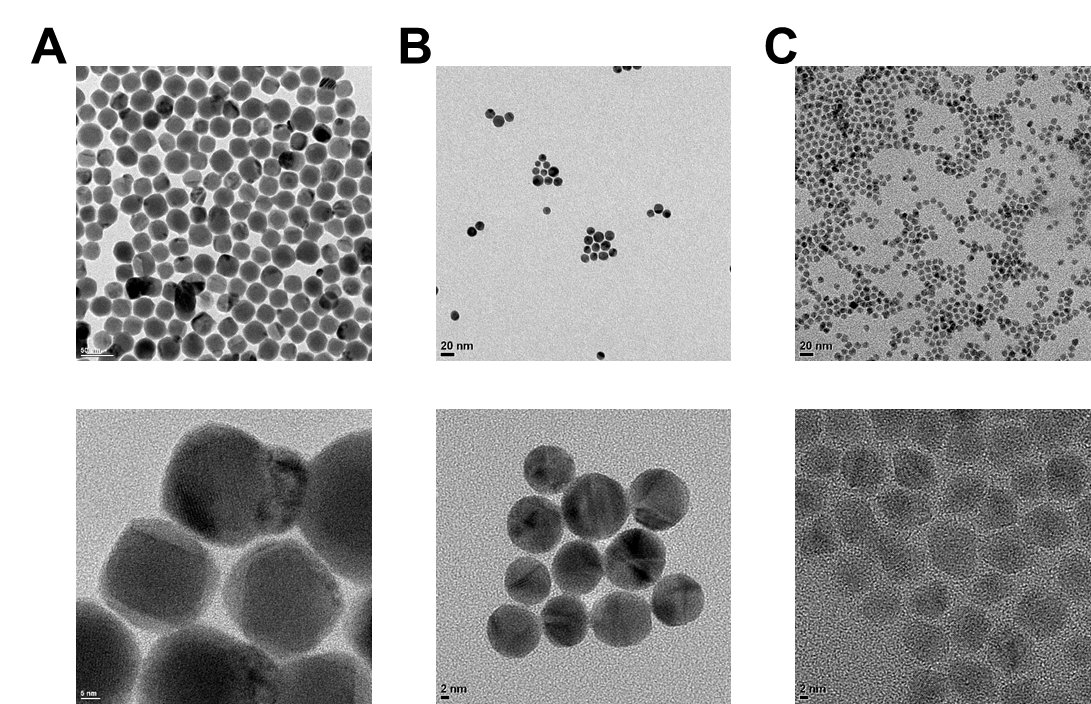


**Fig. S3.** Transmission electron microscope images for **a** Ag NPs, **b** Au NPs, and **c** CdSe QDs.


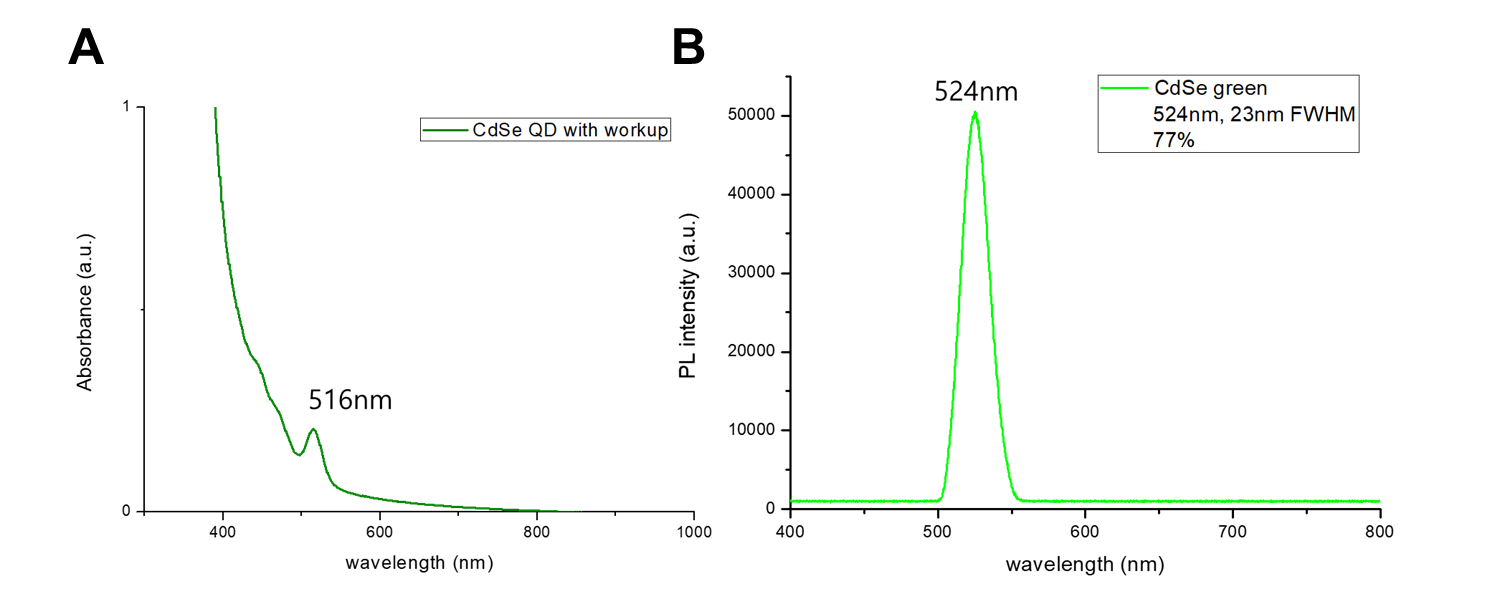


**Fig. S4.** Absorption **a** and emission **b** spectrum of CdSe QDs. PL = Photoluminescence.


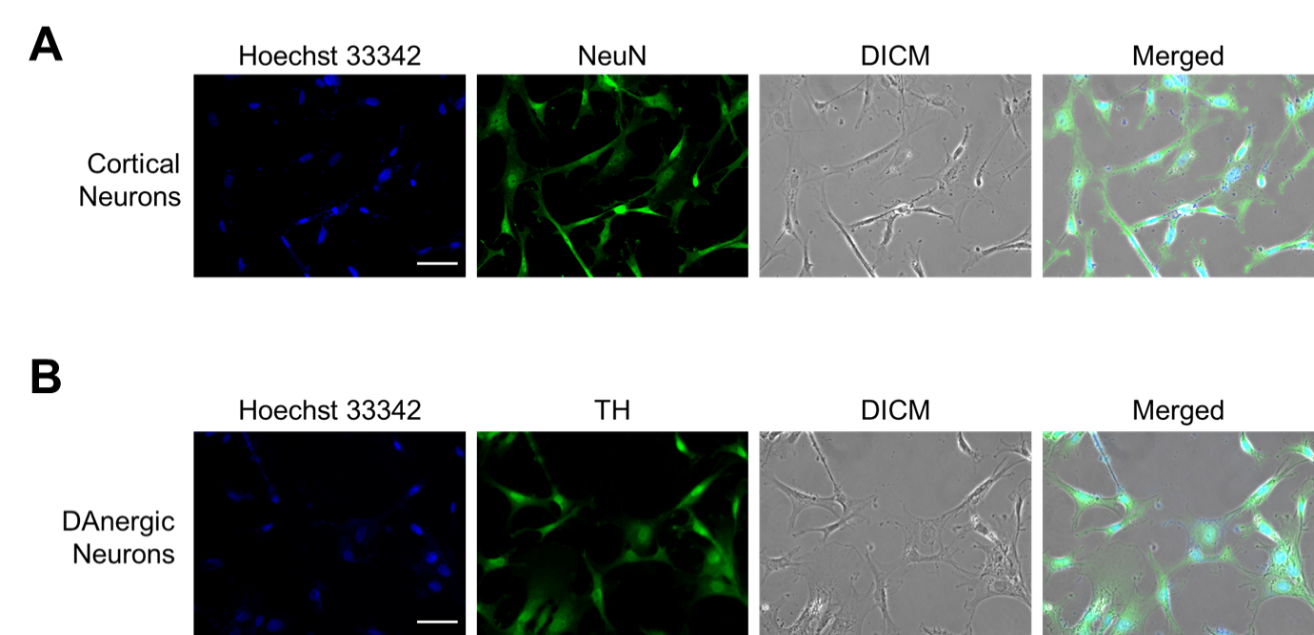


**Fig. S5.** Characterization of primary rat cortical and dopaminergic neurons. Primary rat cortical **a** and dopaminergic neurons **b** were immunostained with specific marker protein. Immunofluorescence of cortical neurons stained with anti-NeuN antibody. Immunofluorescence of dopaminergic neurons stained with anti-TH antibody. DICM: differential interference contrast microscopy. Scale bar = 50 μm.


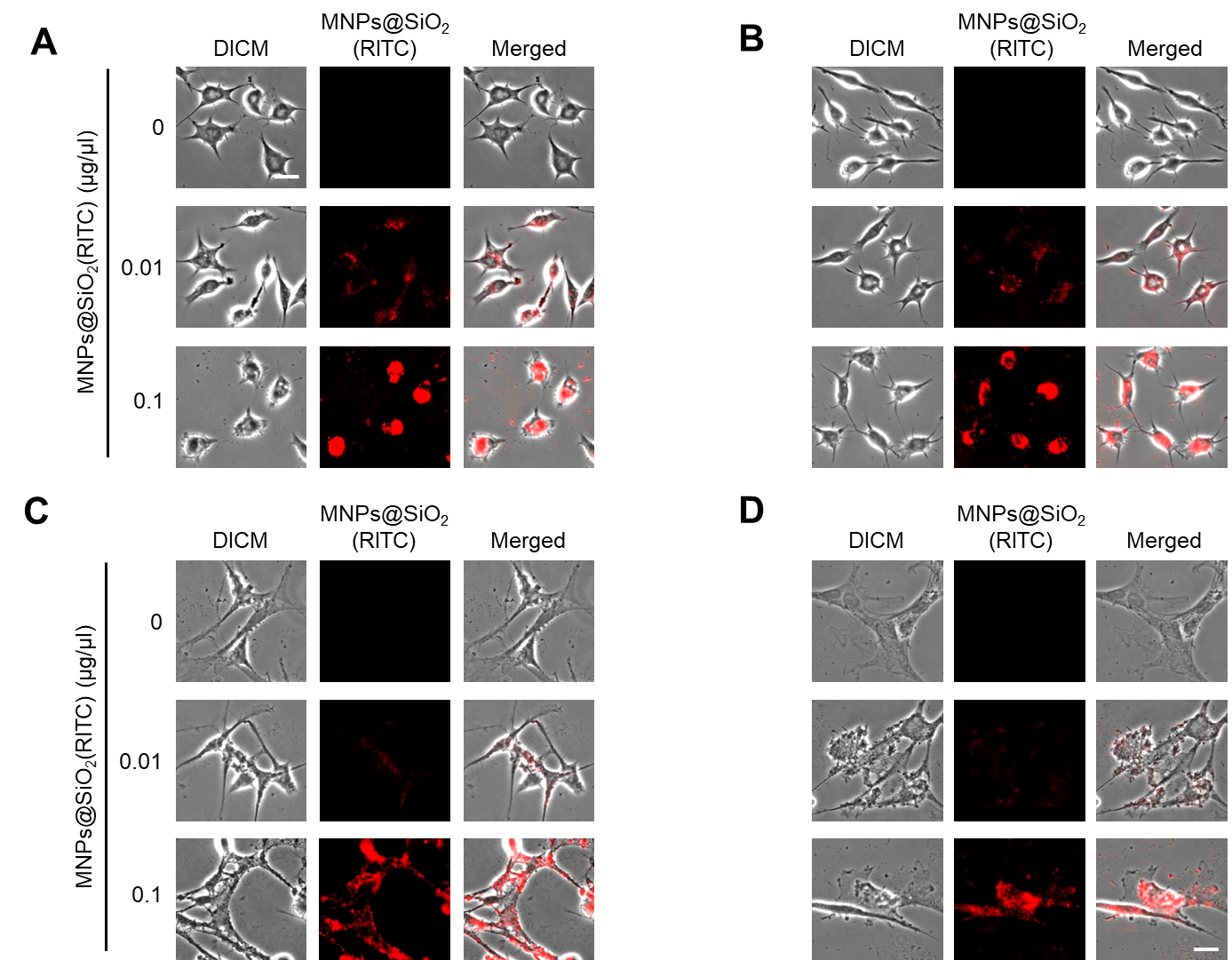


**Fig. S6.** Comparison of uptake efficiency for MNPs@SiO_2_(RITC) in microglia and neurons. Cells were treated with MNPs@SiO_2_(RITC) for 12 h, and the nanoparticles were detected with fluorescence in BV2 **a**, primary rat microglia **b**, cortical neurons **c**, and dopaminergic neurons **d**. Scale bar = 20 µm. Although the differences in uptake efficiency for MNPs@SiO_2_(RITC) are hard to be determined at 0.1 µg/µl concentration due to fluorescence saturation, microglial cells showed higher uptake efficiency than neuronal cells.


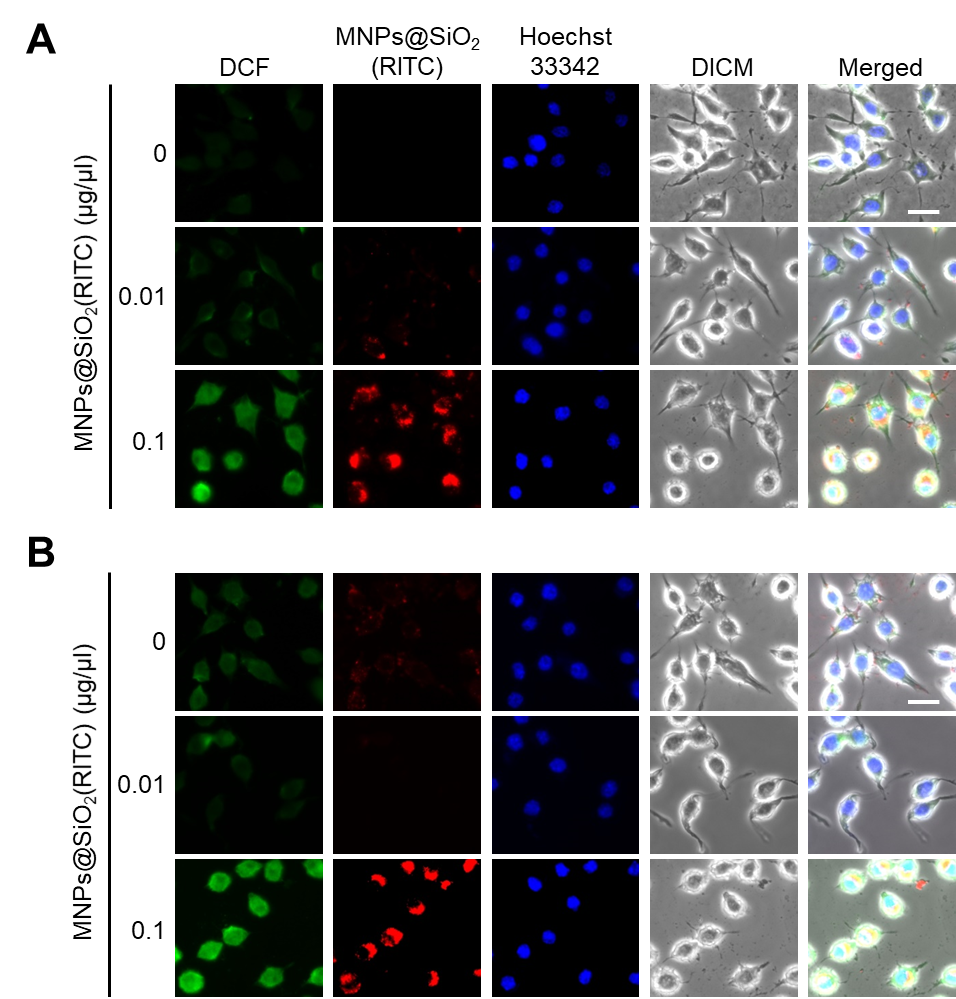


**Fig. S7.** Intracellular ROS generation in MNPs@SiO_2_(RITC) treated microglia. Intracellular ROS was evaluated using DCFH-DA (DCF) staining method. Cells were treated with MNPs@SiO_2_(RITC) for 12 h and ROS levels were detected with fluorescence of DCF in BV2 **a** and primary rat microglia **b**. Scale bar = 20 µm.


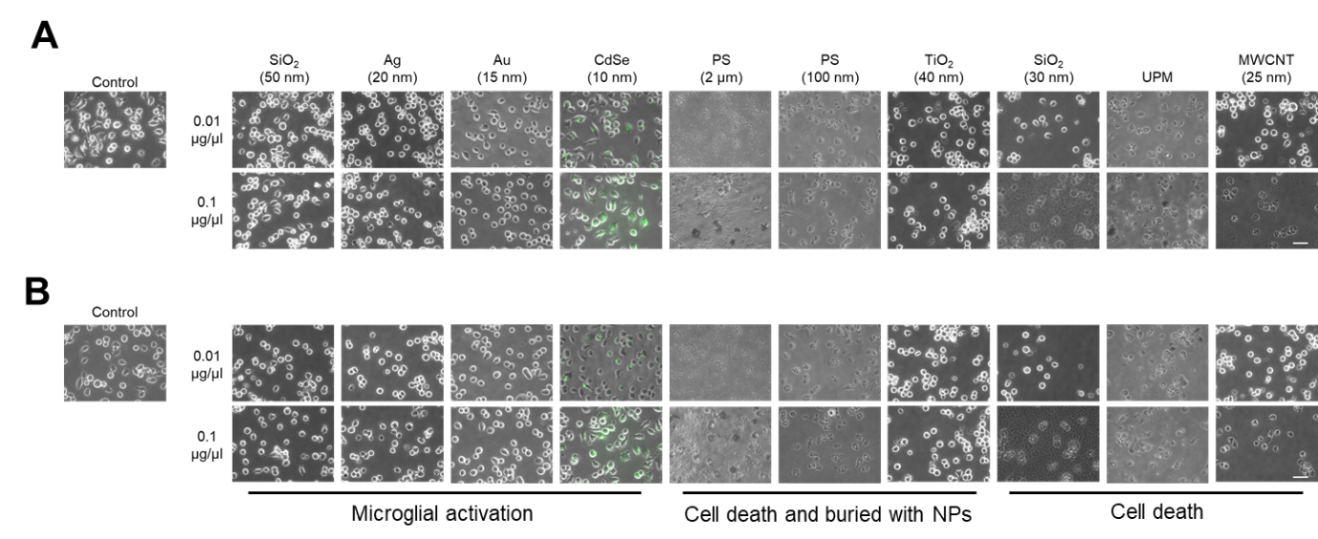


**Fig. S8.** Response of microglia to 10 kinds of nanomaterials. BV2 cells were treated with 10 kinds of nanomaterials (at doses 0.01 and 0.1 μg/μl), *e.g*. 50 nm silica NPs (SiO_2_ NPs), 20 nm silver NPs (Ag NPs), 15 nm gold NPs (Au NPs), cadmium selenide quantum dots (CdSe QDs), 2 μm and 100 nm polystyrene particles (PSs), 40 nm titanium dioxide NPs (TiO_2_ NPs), 30 nm SiO_2_ NPs, urban particulate matter (UPM), and 25 nm outer diameter (OD) multi-walled carbon nanotubes (MWCNTs). Cells were treated with nanomaterials for 12 h and the morphological changes and cell density were analyzed in BV2 **a** and primary rat microglia **b**. Scale bar = 50 µm.


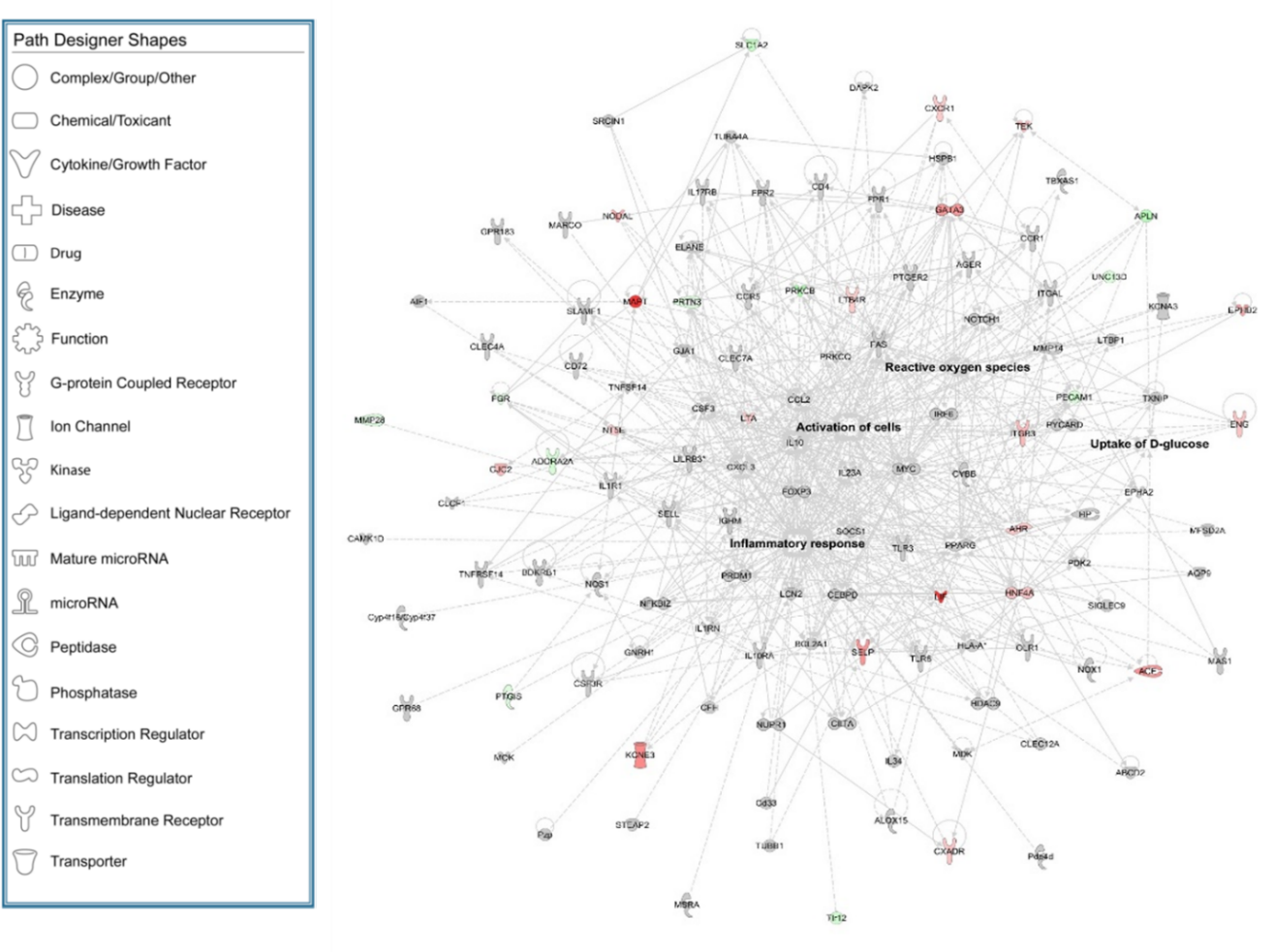


**Fig. S9.** Functional analysis of transcriptomic network of 0.01 µg/µl MNPs@SiO_2_(RITC) treated BV2 cells using IPA. Fold change ± 1.5 was used as cut off value. Red and green areas indicate up- and downregulated genes, respectively. Path Designer Shapes are originated from Ingenuity Systems (http://www.ingenuity.com).

**
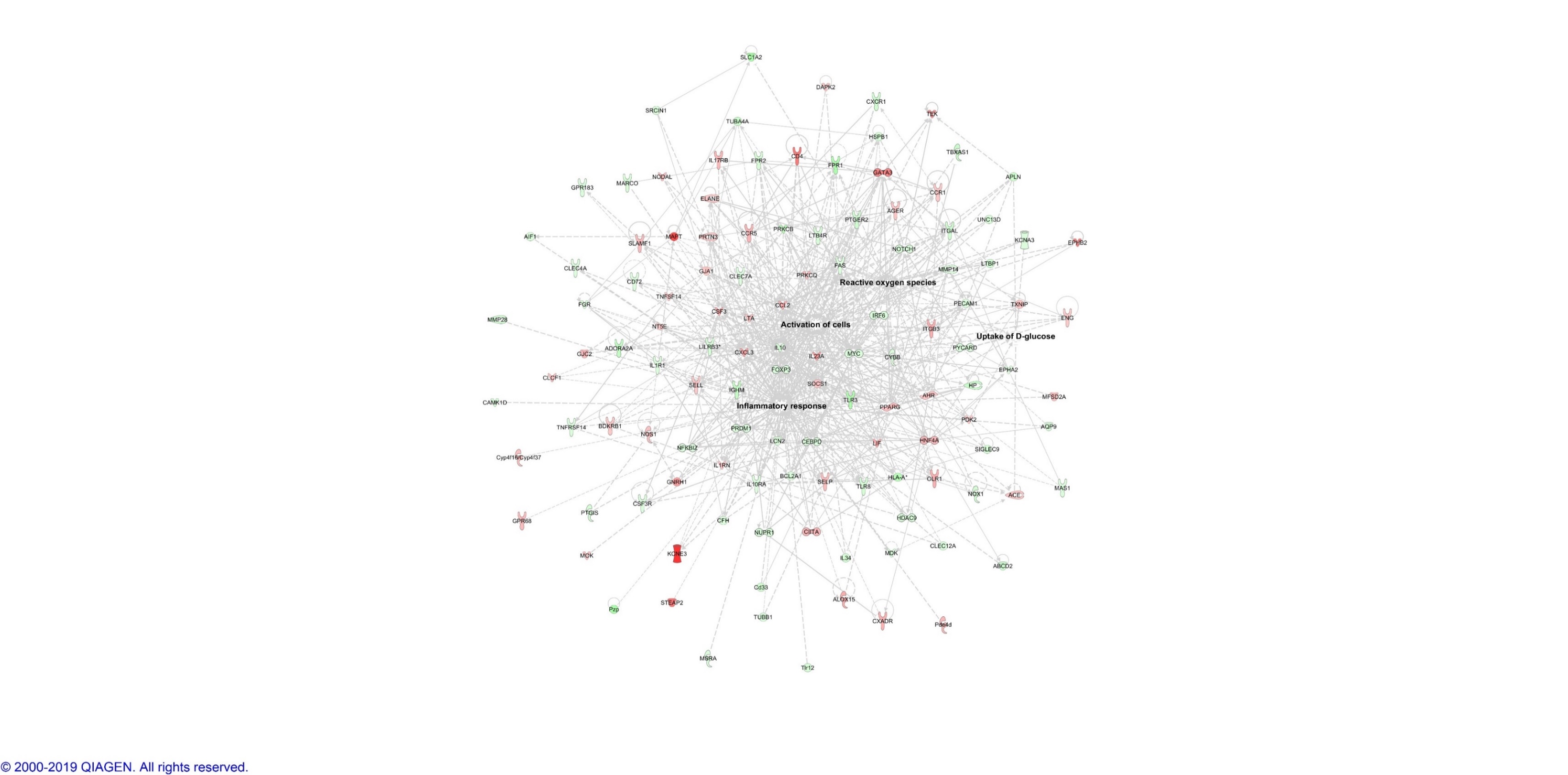
**

**Fig. S10.** Functional analysis of transcriptomic network of 0.1 µg/µl MNPs@SiO_2_(RITC) treated BV2 cells. Fold change ± 1.5 was used as cut off value. Red and green areas indicate up- and downregulated genes, respectively. Symbols are described in the legend of Fig. S9.


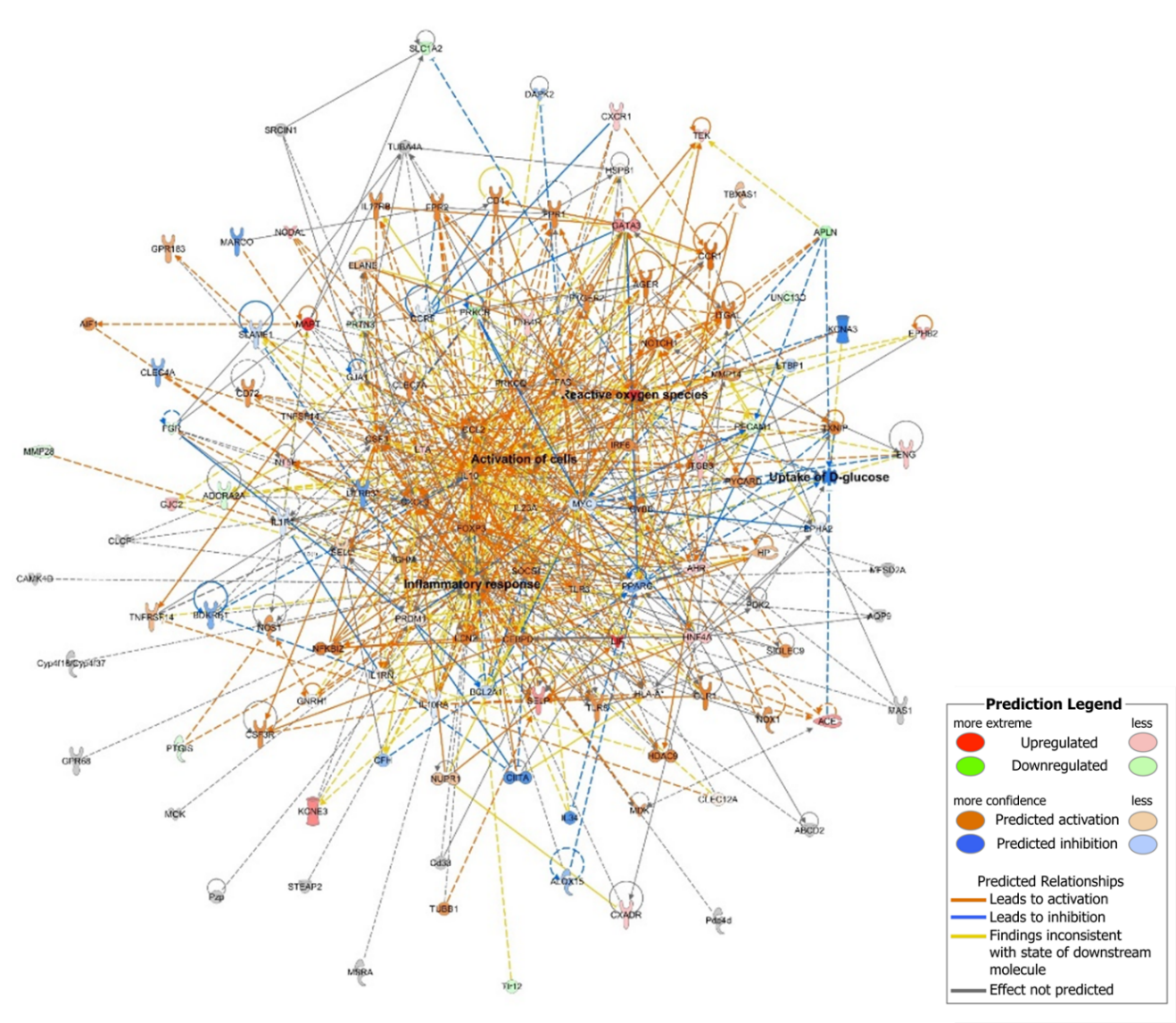


**Fig. S11**. Functional analysis of transcriptomic network with prediction of 0.01 µg/µl MNPs@SiO_2_(RITC) treated BV2 cells. Fold change ± 1.5 was used as cut off value. Red and green areas indicate up- and downregulated genes, respectively. Orange and blue areas indicate prediction as activation and inhibition, respectively. Prediction Legends are originated from Ingenuity Systems (http://www.ingenuity.com). Symbols are described in the legend of Fig. S9.


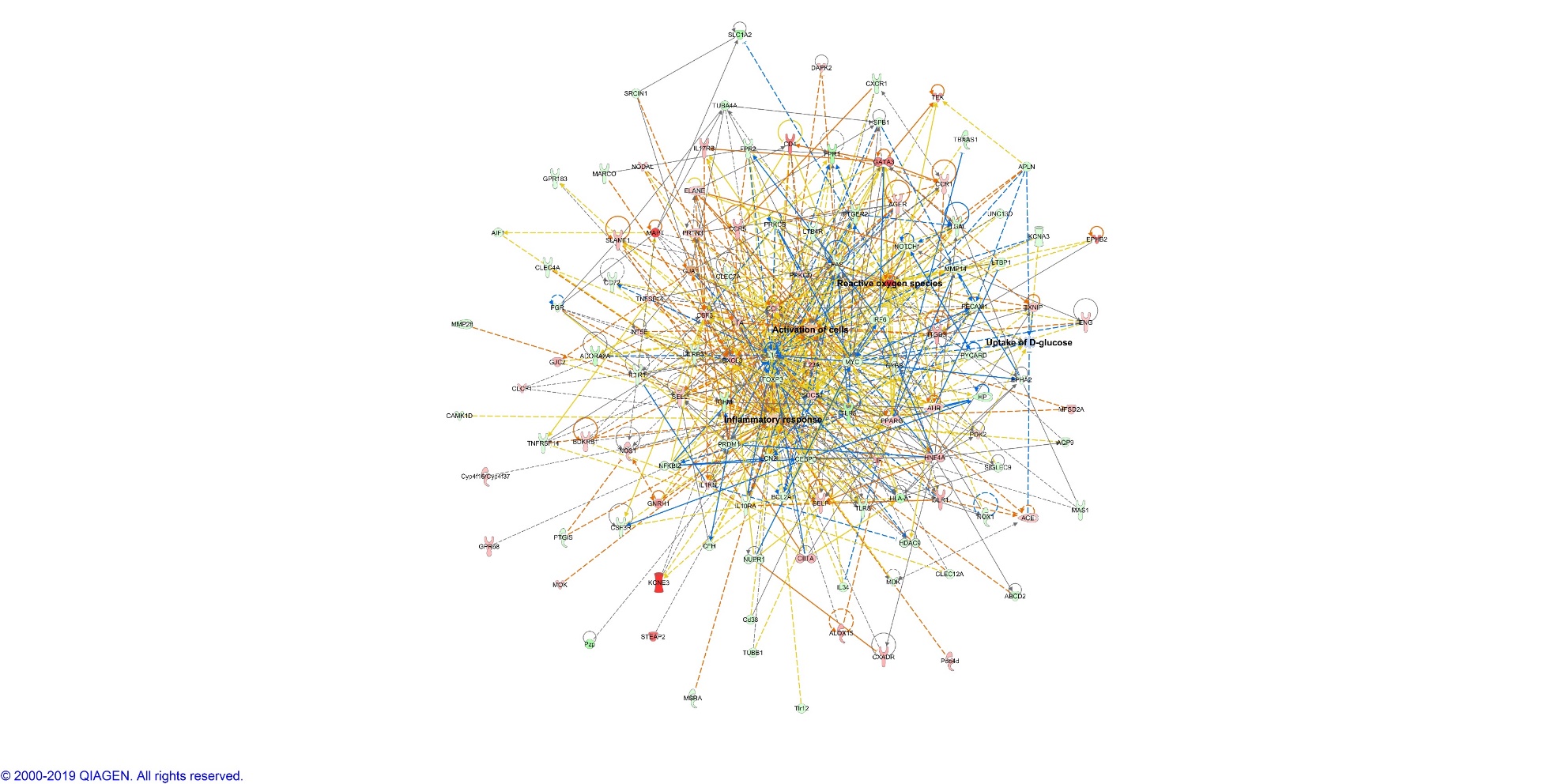


**Fig. S12**. Functional analysis of transcriptomic network with prediction of 0.1 µg/µl MNPs@SiO_2_(RITC) treated BV2 cells. Fold change ± 1.5 was used as cut off value. Red and green areas indicate up- and downregulated genes, respectively. Orange and blue areas indicate prediction as activation and inhibition, respectively. Details for shape and color are provided in Figs. S9 and S11.


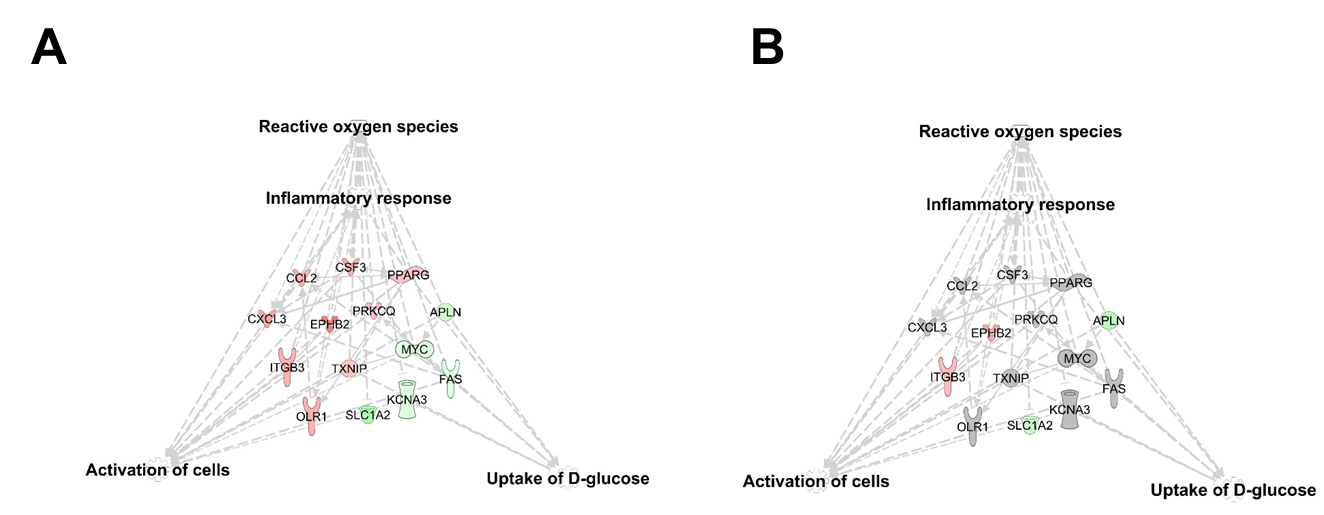


**Fig. S13**. Functional analysis of trimmed transcriptomic network of 0.1 **a** and 0.01 **b** µg/µl MNPs@SiO_2_(RITC) treated BV2 cells. Fold change ± 1.5 was used as cut off value. Red and green areas indicate up- and downregulated genes, respectively. Symbols are described in the legend of Fig. S9.


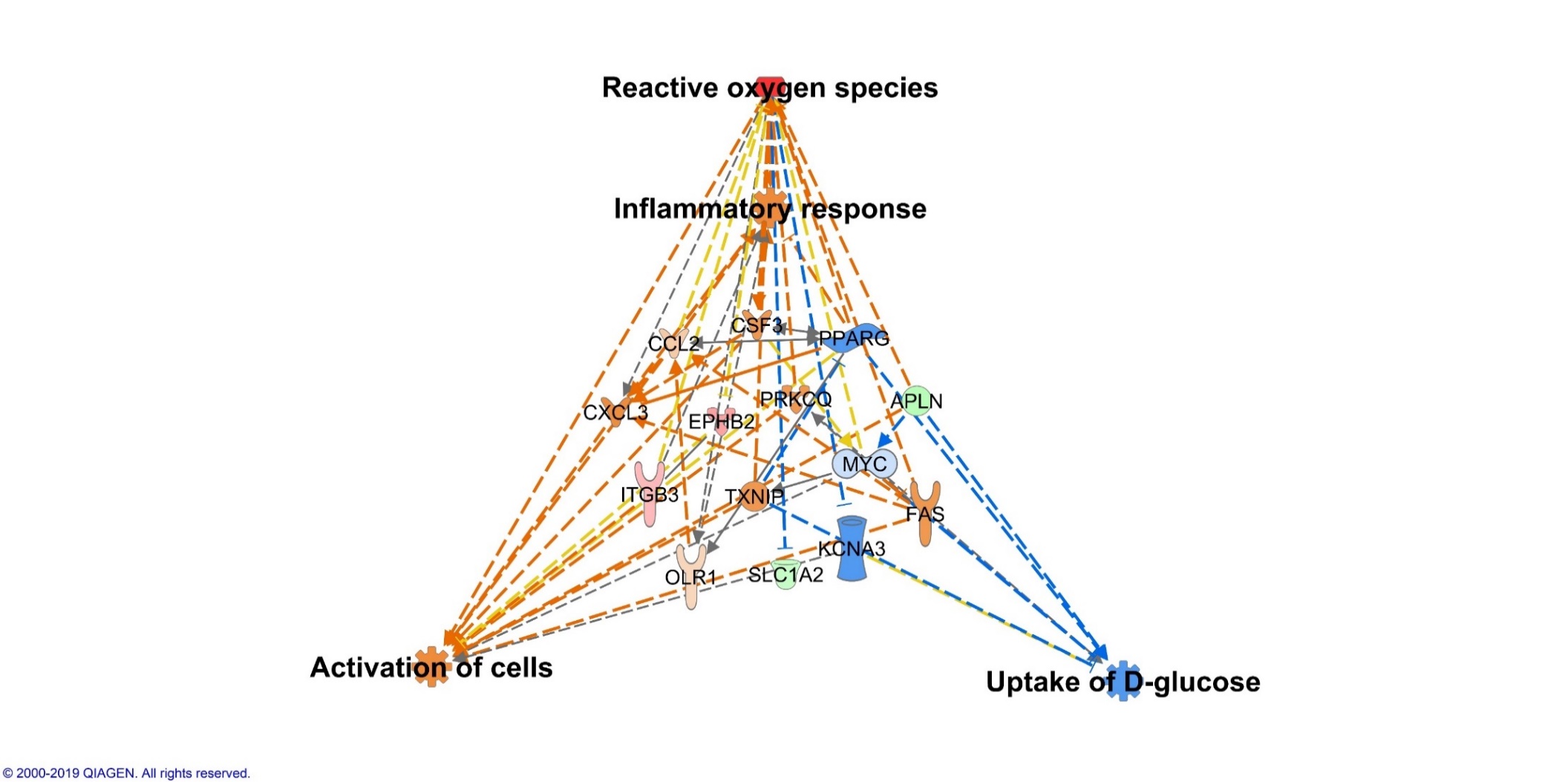


**Fig. S14**. Functional analysis of trimmed transcriptomic network with prediction of 0.01 µg/µl MNPs@SiO_2_(RITC) treated BV2 cells. Fold change ± 1.5 was used as cut off value. Details for shape and color are provided in Figs. S9 and S11.


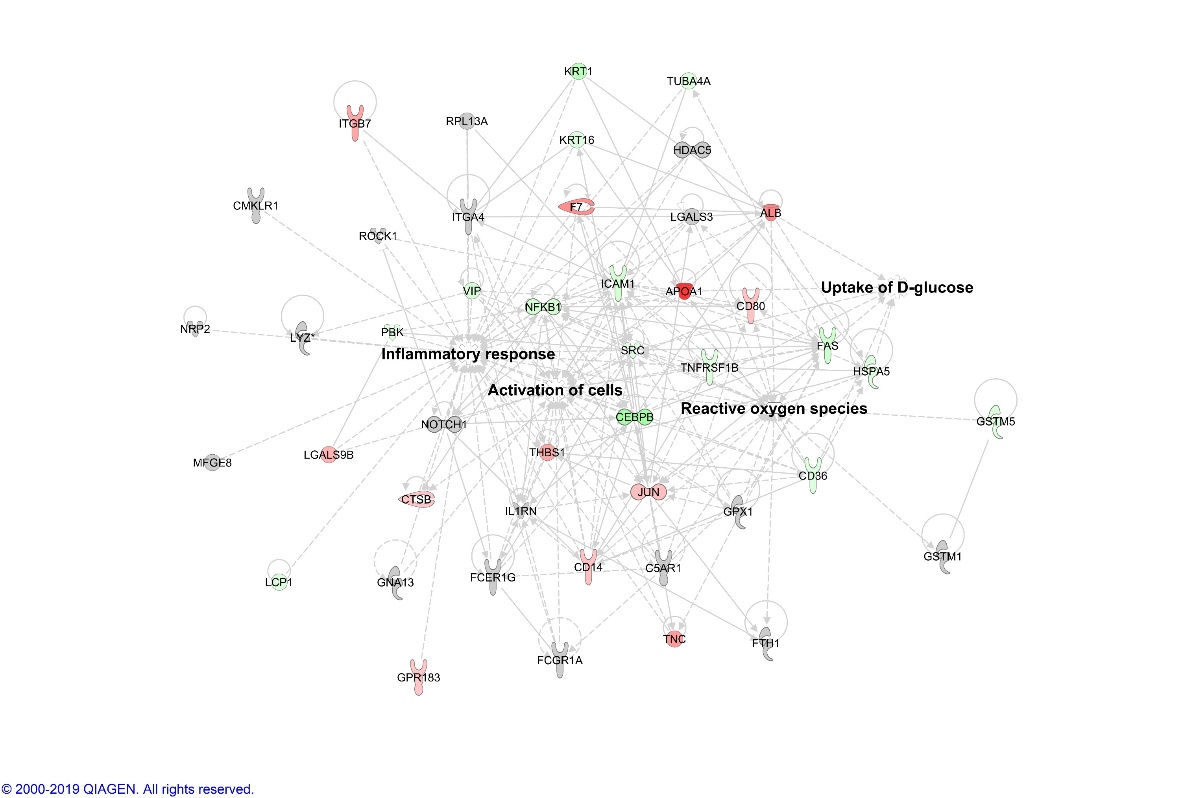


**Fig. S15.** Functional analysis of proteomic network of 0.01 µg/µl MNPs@SiO_2_(RITC) treated BV2 cells. Fold change ± 1.5 was used as cut off value. Red and green areas indicate up- and downregulated proteins, respectively. Symbols are described in the legend of Fig. S9.


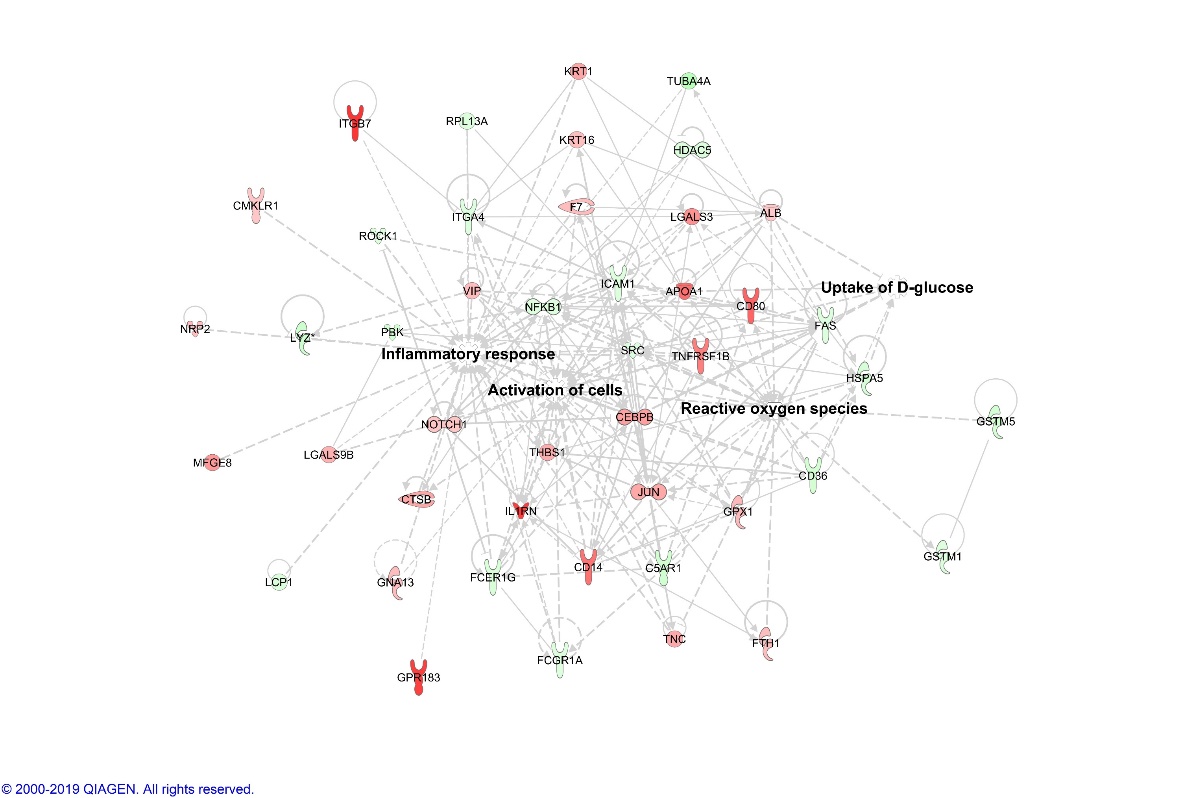


**Fig. S16.** Functional analysis of proteomic network of 0.1 µg/µl MNPs@SiO_2_(RITC) treated BV2 cells. Fold change ± 1.5 was used as cut off value. Red and green areas indicate up- and downregulated proteins, respectively. Symbols are described in the legend of Fig. S9.


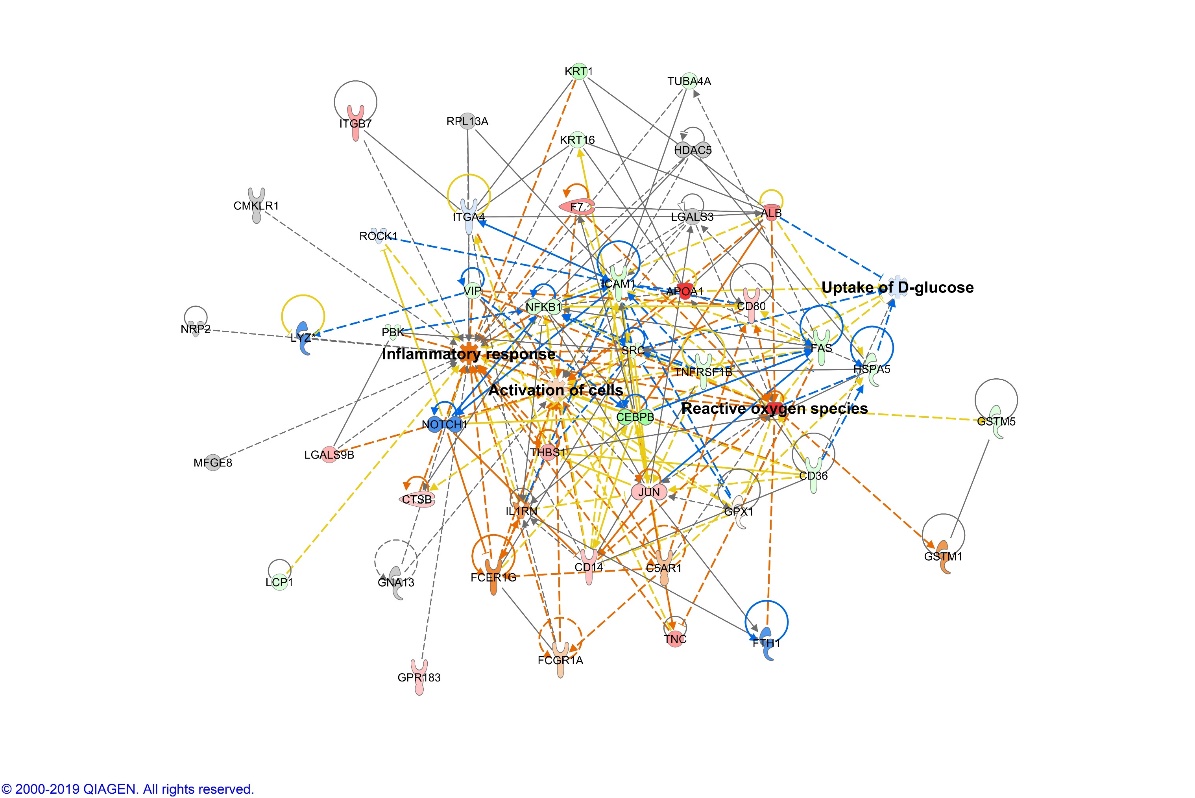


**Fig. S17**. Functional analysis of proteomic network with prediction of 0.01 µg/µl MNPs@SiO_2_(RITC) treated BV2 cells. Fold change ± 1.5 was used as cut off value. Details for shape and color are provided in Figs. S9 and S11.


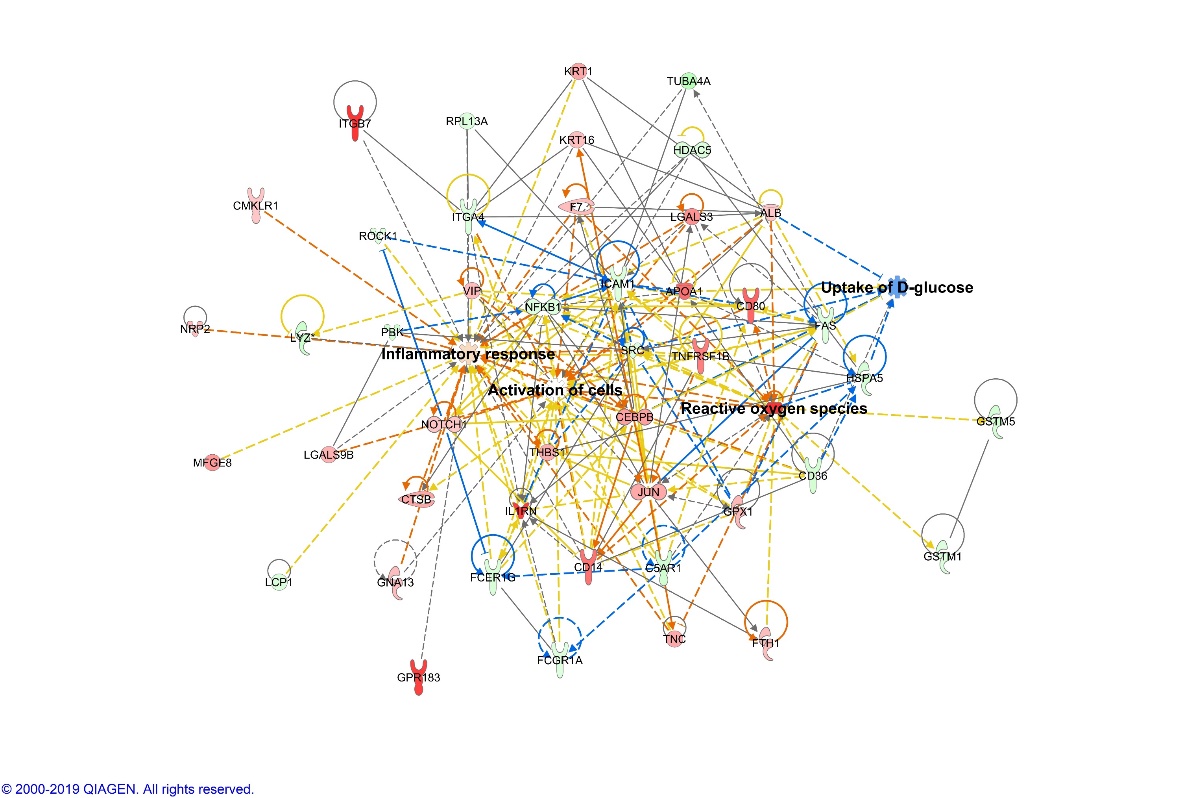


**Fig. S18**. Functional analysis of proteomic network with prediction of 0.1 µg/µl MNPs@SiO_2_(RITC) treated BV2 cells. Fold change ± 1.5 was used as cut off value. Details for shape and color are provided in Figs. S9 and S11.


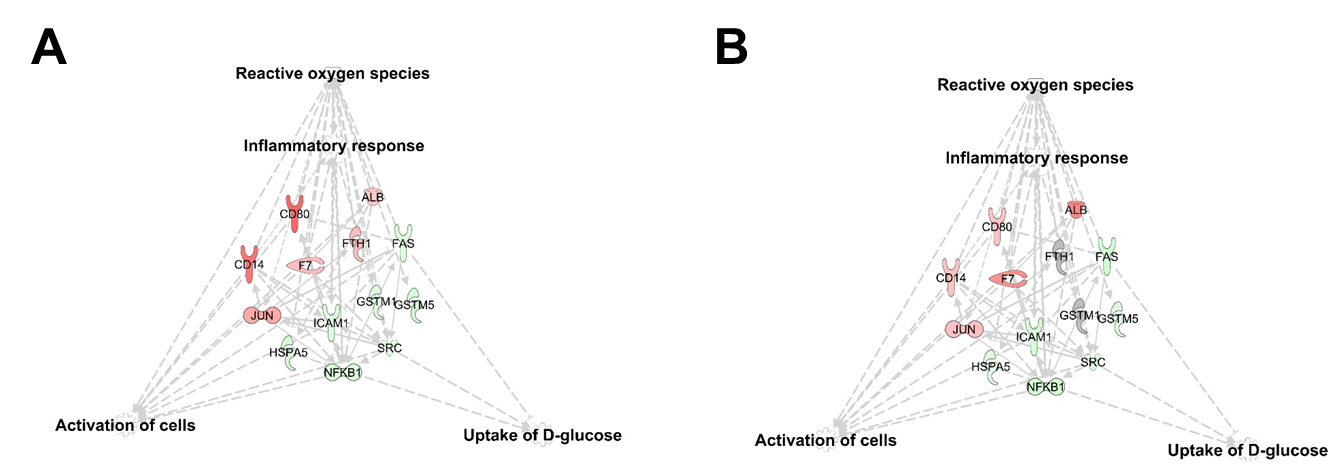


**Fig. S19**. Functional analysis of trimmed proteomic network of 0.1 **a** and 0.01 **b** µg/µl MNPs@SiO_2_(RITC) treated BV2 cells. Fold change ± 1.5 was used as cut off value. Symbols are described in the legend of Fig. S9.


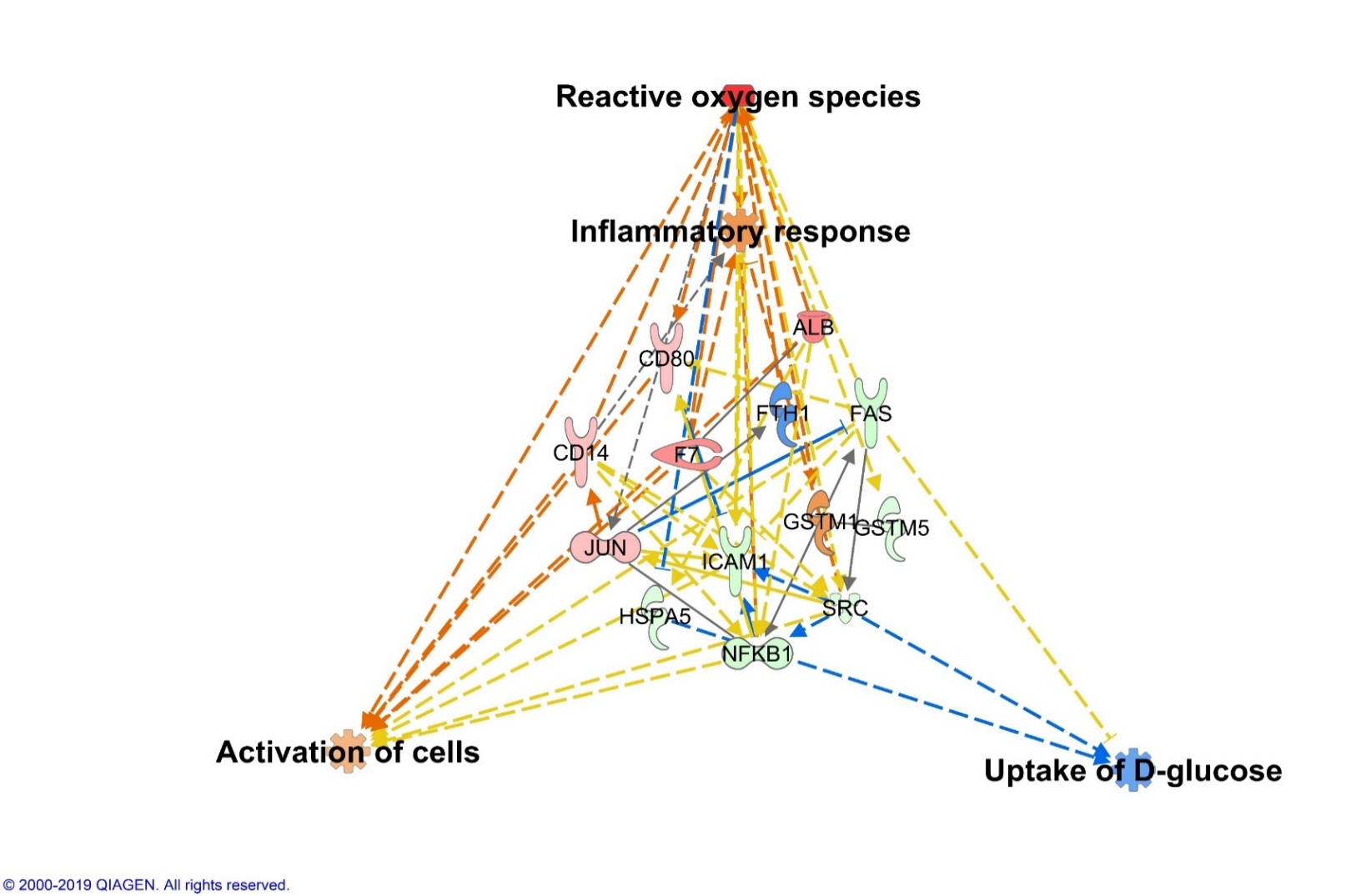


**Fig. S20**. Functional analysis of trimmed proteomic network with prediction of 0.01 µg/µl MNPs@SiO_2_(RITC) treated BV2 cells. Fold change ± 1.5 was used as cut off value. Details for shape and color are provided in Figs. S9 and S11.


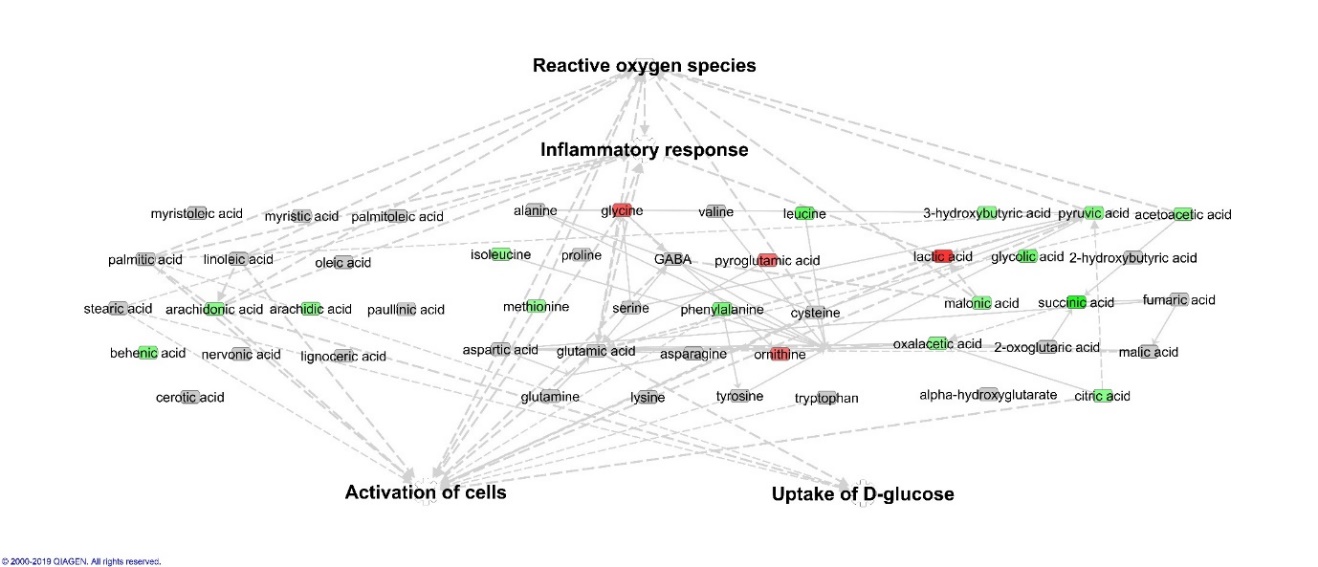


**Fig. S21**. Functional analysis of metabolic network of 0.01 µg/µl MNPs@SiO_2_(RITC) treated BV2 cells. Fold change ± 1.2 was used as cut off value. Red and green areas indicate up- and downregulated metabolites, respectively. Symbols are described in the legend of Fig. S9.


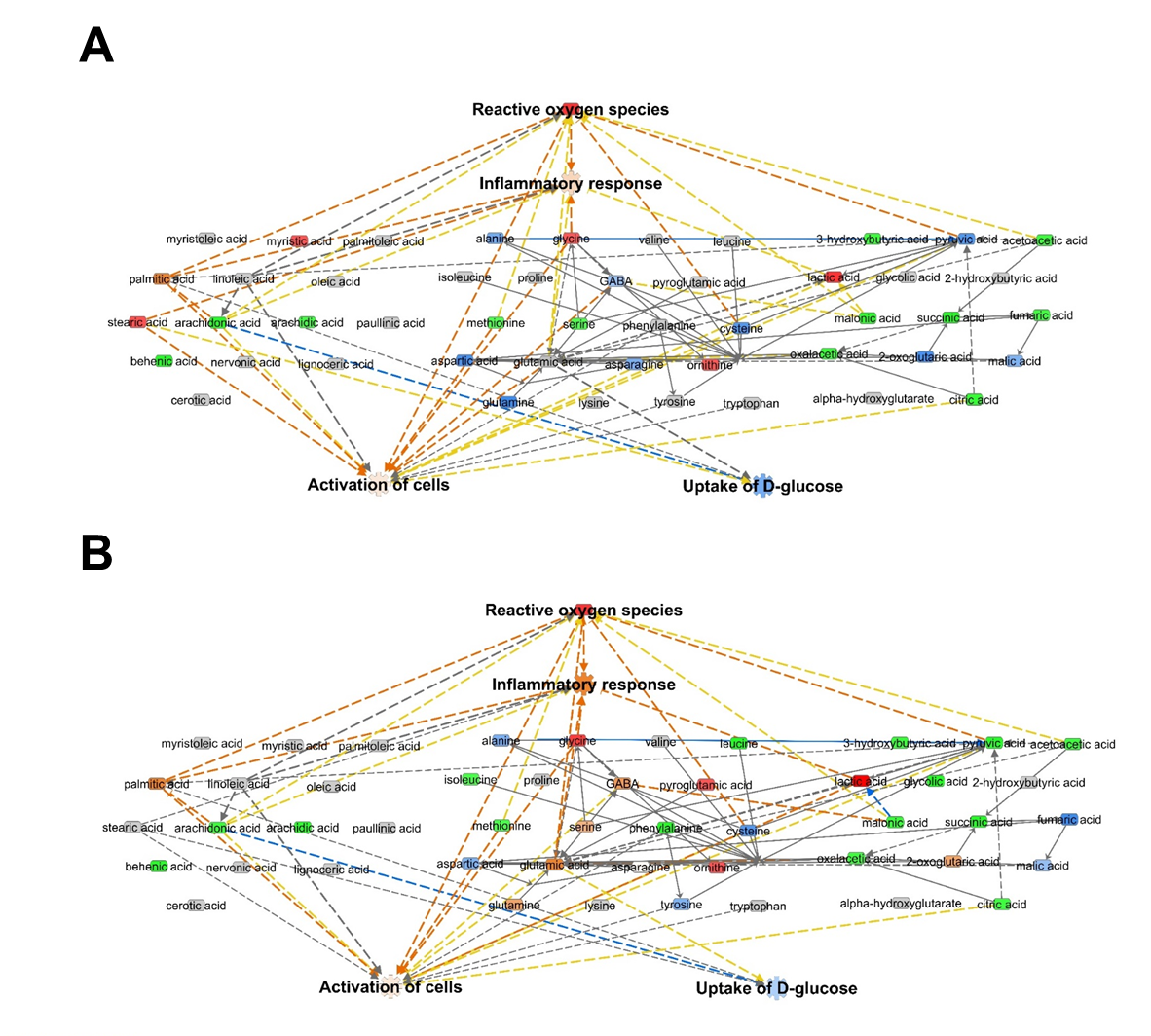


**Fig. S22**. Functional analysis of metabolic network with prediction of 0.1 **a** and 0.01 **b** µg/µl MNPs@SiO_2_(RITC) treated BV2 cells. Fold change ± 1.2 was used as cut off value. Red and green areas indicate up- and downregulated metabolites, respectively. Orange and blue areas indicate prediction as activation and inhibition, respectively. Details for shape and color are provided in Figs. S9 and S11.


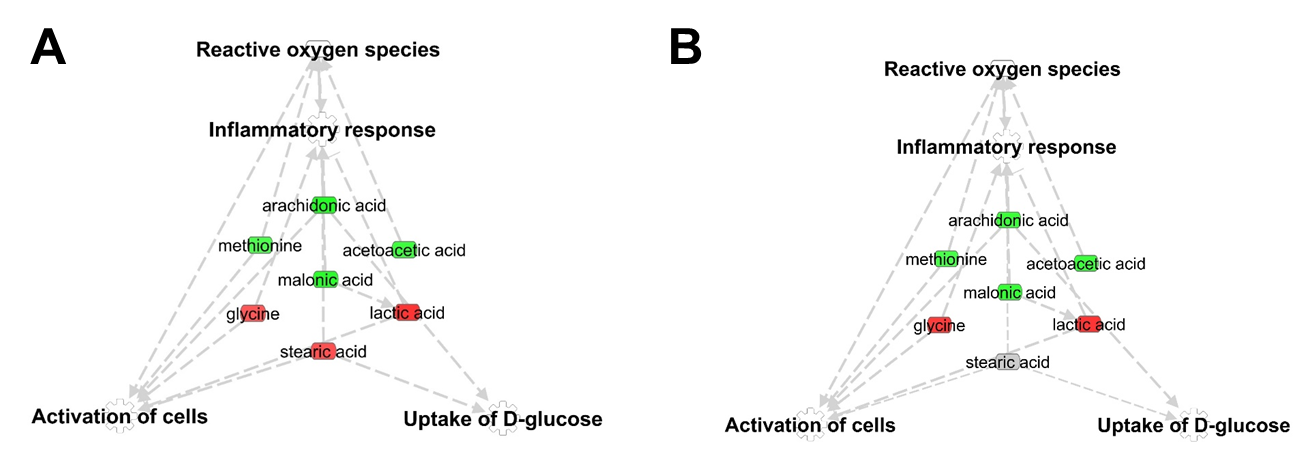


**Fig. S23**. Functional analysis of trimmed metabolic network of 0.1 **a** and **b** 0.01 µg/µl MNPs@SiO_2_(RITC) treated BV2 cells. Fold change ± 1.2 was used as cut off value. Red and green areas indicate up- and downregulated metabolites, respectively. Symbols are described in the legend of Fig. S9.


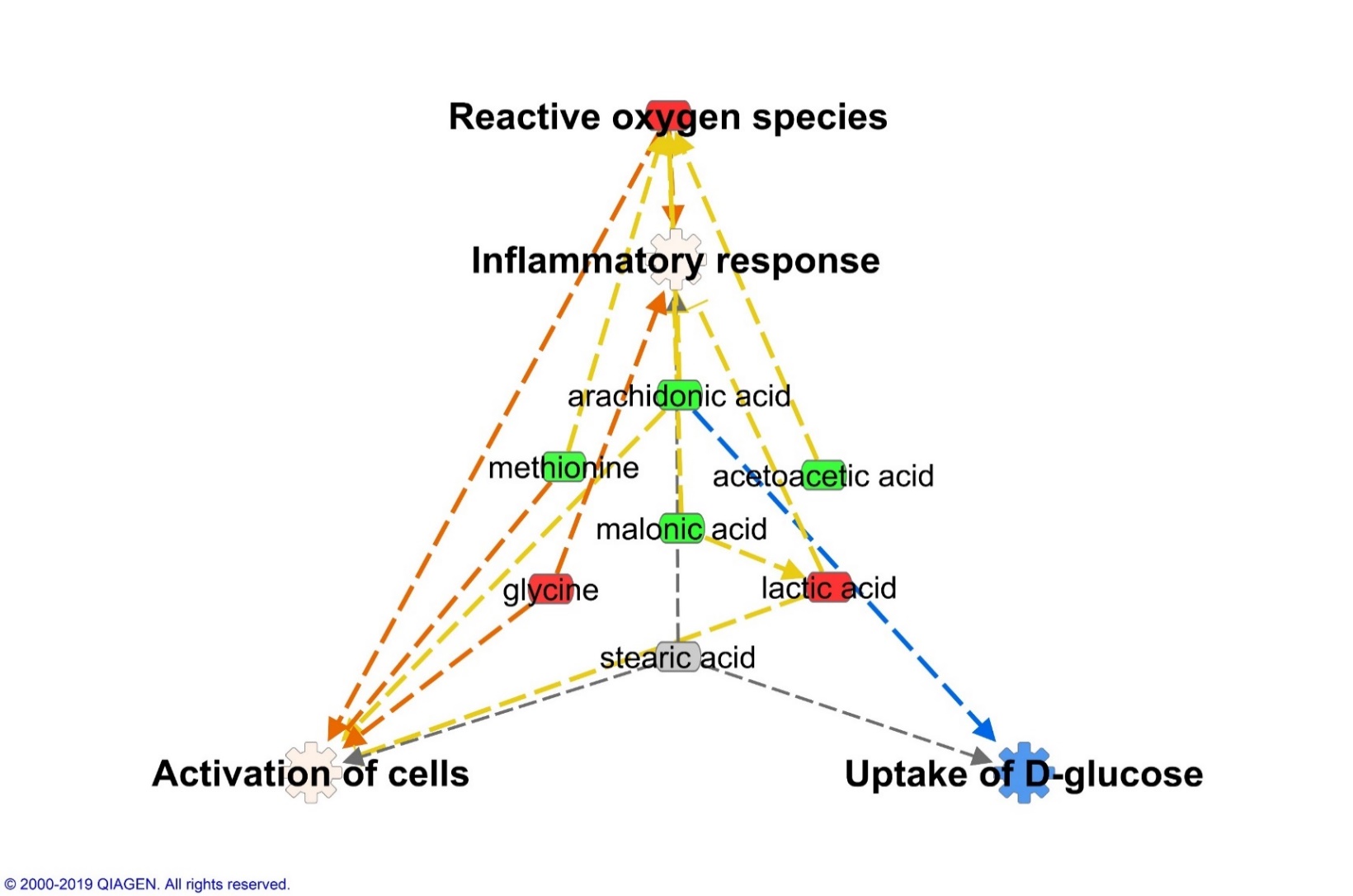


**Fig. S24**. Functional analysis of trimmed metabolic network with prediction of 0.01 µg/µl MNPs@SiO_2_(RITC) treated BV2 cells. Fold change ± 1.2 was used as cut off value. Details for shape and color are provided in Figs. S9 and S11.


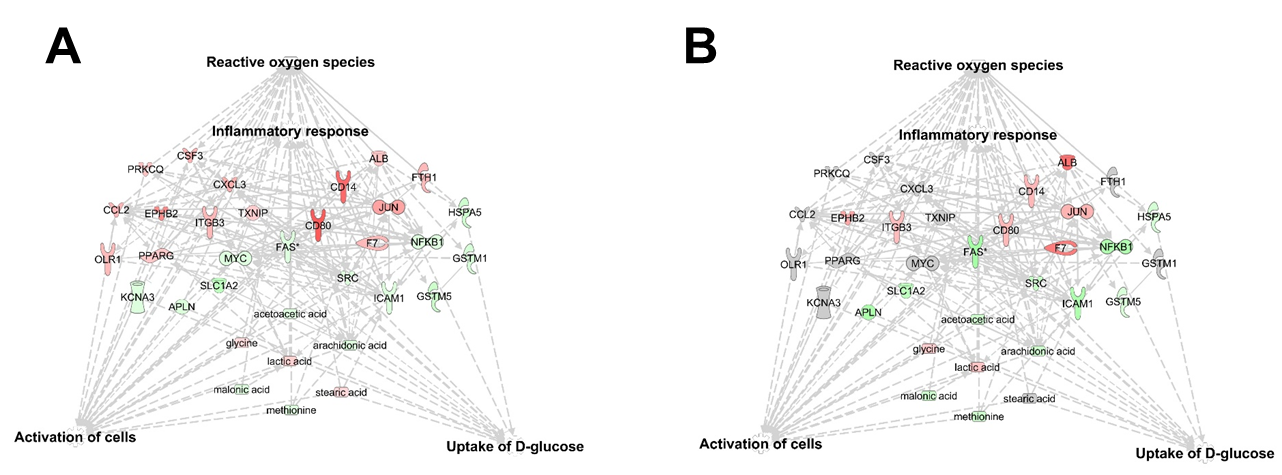


**Fig. S25**. Functional analysis of triple omics network of 0.1 **a** and 0.01 **b** µg/µl MNPs@SiO_2_(RITC) treated BV2 cells. Fold changes ± 1.5 for genes and proteins and ± 1.2 for metabolites were used as cut off value. Red and green areas indicate up- and downregulated factors, respectively. Symbols are described in the legend of Fig. S9.


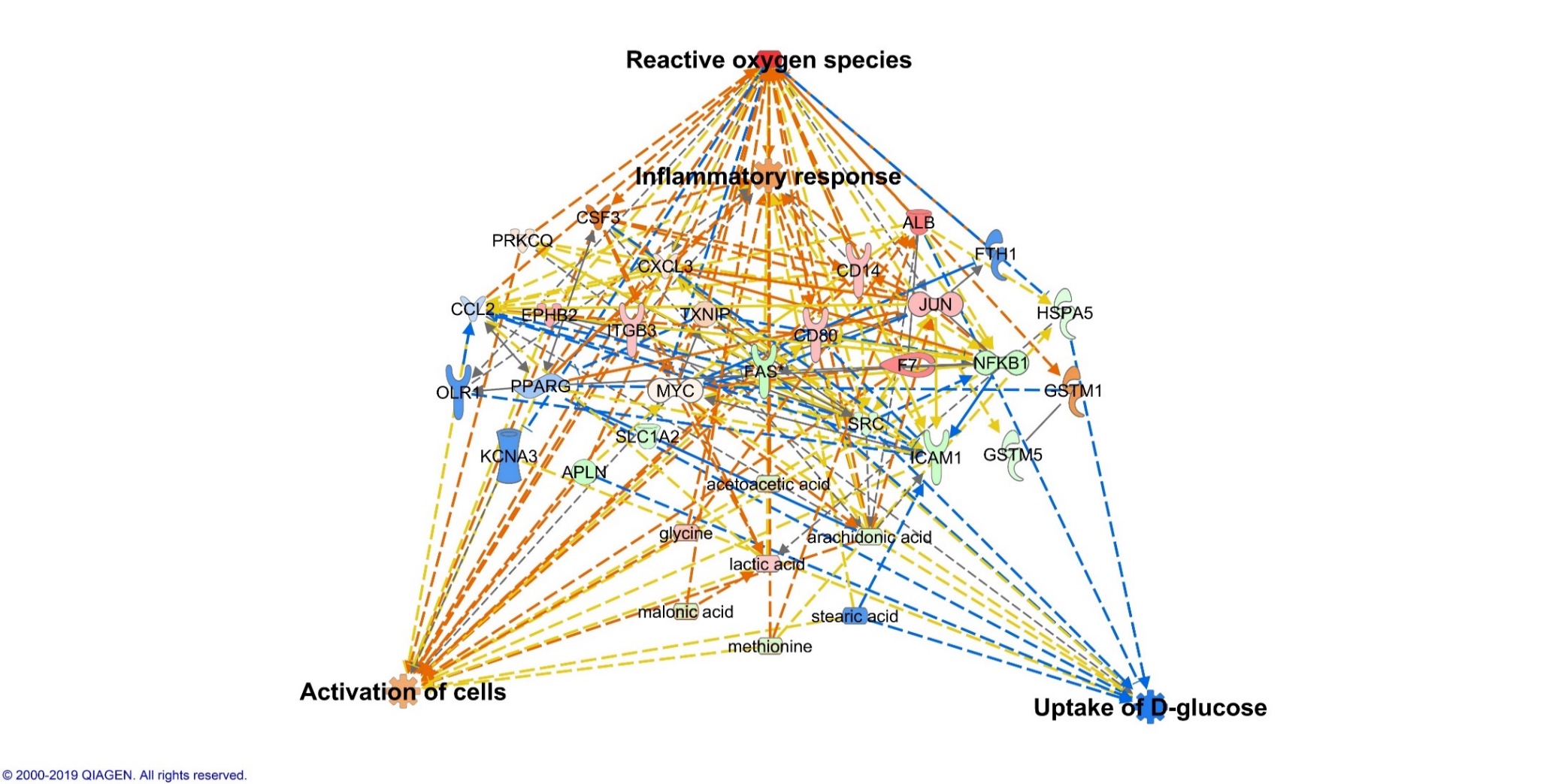


**Fig. S26**. Functional analysis of triple omics network with prediction of 0.01 µg/µl MNPs@SiO_2_(RITC) treated BV2 cells. Fold changes ± 1.5 for genes and proteins and ± 1.2 for metabolites were used as cut off value. Red and green areas indicate up- and downregulated factors, respectively. Orange and blue areas indicate prediction as activation and inhibition, respectively. Details for shape and color are provided in Figs. S9 and S11.


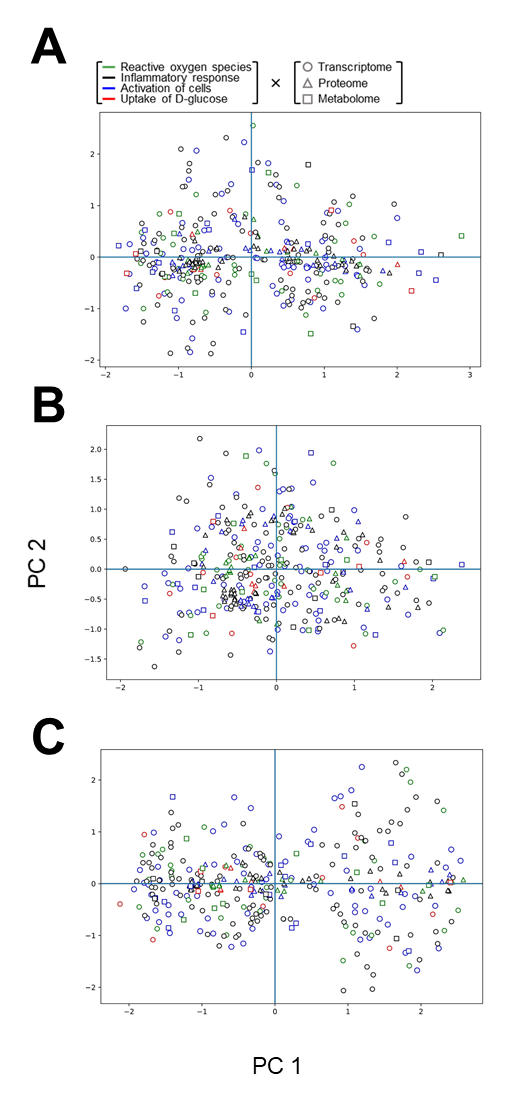


**Fig. S27**. PCA analysis for triple omics of non-treated control **a**, 0.01 **b** and 0.1 **c** µg/µl MNPs@SiO_2_(RITC) treated BV2 cells against 4 categories of biological functions.


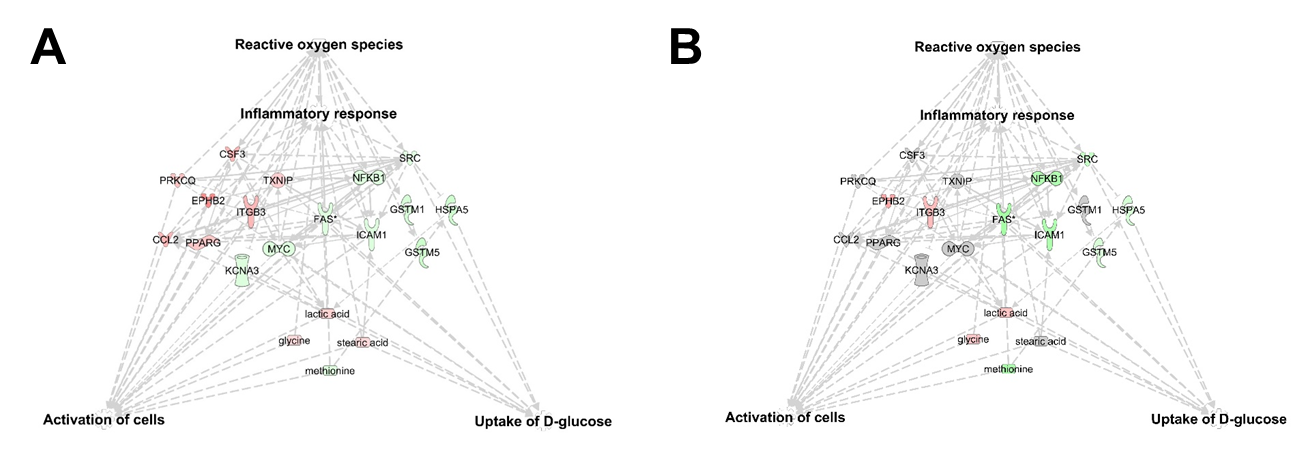


**Fig. S28**. Functional analysis of trimmed triple omics network of 0.1 **a** and 0.01 **b** µg/µl MNPs@SiO_2_(RITC) treated BV2 cells. Fold changes ± 1.5 for genes and proteins and ± 1.2 for metabolites were used as cut off value. Red and green areas indicate up- and downregulated factors, respectively. Symbols are described in the legend of Fig. S9.


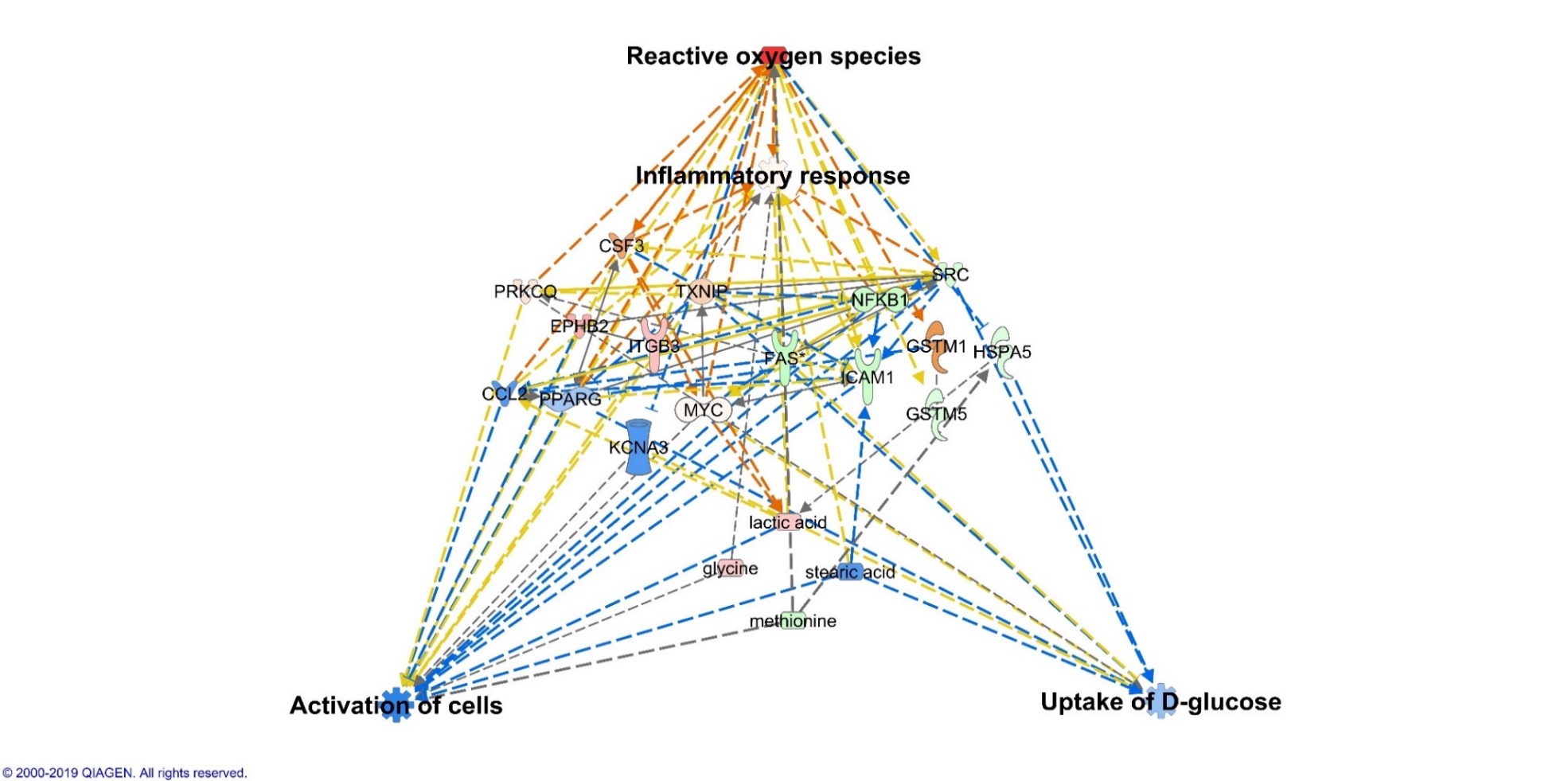


**Fig. S29**. Functional analysis of trimmed triple omics network with prediction of 0.01 µg/µl MNPs@SiO_2_(RITC) treated BV2 cells. Fold changes ± 1.5 for genes and proteins and ± 1.2 for metabolites were used as cut off value. Red and green areas indicate up- and downregulated factors, respectively. Orange and blue areas indicate prediction as activation and inhibition, respectively. Details for shape and color are provided in Figs. S9 and S11.


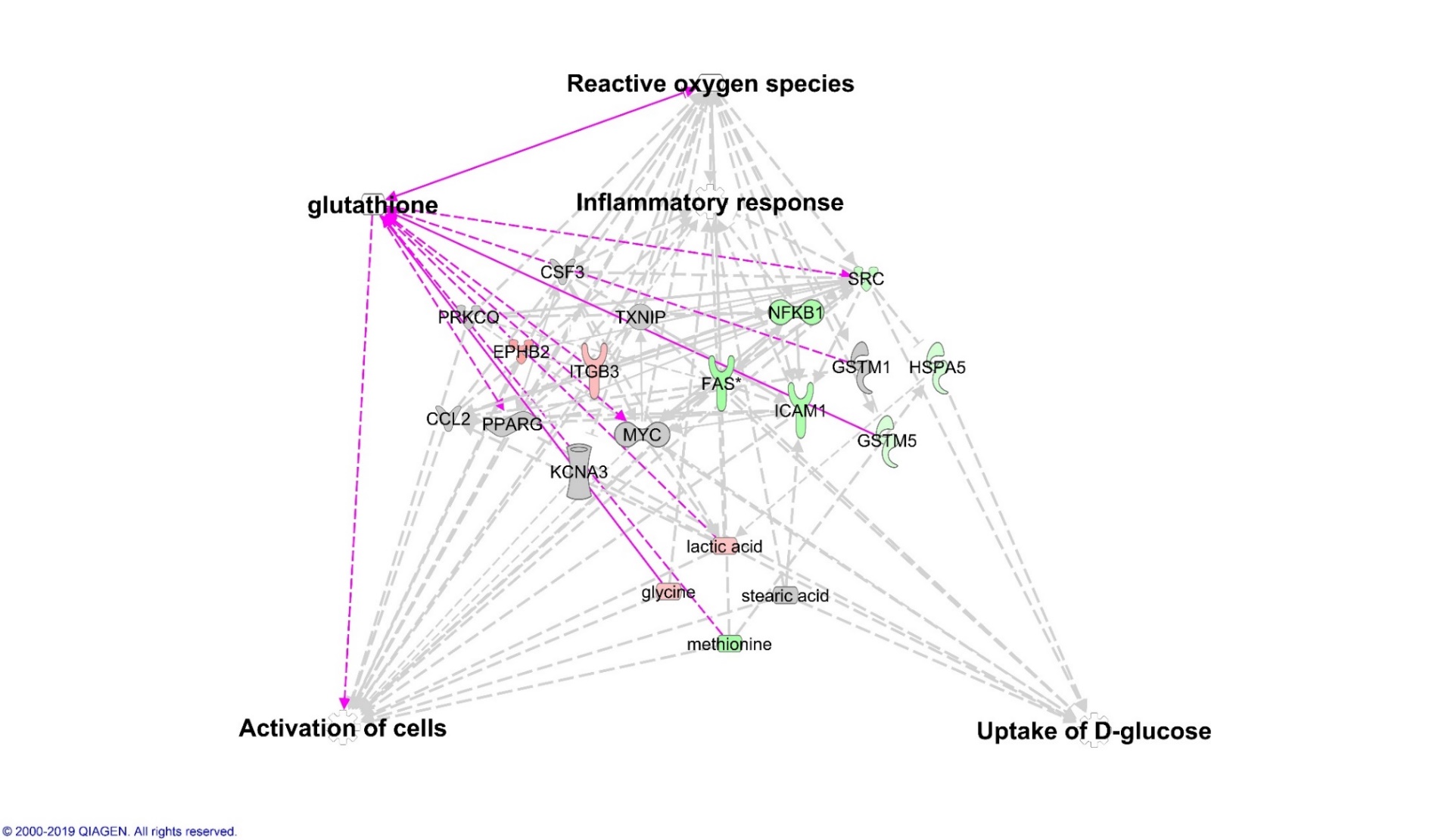


**Fig. S30**. Functional analysis of GSH-added trimmed triple omics network of 0.01 µg/µl MNPs@SiO_2_(RITC) treated BV2 cells. Fold changes ± 1.5 for genes and proteins and ± 1.2 for metabolites were used as cut off value. Red and green areas indicate up- and downregulated factors, respectively. Nodes, which is linked to glutathione, are highlighted with purple. Symbols are described in the legend of Fig. S9.


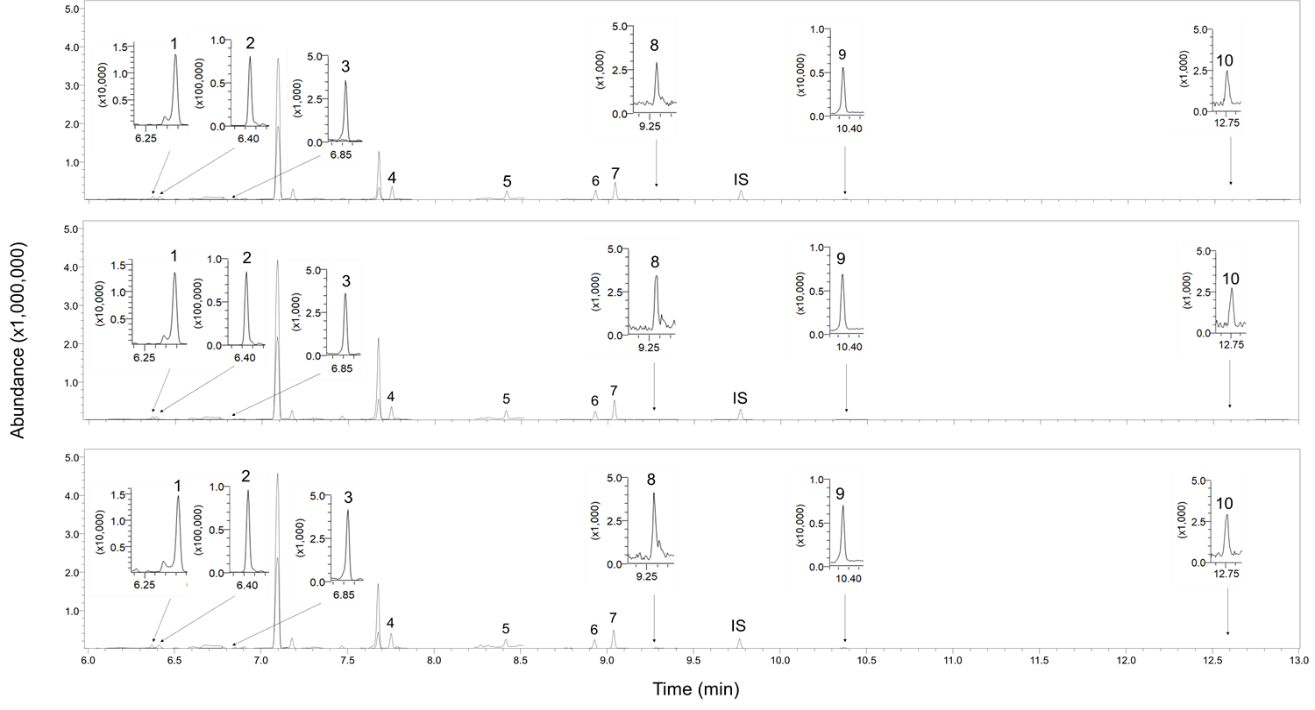


**Fig. S31.** Representative SIM chromatograms for 10 organic acid. 1: 3-Hydroxybutyric acid; 2: Pyruvic acid; 3: Acetoacetic acid; 4: Glycolic acid; 5: Malonic acid; 6: Succinic acid; 7: Fumaric acid; 8: Oxaloacetic acid; 9: Malic acid; 10: Citric acid. Top: control, middle: 0.01 µg/µl MNPs@SiO_2_(RITC) treated BV2 cells, bottom: 0.1 µg/µl MNPs@SiO_2_(RITC) treated BV2 cells.


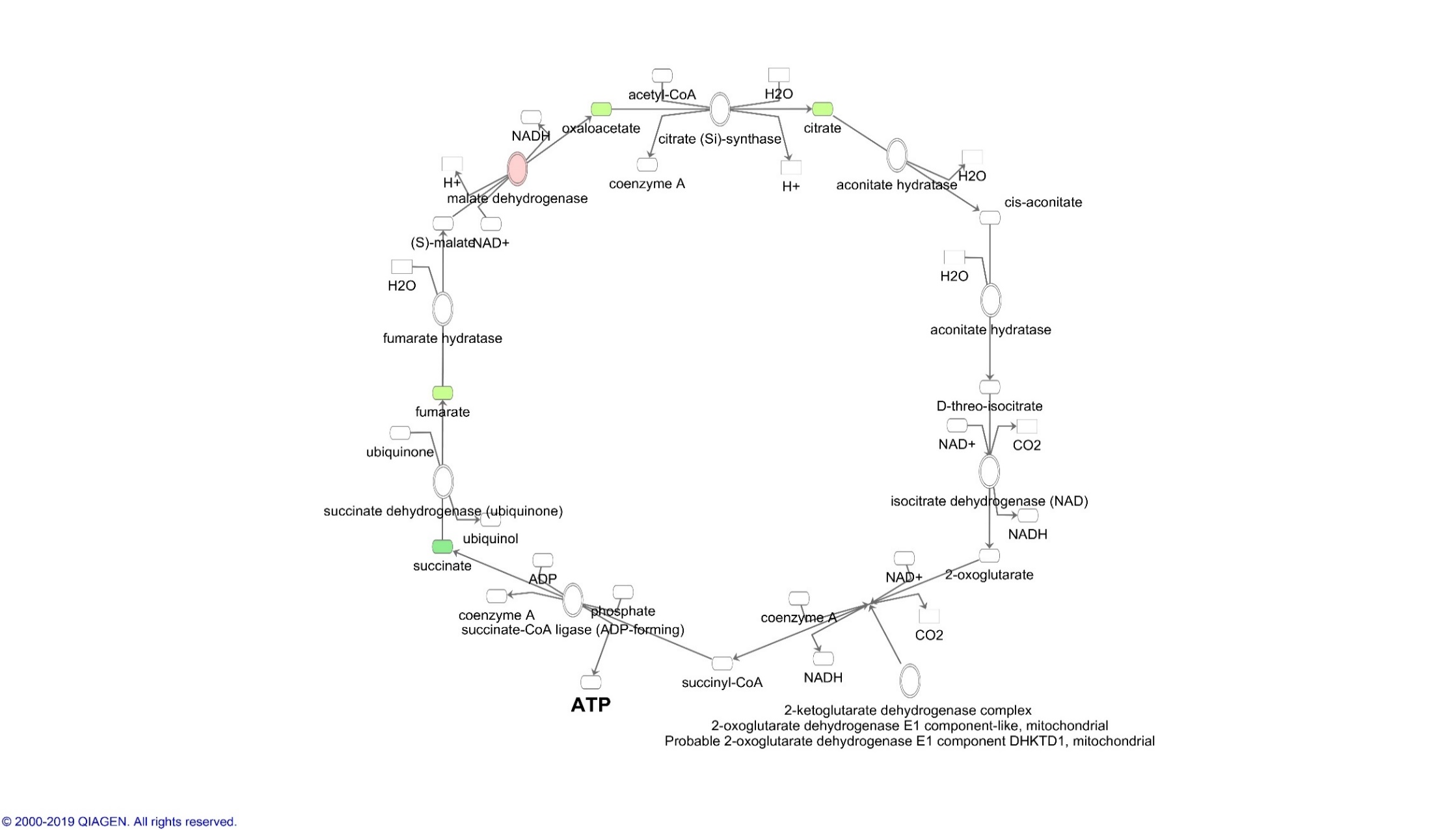


**Fig. S32.** Canonical pathway analysis for TCA cycle with triple omics. Fold changes ± 1.5 for genes and proteins and ± 1.2 for metabolites were used as cut off value. Red and green areas indicate factors that were increased and decreased compared to the untreated control group, respectively. Symbols are described in the legend of Fig. S9.


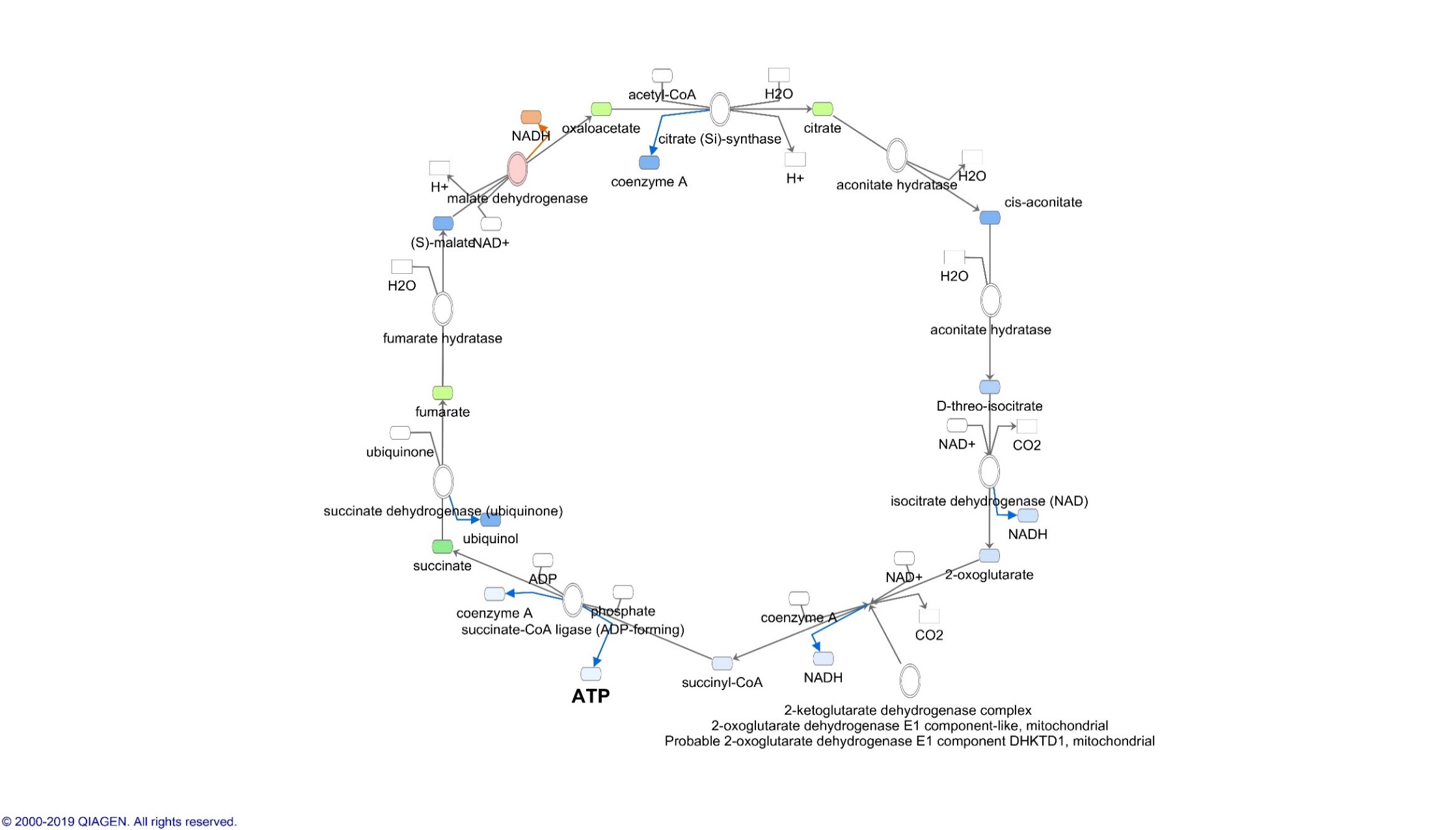


**Fig. S33.** Canonical pathway analysis for TCA cycle with triple omics prediction. Fold changes ± 1.5 for genes and proteins and ± 1.2 for metabolites were used as cut off value. Red and green areas indicate factors that were increased and decreased compared to the untreated control group, respectively. Orange and blue areas indicate prediction as activation and inhibition, respectively. Details for shape and color are provided in Figs. S9 and S11.

**
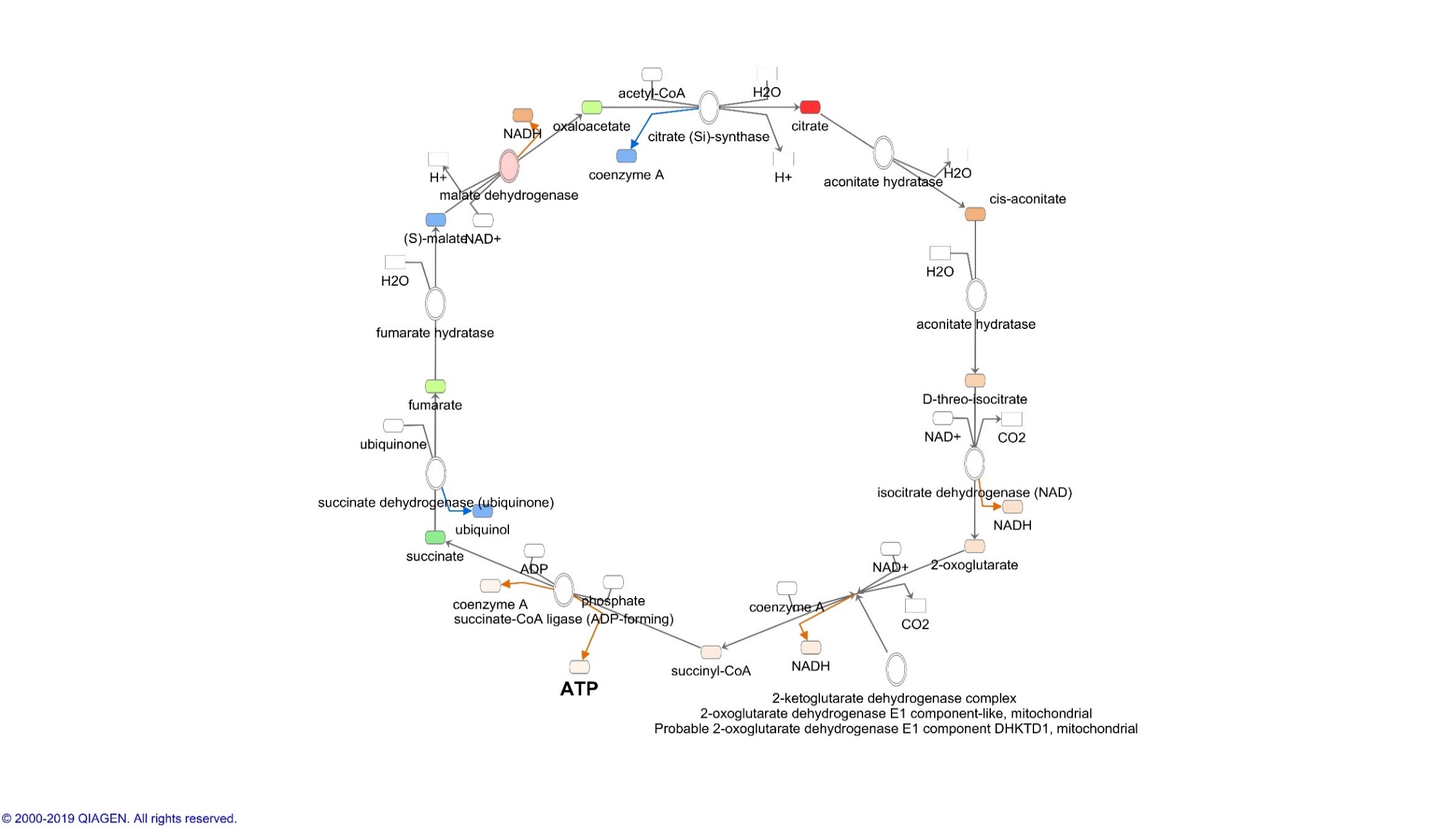
**

**Fig. S34.** Canonical pathway analysis for TCA cycle with triple omics prediction in citrate supplementation. Fold changes ± 1.5 for genes and proteins and ± 1.2 for metabolites were used as cut off value. Red and green areas indicate factors that were increased and decreased compared to the untreated control group, respectively. Orange and blue areas indicate prediction as activation and inhibition, respectively. Details for shape and color are provided in Figs. S9 and S11.


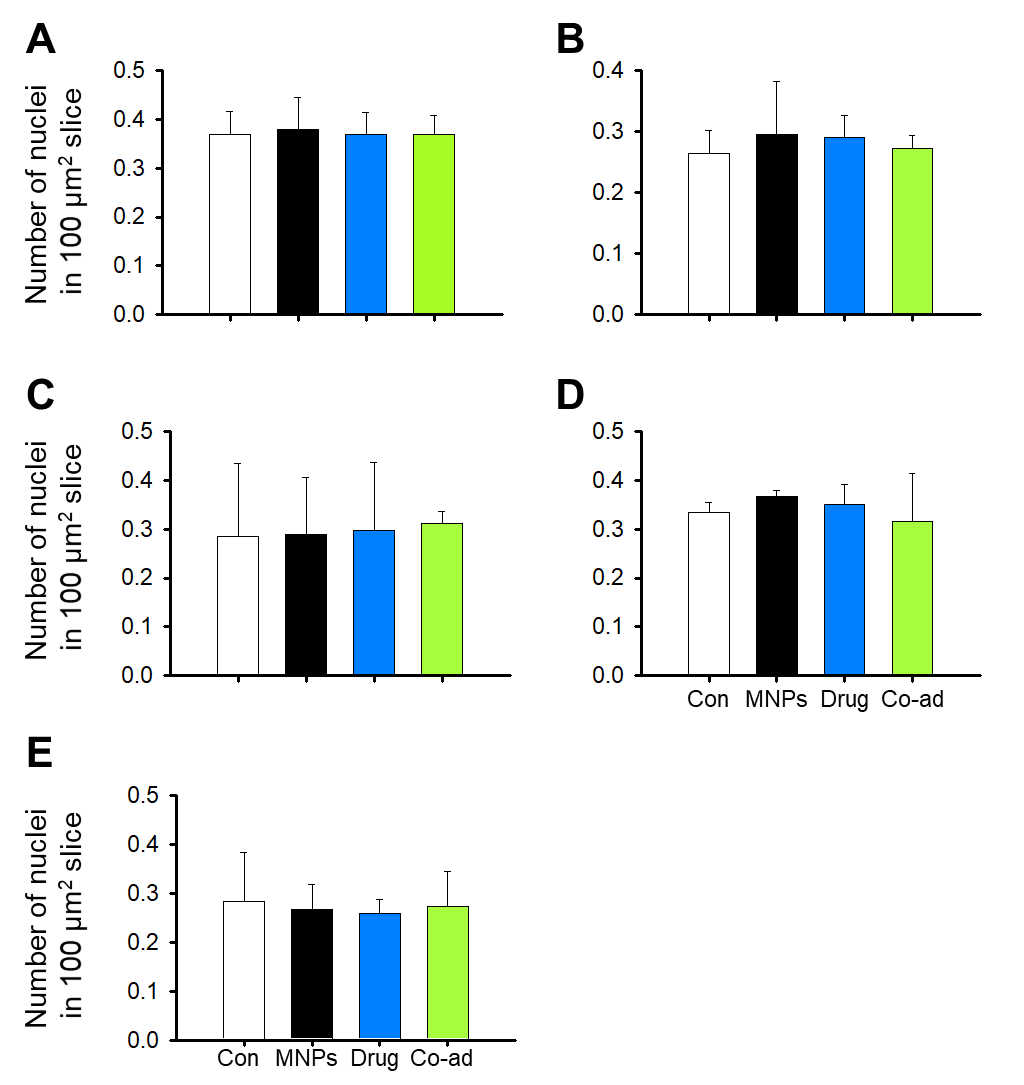


**Fig. S35.** Quantification of the number of nuclei per 100 μm^2^ brain slice of cortex **a**, striatum **b**, hippocampus **c**, thalamus **d**, and cerebellum **e**. Total 150,000 μm^2^ per each brain part were analyzed. Data represent means ± standard error of 4 samples per group. Con = control; MNPs = MNPs@SiO_2_(RITC) treated mice; Drug = GSH and citric acid treated mice; Co-ad = MNPs@SiO_2_(RITC) plus combination of GSH and citric acid co-administered mice.


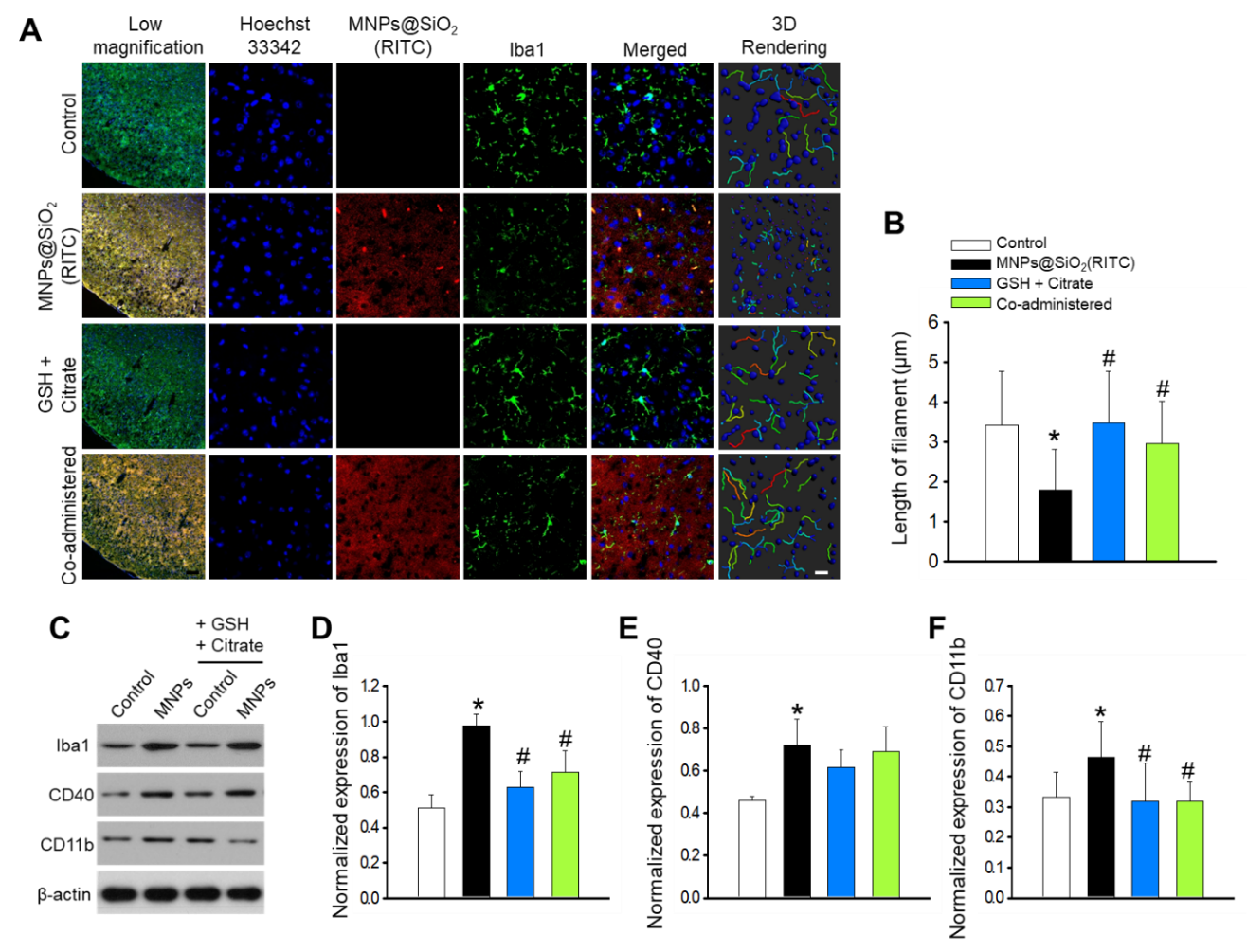


**Fig. S36.** Evaluation of MNPs@SiO_2_(RITC)-induced microglial activation and effect of GSH and citric acid in the mouse cortex. **a** Immunohistochemical analysis of the cortical regions of mice. Low-magnification images are merged with florescence of Hoechst 33342 (blue), MNPs@SiO_2_(RITC) (red), and Iba1 (green) to show region-specific structure and distribution of MNPs@SiO_2_(RITC). Black scale bar = 100 μm. Magnified images are separated into Hoechst 33342 (blue), MNPs@SiO_2_(RITC) (red), and Iba1 (green), and Iba1-based 3D rendering images. White scale bar = 10 µm. **b** Determined length of the filament from 3D rendering images. **p* < 0.05 vs. control, ^#^*p* < 0.05 vs. MNPs@SiO_2_(RITC)-treated mice. **c** Representative immunoblotting images related to microglia activation. β-Actin served as an internal control. Normalized expression of Iba1 **d**, CD40 **e**, and CD11b **f**. Data represent means ± standard error of three independent experiments. **p* < 0.05 vs. control, ^#^*p* < 0.05 vs. MNPs@SiO_2_(RITC)-treated mice.


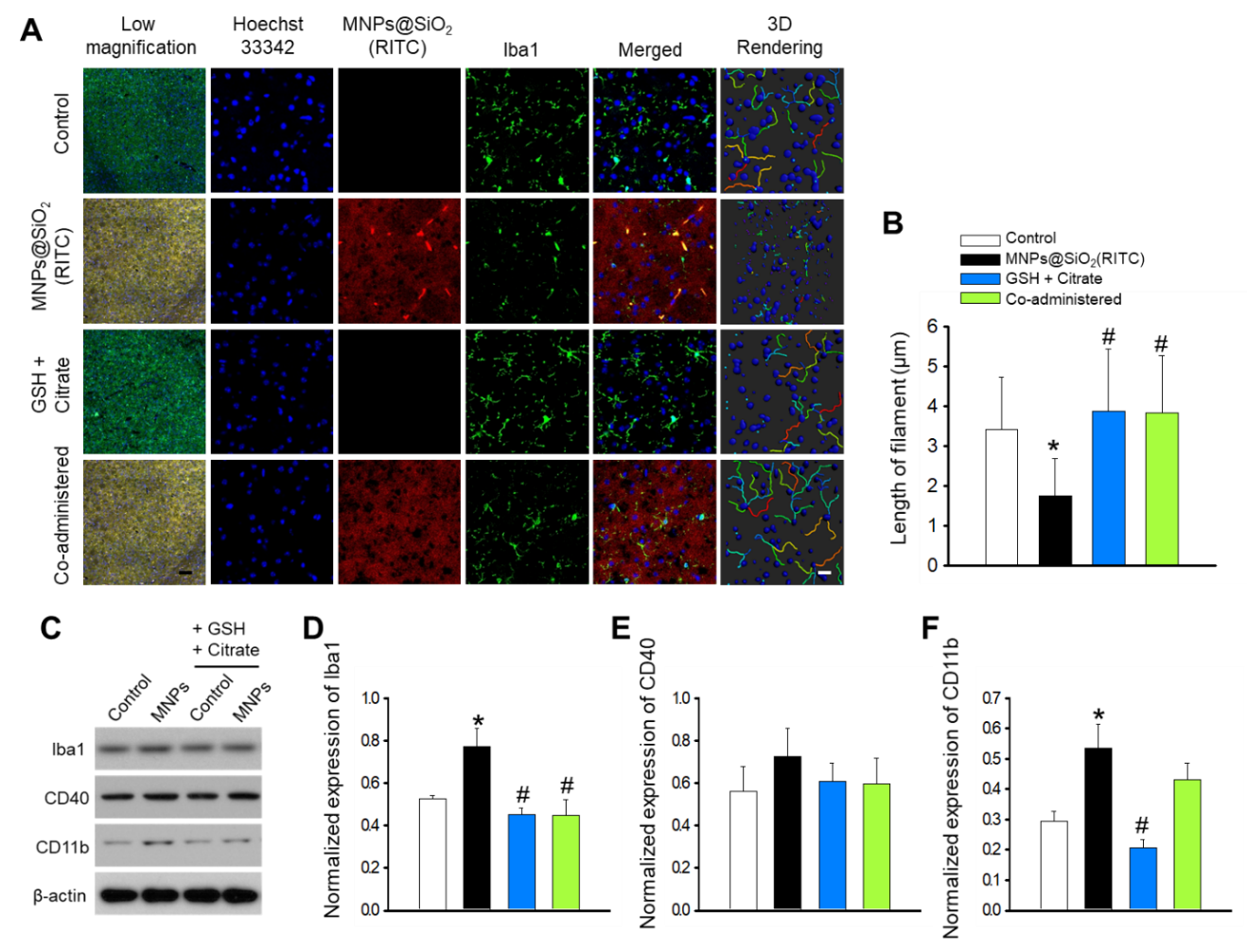


**Fig. S37.** Evaluation of MNPs@SiO_2_(RITC)-induced microglial activation and effect of GSH and citric acid in the mouse striatum. **a** Immunohistochemical analysis of the striatal regions of mice. Low-magnification images are merged with florescence of Hoechst 33342 (blue), MNPs@SiO_2_(RITC) (red), and Iba1 (green) to show region-specific structure and distribution of MNPs@SiO_2_(RITC). Black scale bar = 100 μm. Magnified images are separated into Hoechst 33342 (blue), MNPs@SiO_2_(RITC) (red), and Iba1 (green), and Iba1-based 3D rendering images. White scale bar = 10 µm. **b** Determined length of the filament from 3D rendering images. **p* < 0.05 vs. control, ^#^*p* < 0.05 vs. MNPs@SiO_2_(RITC)-treated mice. **c** Representative immunoblotting images related to microglia activation. β-Actin served as an internal control. Normalized expression of Iba1 **d**, CD40 **e**, and CD11b **f**. Data represent means ± standard error of three independent experiments. **p* < 0.05 vs. control, ^#^*p* < 0.05 vs. MNPs@SiO_2_(RITC)-treated mice.


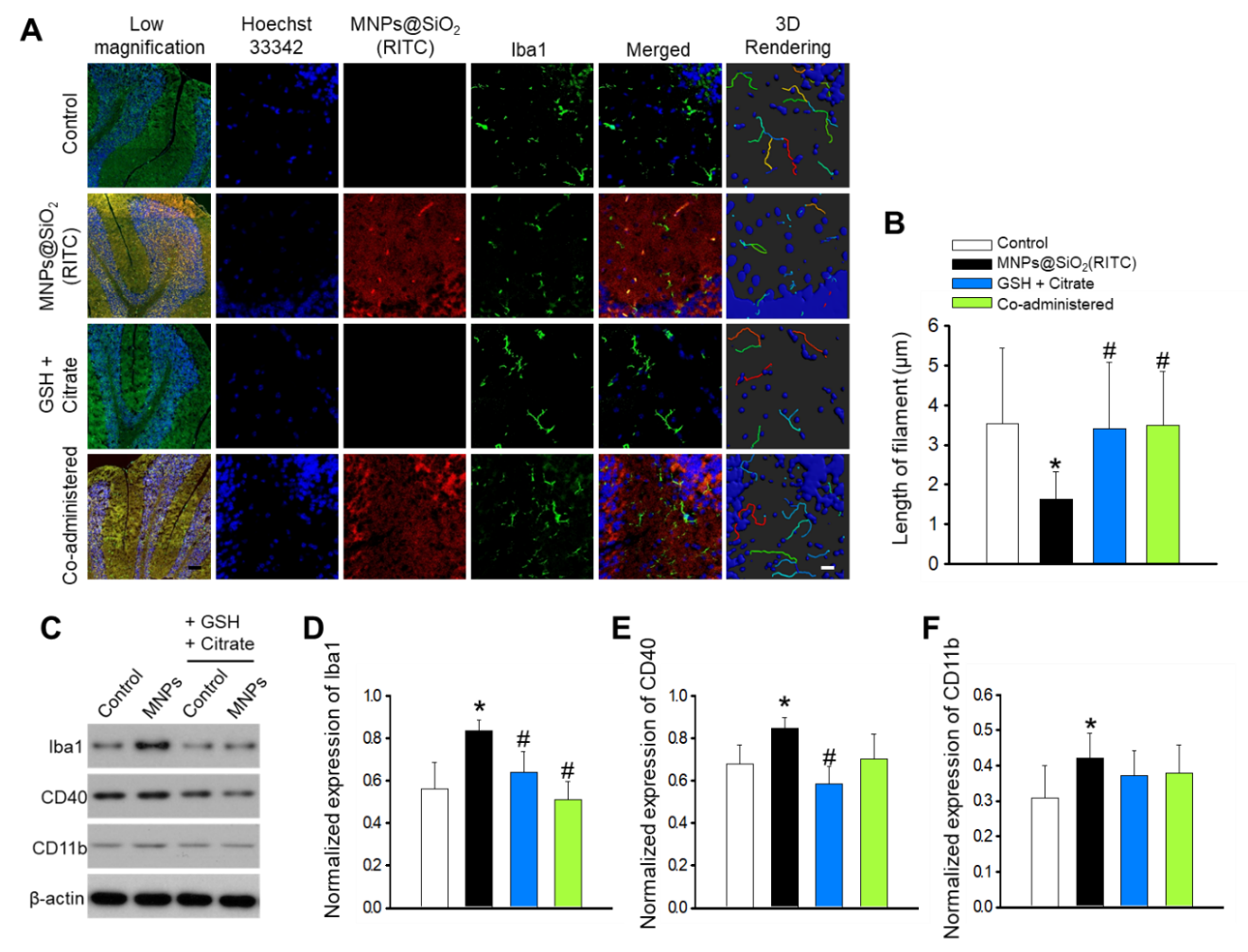


**Fig. S38.** Evaluation of MNPs@SiO_2_(RITC)-induced microglial activation and effect of GSH and citric acid in the mouse cerebellum. **a** Immunohistochemical analysis of the cerebellar regions of mice. Low-magnification images are merged with florescence of Hoechst 33342 (blue), MNPs@SiO_2_(RITC) (red), and Iba1 (green) to show region-specific structure and distribution of MNPs@SiO_2_(RITC). Black scale bar = 100 μm. Magnified images are separated into Hoechst 33342 (blue), MNPs@SiO_2_(RITC) (red), and Iba1 (green), and Iba1-based 3D rendering images. White scale bar = 10 µm. **b** Determined length of the filament from 3D rendering images. **p* < 0.05 vs. control, ^#^*p* < 0.05 vs. MNPs@SiO_2_(RITC)-treated mice. **c** Representative immunoblotting images related to microglia activation. β-Actin served as an internal control. Normalized expression of Iba1 **d**, CD40 **e**, and CD11b **f**. Data represent means ± standard error of three independent experiments. **p* < 0.05 vs. control, ^#^*p* < 0.05 vs. MNPs@SiO_2_(RITC)-treated mice.


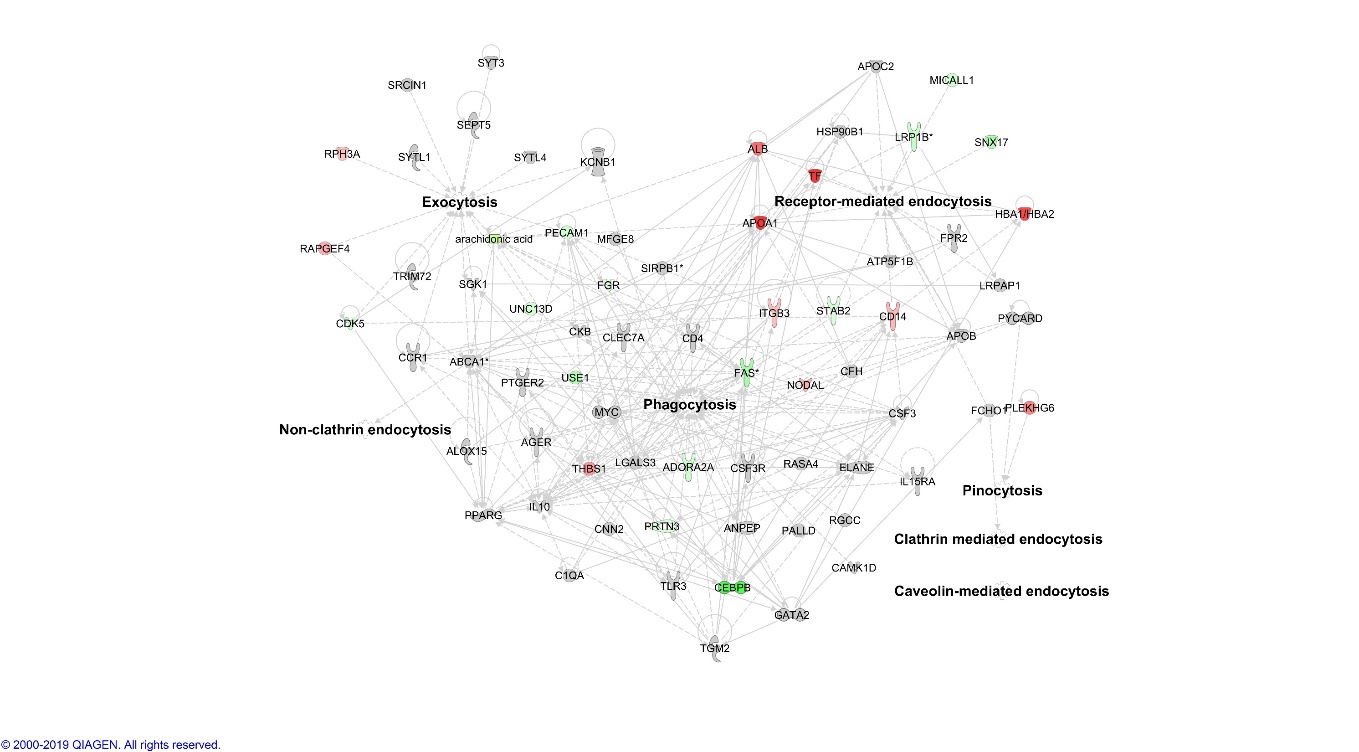


**Fig. S39**. Functional analysis of endocytosis and exocytosis related triple omics network of 0.01 µg/µl MNPs@SiO_2_(RITC) treated BV2 cells. Fold changes ± 1.5 for genes and proteins and ± 1.2 for metabolites were used as cut off value. Red and green areas indicate up- and down-regulated factors, respectively. Symbols are described in the legend of Fig. S9.


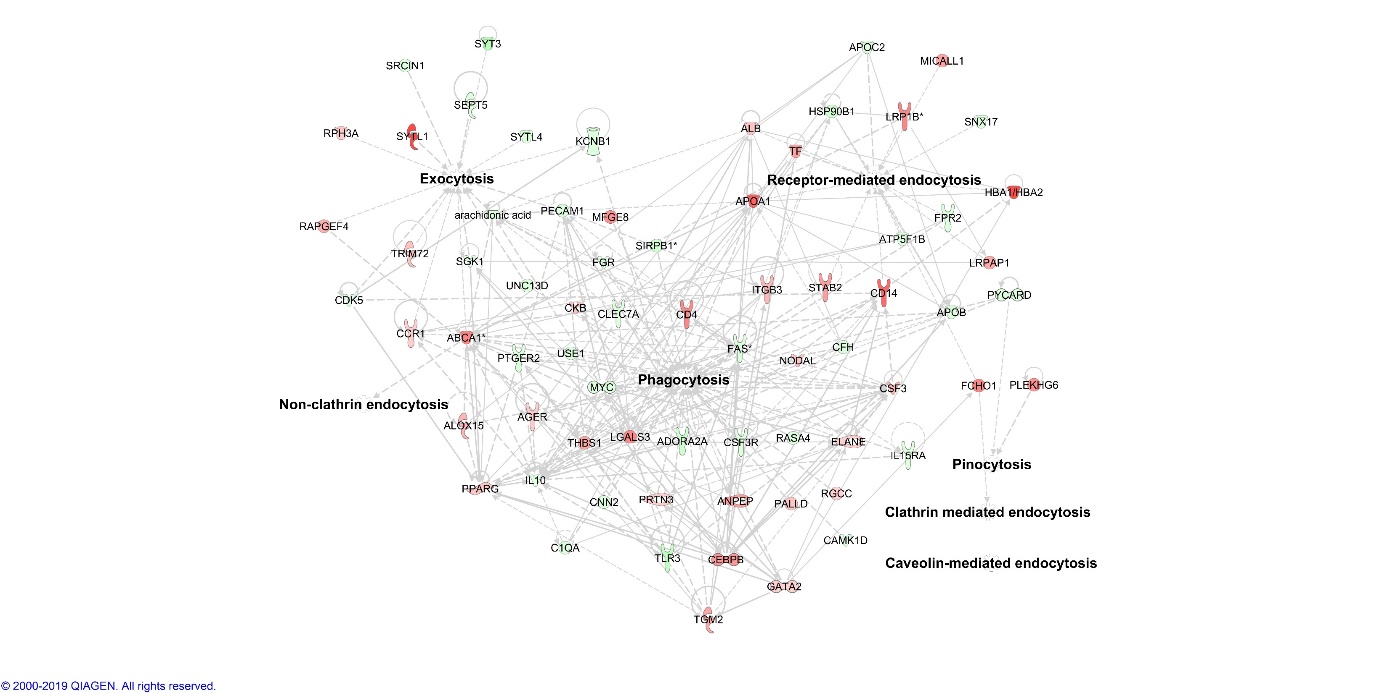


**Fig. S40**. Functional analysis of endocytosis and exocytosis related triple omics network of 0.1 µg/µl MNPs@SiO_2_(RITC) treated BV2 cells. Fold changes ± 1.5 for genes and proteins and ± 1.2 for metabolites were used as cut off value. Red and green areas indicate up- and downregulated factors, respectively. Symbols are described in the legend of Fig. S9.


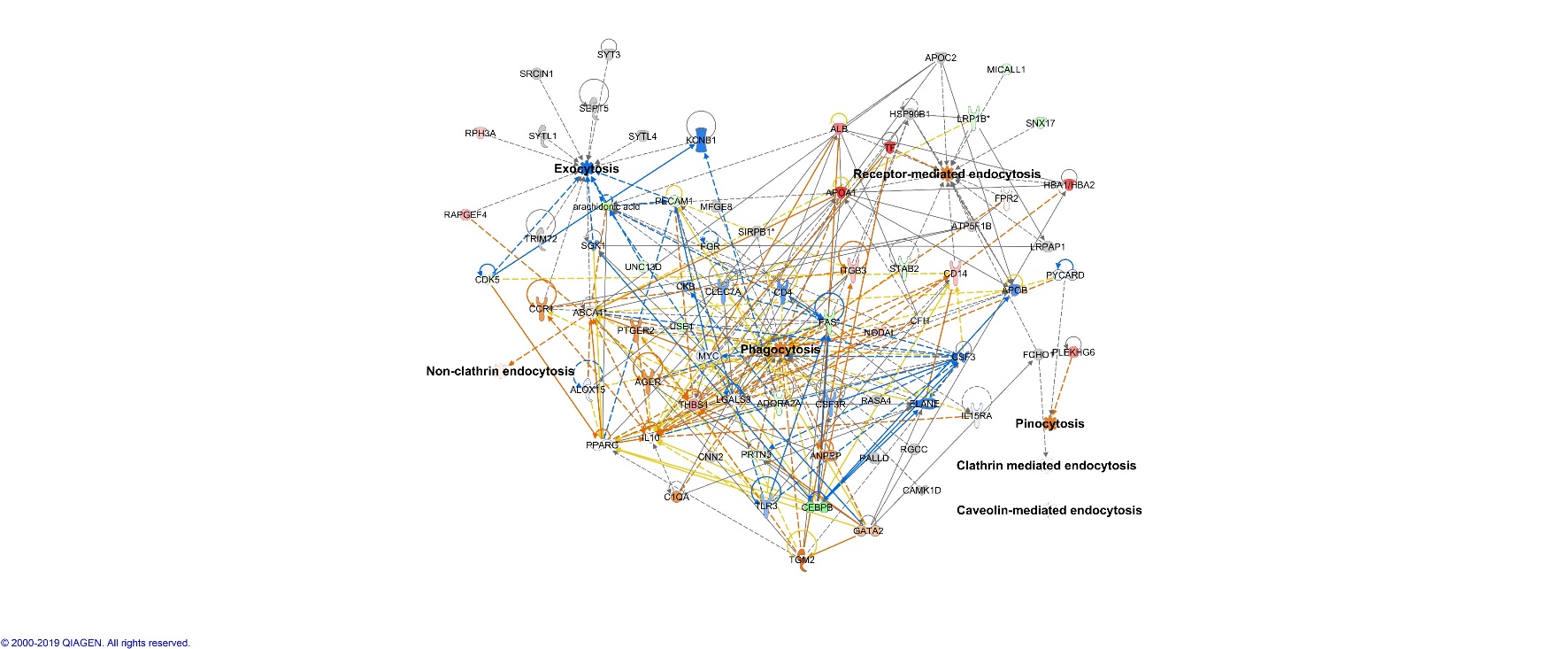


**Fig. S41.** Functional analysis of endocytosis and exocytosis related triple omics network prediction of 0.01 µg/µl MNPs@SiO_2_(RITC) treated BV2 cells Fold changes ± 1.5 for genes and proteins and ± 1.2 for metabolites were used as cut off value. Red and green areas indicate factors that were increased and decreased compared to the untreated control group, respectively. Orange and blue areas indicate prediction as activation and inhibition, respectively. Details for shape and color are provided in Figs. S9 and S11.


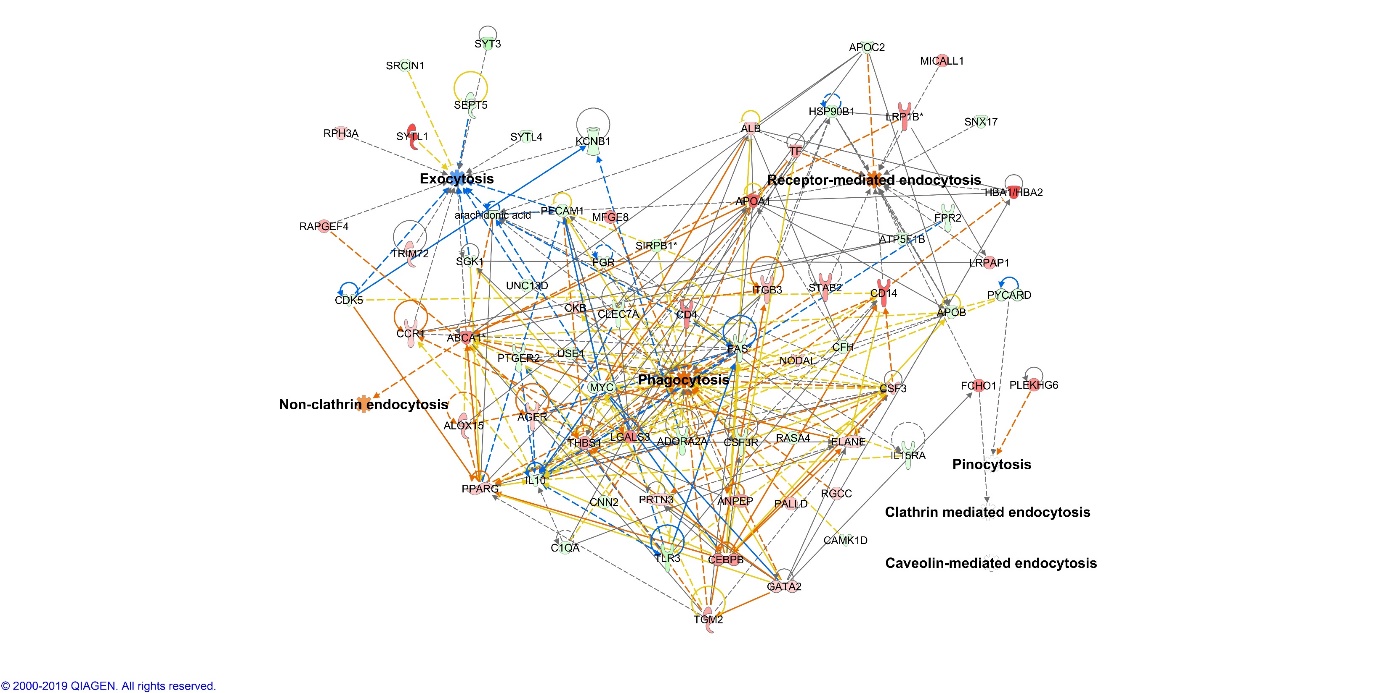


**Fig. S42.** Functional analysis of endocytosis and exocytosis related triple omics network prediction of 0.1 µg/µl MNPs@SiO_2_(RITC) treated BV2 cells Fold changes ± 1.5 for genes and proteins and ± 1.2 for metabolites were used as cut off value. Red and green areas indicate factors that were increased and decreased compared to the untreated control group, respectively. Orange and blue areas indicate prediction as activation and inhibition, respectively. Details for shape and color are provided in Figs. S9 and S11.

**References**

1. Carvalho PC, Xu T, Han X, Cociorva D, Barbosa VC, Yates JR, III. YADA: a tool for taking the most out of high-resolution spectra. Bioinformatics. 2009, 25:2734-2736.

2. Tabb DL, McDonald WH, Yates JR, 3rd. DTASelect and Contrast: tools for assembling and comparing protein identifications from shotgun proteomics. J Proteome Res. 2002, 1:21-26.

3. Raso C, Cosentino C, Gaspari M, Malara N, Han X, McClatchy D, Park SK, Renne M, Vadala N, Prati U, et al. Characterization of breast cancer interstitial fluids by TmT labeling, LTQ-Orbitrap Velos mass spectrometry, and pathway analysis. J Proteome Res. 2012, 11:3199-3210.

4. Trapnell C, Roberts A, Goff L, Pertea G, Kim D, Kelley DR, Pimentel H, Salzberg SL, Rinn JL, Pachter L. Differential gene and transcript expression analysis of RNA-seq experiments with TopHat and Cufflinks. Nat Protoc. 2012, 7:562-578.

5. Paik MJ, Lee HJ, Kim KR. Simultaneous retention index analysis of urinary amino acids and carboxylic acids for graphic recognition of abnormal state. J Chromatogr B Analyt Technol Biomed Life Sci. 2005, 821:94-104.

6. Paik MJ, Kim KR. Sequential ethoxycarbonylation, methoximation and tert-butyldimethylsilylation for simultaneous determination of amino acids and carboxylic acids by dual-column gas chromatography. J Chromatogr A. 2004, 1034:13-23.

7. Eppig JT, Richardson JE, Kadin JA, Ringwald M, Blake JA, Bult CJ. Mouse Genome Informatics (MGI): reflecting on 25 years. Mamm Genome. 2015, 26:272-284.
